# The global ocean size spectrum from bacteria to whales

**DOI:** 10.1126/sciadv.abh3732

**Published:** 2021-11-10

**Authors:** Ian A. Hatton, Ryan F. Heneghan, Yinon M. Bar-On, Eric D. Galbraith

**Affiliations:** 1 Max Planck Institute for Mathematics in the Sciences, Leipzig 04103, Germany.; 2 Institut de Ciència i Tecnologia Ambientals (ICTA), Universitat Autonoma de Barcelona, Barcelona, Spain.; 3 School of Mathematical Sciences, Queensland University of Technology, Brisbane, QD 4000, Australia.; 4 Department of Plant and Environmental Sciences, Weizmann Institute of Science, 76100 Rehovot, Israel.; 5 Department of Earth and Planetary Sciences, McGill University, Montreal, QC H3A 0E8, Canada.

## Abstract

It has long been hypothesized that aquatic biomass is evenly distributed among logarithmic body mass size classes. Although this community structure has been observed regionally, mostly among plankton groups, its generality has never been formally tested across all marine life over the global ocean, nor have the impacts of humans on it been globally assessed. Here, we bring together data at the global scale to test the hypothesis from bacteria to whales. We find that biomass within most order of magnitude size classes is indeed remarkably constant, near 1 gigatonne (Gt) wet weight (10^15^ g), but bacteria and large marine mammals are markedly above and below this value, respectively. Furthermore, human impacts appear to have significantly truncated the upper one-third of the spectrum. This dramatic alteration to what is possibly life’s largest-scale regularity underscores the global extent of human activities.

## Teaser

Human activity has altered one of life’s largest-scale patterns: a global power law size distribution from bacteria to whales.

## INTRODUCTION

In 1972, Sheldon *et al.* (*1*) published measurements of marine plankton abundance spanning about six orders of magnitude in body mass (from ~0.6 to 100 μm in body length), collected at approximately 80 Atlantic and Pacific stations in a circumnavigation of the Americas. At each station, the total biomass of all individuals was approximately evenly distributed across logarithmic size classes (*1*). On the basis of these planktonic observations, they boldly hypothesized that “to a first approximation, roughly equal concentrations of material occur at all particle sizes within the range from 1 μm to about 10^6^ μm, i.e., from bacteria to whales.” Although Sheldon *et al.* (*1*) were focused on the distribution of biomass at the regional scale, their study had immediate global implications. Yet, this extraordinary hypothesis has never been formally tested globally across the astronomical range in body masses it encompasses nor has its possible alteration by human impacts been examined at the whole-ocean scale.

Since Sheldon *et al.*’s seminal work (*1*), the distribution that results from aggregating individuals, regardless of species identity, into size bins has become known as the size spectrum or Sheldon spectrum, among other names (*2*). Sheldon *et al.*’s hypothesis has been widely validated at local and regional scales, mostly among pelagic plankton groups (*1*–*6*), but occasionally extending up to fish (*7*, *8*), as well as in freshwater systems (*8*–*11*). These studies have often reported notable similarity in the exponents of these distributions (fig. S2 and table S3) (*1*–*3*, *5*, *7*, *12*). Although there are many ways to represent the size spectrum (*2*), these exponents are typically near −1 for the relation between the logarithm of numerical abundance across logarithmic size classes, equivalent to an exponent near 0 for biomass across log size classes (*2*) (Materials and Methods).

These empirical findings have inspired a rich literature on size spectrum theory [see recent reviews; (*2*, *12*–*15*)]. Existing explanations for the size spectrum are predominantly based on variations of predator-prey interactions and tend to rely on a combination of two or more lower level factors to account for the distribution of biomass across size classes. These factors include metabolism (*16*–*23*), trophic growth efficiency (*19*–*25*), encounter rates or consumption (*18*, *26*–*28*), predator-prey mass ratios (*19*–*24*, *26*, *28*–*30*), rates of growth (*16*, *17*, *19*, *22*, *25*–*30*), birth or reproduction (*26*–*28*), and mortality (*16*, *17*, *25*–*30*). Many of these variables exhibit robust allometric scaling relations with body size (*31*–*34*), but which combination of variables ultimately dominate the maintenance of the size-spectrum across the diversity of marine taxa remains an open question. More generally, a great diversity of adaptive traits, from life history and resource encounter strategies to mobility and sensory ability, depends on organisms “being the right size” (*14*, *31*, *33*, *34*). This suggests that not all size classes are created equal and that certain sizes should be selectively advantaged or disadvantaged, challenging the idea of an evenly distributed size spectrum. Knowing the community structure across the full size range of marine life is thus key to strengthening size spectrum theory and is needed for a broader understanding of biosphere functioning and human impacts on the global ocean (*13*, *15*, *35*).

Despite nearly 50 years of empirical and theoretical work, research has been dominated by regional studies and has been limited to much smaller size ranges than the 23 orders of magnitude originally conjectured [but see (*36*)]. Empirical size spectra have typically averaged a range in body mass of six orders of magnitude and have not exceeded 16 (*5*, *7*, *8*, *10*) (table S3). A major challenge to comparisons over these disparate size scales is posed by the extremely different spatial scales over which measurements must be made. Whereas bacteria and small plankton can be estimated in a small water sample, the largest fish and mammals can actively range over thousands of kilometers, and only the global scale unambiguously captures the full spatial range of all marine organisms. Furthermore, although human activities are known to have locally altered the shape of many size spectra (*2*, *12*, *15*, *37*, *38*), the extent to which these activities may have affected the whole-ocean size spectrum has not been investigated.

**Fig. 1.**
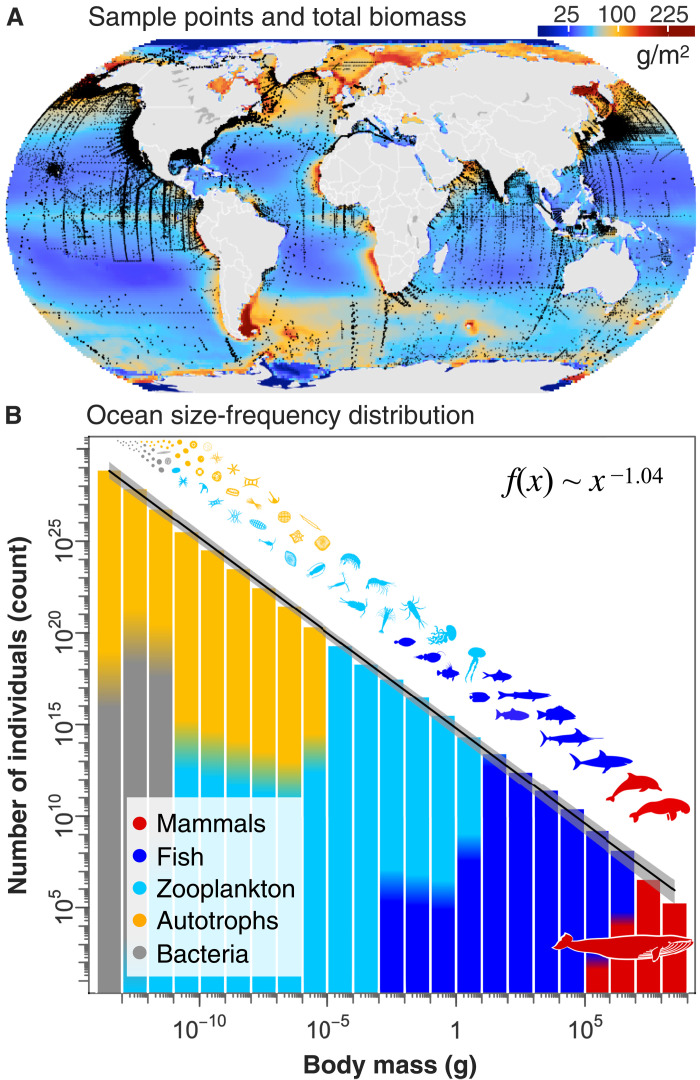
The global ocean size spectrum. (**A**) The black mapped points are *n* = 226,405 sample locations for measurements of heterotrophic bacteria and zooplankton. Autotrophs were estimated from satellite imagery of surface chlorophyll and fish from global process models constrained by catch data. Marine mammals are estimated from species global population estimates, and their biomass is not included on the map. Biomass (g/m^2^; wet weight) of each group is summed over all groups in each 1° region of the ocean (only biomass in the upper 200 m is shown here). (**B**) Total ocean biomass (wet weight) is partitioned across relevant size classes (g, wet weight) for each group to estimate the global size spectrum. This is shown as the total number of individuals in each order of magnitude size class over the ocean’s epipelagic and continental shelves (upper ~200 m), giving an exponent of −1.04 (95% CI: −1.05 to −1.02). The gray confidence band includes biomass uncertainty in each size class (Fig. 2 A) and uncertainty in the size distribution of each group (Fig. 2C, i). Bin colors show the relative fraction of each group on a linear axis [no relation to *y* axis or to the biomass in (A)]. Further details are in Materials and Methods.

Here, we make use of advances in global ocean observation and recent meta-analyses to test Sheldon’s original hypothesis from bacteria to whales at the global scale. We evaluate this hypothesis in a “pristine” state, before industrial-scale human capture of fish and marine mammals (pre-1850), based on a combination of marine ecosystem models and prior published historical reconstructions. We compare the pristine to present-day size spectrum based on published estimates of direct human impacts and population declines (see Materials and Methods). Given the diversity of taxa and observational scales, we tailored our methods to the available data for each group. For example, phytoplankton are estimated globally using satellite images of surface chlorophyll, with algorithms designed to estimate total depth-integrated biomass. Heterotrophic bacteria and all zooplankton groups, from single cells to large crustaceans, are estimated from >220,000 water samples, geographically distributed as shown in Fig. 1A (black points), and interpolated over the whole ocean based on environmental correlates. The biomass of fish, which aggregate, migrate, and can escape capture, is challenging to estimate from point samples but are nonetheless intensively “sampled” by commercial fisheries; so, we use two independent global process models constrained by global catch data (*39*, *40*) (table S5). Last, given that large marine mammals can individually range across whole ocean basins, we compile global population estimates for most marine mammal species (*n* = 82) and use body size allometry and geographic ranges to estimate the remainder (*n =* 44; fig. S9; see Materials and Methods).

Our approach allows us to estimate the biomass of 12 major groups (aggregated into 5 groups in the figures) over approximately 33,000 1° grid points of the global ocean. The total group biomasses are broadly concordant with prior global compilations of particular groups, as shown in tables S1 and S2 (*41*–*43*). We partition each group biomass into order of magnitude body mass size classes over their respective size ranges, based on published size distributions (table S3), or if unknown, we partition biomass uniformly across the group size range and test this assumption with alternative distributions and sensitivity analysis (figs. S11 and S12) (*44*). For each group and size class, we estimate a logarithmic 95% confidence interval (CI), representing a multiplicative fold uncertainty. We outline our data sources, methods for estimating pristine, and present-day biomass, as well as the various sources of uncertainty in Materials and Methods and the Supplementary Materials (*44*), and report here the most robust overall results.

## RESULTS

We find that the reconstructed pristine global ocean size spectrum is largely consistent with Sheldon *et al.*’s original hypothesis (Fig. 1B), particularly in the epipelagic (the upper, sunlit portion of the ocean). The least squares regression fit to log abundance versus log size class is close to the long-hypothesized value of −1 (−1.04, 95% CI: −1.05 to −1.02) and exhibits remarkable regularity. This regularity derives from the fact that we are aggregating organisms over very large spatial extents and size classes and representing the relation over an enormous 23 orders of magnitude, over which even large residual variation is undetectable.

**Fig. 2.**
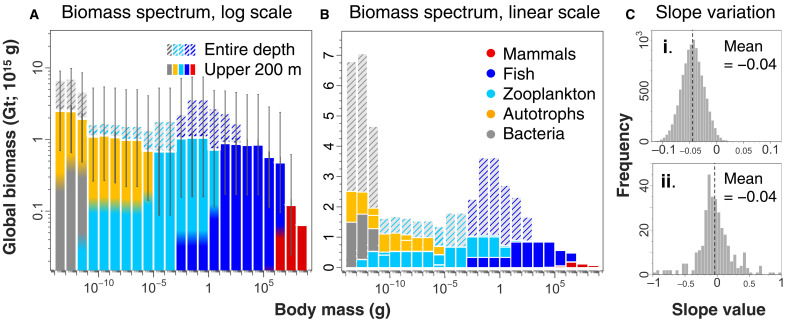
The pristine ocean biomass spectrum. Total estimated historic ocean biomass in each order of magnitude size class is approximately 1 Gt (gigatonnes or petagrams = 10^15^ g), with exceptions at either extreme. Biomass is shown in the upper 200 m of the ocean (colored) and extending to the seafloor (hatched colors represent the group that dominates below the epipelagic; bacteria dominate <10^−11^ g, and mesopelagic fish dominate size classes 10^−3^ to 10^3^ g). (**A**) Global ocean biomass is shown on a logarithmic scale with logarithmic 95% CIs on epipelagic biomass. Bin colors show the relative fraction of each group (no relation to *y* axis). (**B**) Biomass estimates in (A) are shown on a linear scale to highlight differences of bacteria and whales from the overall trend. (**C**) Frequency histograms of biomass spectrum slopes for (i) resampled data incorporating uncertainty in both biomass [shown in (A)] and the size distribution of each group (*n* = 10,000 simulations) and (ii) prior published slope values for *n* = 325 measured biomass spectra (from 47 separate studies; note the difference in *x* axis from C, i).

This pattern indicates that biomass is generally not dominated by any best adapted size, as is evident when abundance is transformed to biomass (Fig. 2A). However, our results show exceptions at the extremes: Bacteria and whales diverge from the uniformity in biomass, which are more obvious when displayed on a linear scale (Fig. 2B). Whereas all other groups sum to approximately 1 gigatonne (Gt) wet weight (10^15^ g) of biomass in each order of magnitude size bin, the size bins dominated by bacteria and whales are notably different. Although there is considerable uncertainty in our estimates (Fig. 2A) (*44*), these differences are more pronounced and significant when we consider the size spectrum over the entire water column (cross-shading in Fig. 2), with bacteria dominating the biomass in the cold, dark ocean. Mesopelagic nekton are also relatively abundant, leading to a peak at 0.01 to 1 g, but these estimates are prone to large uncertainties (*41*, *42*, *44*), and we hesitate to draw a strong conclusion about this portion of the spectrum. Sensitivity analysis shows that the overall size spectrum slope is robust to both the choice of group biomass distributions and the possible broad variations in biomass within our uncertainty bounds (slope 95% CI = ±0.043; Fig. 2C, i, and figs. S11 and S12) (*44*). Slope estimates are also robust to different fitting methods (table S7) and binning schemes (fig. S11). Last, our data allow us to approximate size spectra over most 1° lat-lon regions of the ocean, and although we are less confident in these ~33,000 slope estimates, given the patchiness in the data and lack of some important major groups, our data suggest that size spectra slopes may be similar across global environmental gradients (fig. S13).

**Table 1.** Summary of pristine ocean biomass estimates, data sources, and methods among groups. Estimated pristine ocean biomass (wet weight; 1 Gt =10^15^ g) are compared across major groups for the epipelagic (top 200 m) and full depth to the seafloor. Because depth-resolved estimates of fish and mammals were not available, these taxa were roughly allocated to top and full depth categories. Fold uncertainty is a multiplicative 95% CI on biomass. These biomass estimates are consistent with other global meta-analyses across major groups (*20*, *41*–*43*, *67*) and were used to partition biomass across the body size range to build the global ocean size spectra in Figs. 1 and 2. GLM, generalized linear model; SST, sea surface temperature. 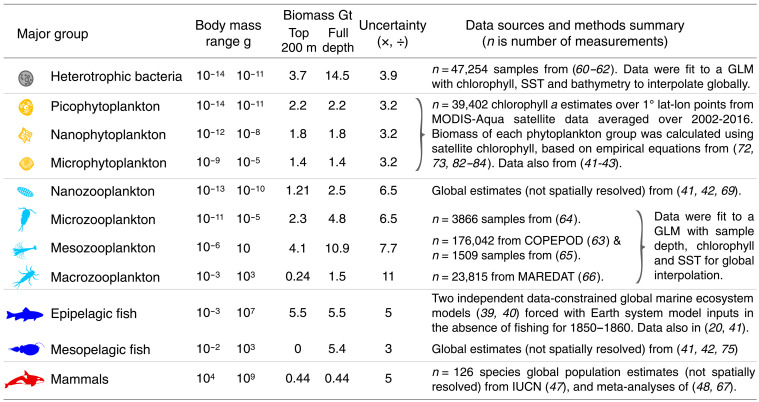

**Fig. 3.**
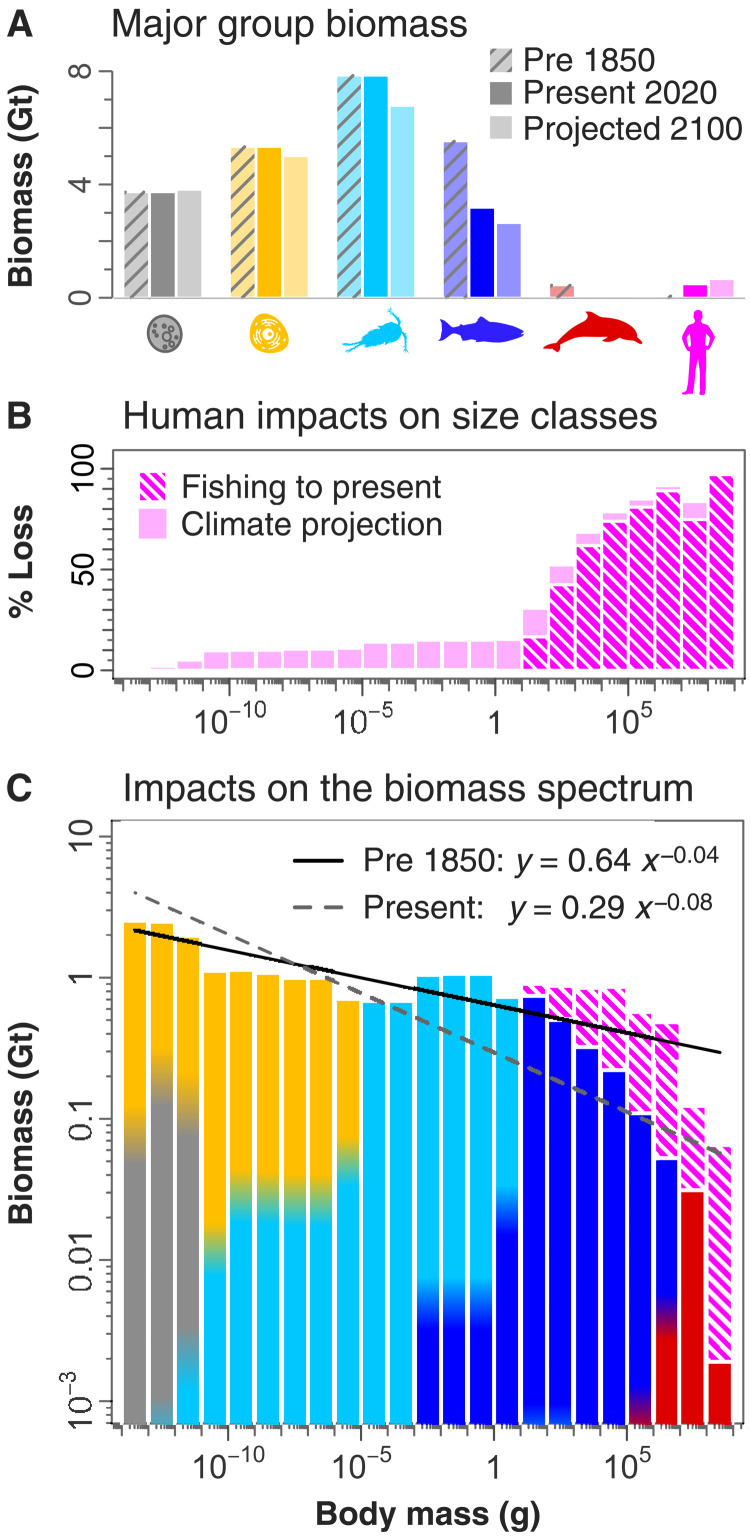
Direct human impacts on ocean biomass. (**A**) Estimated biomass in the ocean’s upper 200 m is compared across major groups at three time periods: pre-1850 (hatched bars), current levels (solid bars), and projected to 2100 (lighter shading). (**B**) Direct human impacts are estimated across size classes and show progressively more extreme impacts above 10 g (we do not consider possible indirect impacts on bacteria and plankton groups). (**C**) These impacts have altered the shape of the biomass spectrum, with the current-day regression (2020; dashed gray line) having a significantly steeper slope than the pre-1850 regression (solid black line; pristine, as in Fig. 2). Hatched pink area is called the lost biomass spectrum.

Our analysis also shows that the whole-ocean pattern is not immune to human impacts. Despite marine mammal and wild fish catches amounting to <3% of annual human food consumption (*44*), the previously reported cumulative impacts of industrial fishing and whaling (*45*–*48*) are notable when viewed within the context of the global size spectrum. Fish >10 g in size and marine mammals are likely to have been reduced in biomass by about 2 Gt (~60% reduction; Fig. 3A), and the largest size classes appear to have experienced a near 90% reduction in biomass since 1800 (Fig. 3B). We also estimate potential climatic impacts that could occur over the next century. To do so, we use published impacts on major groups from high emission–projected changes in climate [representative concentration pathway (RCP) of 8.5; (*49*–*51*)] and assume that current fishing effort remains constant (Fig. 3B). These estimates suggest that fishing and whaling could have already had a considerably greater impact among large size classes than will climate change over the coming decades. Although there are considerable uncertainties in these projections, it is clear that the direct impacts of fishing and whaling have markedly altered the ocean biomass spectrum. We find that the upper one-third of the biomass spectrum has been severely truncated and the whole-spectrum slope significantly altered (Fig. 3C).

## DISCUSSION

Our estimated reconstructions of the pristine ocean biomass suggest a robust law-like property of marine systems that appears to hold across nearly all marine life. These estimates imply that biomass is nearly invariant across logarithmic size classes but diverges at the extremes with a relatively higher abundance of bacteria and lower abundance of whales. These divergences mark a departure from what might be considered a strong interpretation of the Sheldon hypothesis. Moreover, the cumulative impacts of historical fishing and whaling appear to have resulted in major alterations to the present-day size spectrum. We discuss the theoretical and applied implications of these findings.

### Implications for theory

Much of the current size spectrum theory has focused on particular groups such as plankton or fish, typically using body mass allometries for those groups to estimate key variables of the theory. Although several size spectrum theories make predictions for the slope based only on a small number of variables, such as metabolic scaling and predator-prey body mass relations (*16*, *18*, *19*, *21*, *25*), these predictions depend on the scaling exponents of the presumed underlying body mass allometries (*16*–*18*, *21*, *27*–*29*) or require that particular combinations of exponents sum to one for dimensional reasons (*16*, *17*, *25*). Since the size spectrum apparently holds over nearly all eukaryotes, the question may be raised as to whether the body mass allometries, which have been used to explain the size spectrum, show the same consistency over this vast size range. In contrast with what is widely assumed, predator-prey mass ratios are extremely variable, with up to six orders of magnitude residual variation in any given size class (fig. S14) (*52*–*54*), and metabolism does not scale as 3/4 with body size but closer to one (fig. S15) (*33*, *34*, *55*, *56*). Near-proportional metabolic scaling, for example, significantly alters the predictions of several of the theories that rely upon it, yielding predicted slopes that differ significantly from the observed slope by −0.2 (*18*, *28*) to +0.25 (*20*, *21*, *23*), depending on the theory. While this may be insufficient to falsify these theories, it suggests that size spectrum theory may need to be questioned in light of new evidence. More broadly, the size spectrum appears more general and consistent than many of the individual-level processes and allometries presumed to be its cause.

### Human impacts on energy flow

Our results place the global loss of marine animals due to human consumption within the context of the size spectrum. Prior work has pointed out that humans are now the top predator in the marine ecosystem, having extracted most of the predatory fish and mammals that previously occupied the upper ranges of the size spectrum (*57*). This raises the question: Do humans now play the same role previously played by the predators we have removed? Have we simply inserted ourselves into the marine size spectrum and now act as a functionally equivalent top predator? The answer is clearly no.

Although human biomass is now among the largest of any single vertebrate species (at approximately 0.4 Gt), it remains unexpectedly small relative to the marine biomass that has been lost from the largest size classes as a direct result of fishing and whaling (~2.7 Gt; Fig. 3A). Furthermore, we can estimate the metabolic energy associated with the lost biomass spectrum using empirical metabolic scaling relations for fish and mammals. As detailed in Materials and Methods, we estimate that the lost biomass spectrum (pink hatched area in Fig. 3C) would have previously dissipated energy (in units of biomass) amounting to as much as 14 Gt/year (12 Gt/year, if we only consider fish). This represents a lost metabolic demand that is two orders of magnitude greater than the ~0.1 Gt/year of biomass energy obtained through fishing [and not exceeding ~0.2 Gt/year; (*58*)]. Clearly, humans have not merely replaced the ocean’s top predators but have instead, through the cumulative impact of the past two centuries, fundamentally altered the flow of energy through the ecosystem. Further work is necessary to understand how this massive alteration of biomass flow may be affecting ocean ecosystem functioning.

In summary, our results provide evidence that the pristine size spectrum regularities previously observed at the local scale among particular groups are largely preserved at the global scale across all groups, as hypothesized by Sheldon *et al.* (*1*) half a century ago. The fact that biomass is so evenly distributed across such a vast size range raises questions about the generality of existing size spectrum theory, demanding renewed effort to uncover the dominant underlying processes. At the same time, our analysis has quantified a major impact of humanity on the distribution of biomass across size ranges, highlighting the degree to which human activities in the Anthropocene have altered life at the global scale.

## MATERIALS AND METHODS

To construct the global ocean size spectrum and assess human impacts, we drew on a diverse assemblage of data using methods tailored to the data available for different taxonomic groups and following a number of simplifying assumptions. Here, we summarize our data sources and methodology for estimating the biomass of each major functional group (heterotrophic bacteria, phytoplankton, zooplankton, fish, and mammals; Table 1), as well as the assumptions and limitations associated with reconstructing pristine ocean biomass. We describe how these estimates are used to build the global marine size spectrum, by partitioning biomass across relevant size classes for each of 12 major groups. We outline the various sources of uncertainty and how we estimate the 95% CI of our biomass estimates for each functional group. We also detail our approach to estimate human impacts on the size spectrum through animal hunting and climate change. Full details of our approach are in the Supplementary Materials, including all data and referenced sources, as well as source code, allowing our analysis to be reproduced and updated as new data become available (https://doi.org/10.5281/zenodo.5520055).

The size spectrum can be represented in many ways. In Fig. 1B, we show the relation between the logarithm of numerical abundance versus logarithmic size class, obtaining a slope near −1. As we show in Fig. 2, this is equivalent to a slope near 0 for log biomass versus log size class. Alternatively, we can normalize our size classes by dividing by their width, which gives a normalized abundance spectrum with a slope near −2 and a normalized biomass spectrum with a slope near −1 (*2*, *13*). Furthermore, size spectra can be represented as a probability density function (exponent near −2), a complementary cumulative distribution function, or rank-size relation [exponent near −1; i.e., Zipf’s law; (*59*)]. It is also important to recognize that Sheldon *et al.* (*1*) used octave size classes (powers of 2) in terms of body mass but plotted these size classes in terms of equivalent spherical diameter (ESD). If size class is instead based on log ESD, then log abundance versus log ESD gives an expected exponent near −3 (*44*).

### Data sources

Our data sources are summarized in Table 1. All raw data, comprising more than 290,000 samples of abundance or biomass of particular taxonomic groups at various ocean locations and depths, are included in the Supplementary Materials and our source code (https://doi.org/10.5281/zenodo.5520055). In addition, we provide our biomass predictions for each spatially resolved major group for each 1° latitude and longitude region of the ocean (fig. S1), amounting to >33,000 biomass estimates for each of eight spatially resolved groups (Table 1).

Bacteria abundance data were obtained from >47,000 sample measurements deriving from three sources (*60*–*62*), and ~500 in situ measurements of individual cell size were taken from five studies covering pelagic and coastal regions (*44*). Phytoplankton biomass across all groups were derived from sea surface temperature and satellite chlorophyll *a* measurements, obtained from monthly climatologies of MODIS-Aqua (Moderate Resolution Imaging Spectroradiometer aboard the *Aqua* spacecraft, 4-km resolution) from 2002 to 2016, and aggregated to a 1° spatial resolution. Zooplankton biomass across all groups except nanozooplankton derive from a total of >200,000 biomass samples aggregated from four sources, most notably COPEPOD and MAREDAT (*63*–*66*). Fish biomass was estimated from two data-constrained global ecosystem models [BiOeconomic mArine Trophic Size-spectrum model (BOATS) (*39*) and FishErIes Size and functional TYpe model (FEISTY) (*40*)]. Mammal biomass was estimated from 126 species global population estimates deriving from the International Union for Conservation of Nature (IUCN) (*47*) and meta-analyses (*48*, *67*). In addition, for building generalized linear models for bacteria and zooplankton groups, we used bathymetry data for each 1° region of the global ocean from General Bathymetric Chart of the Oceans, as well as annual average chlorophyll *a* and sea surface temperature from MODIS-Aqua.

### Reconstructing pristine biomass

To estimate pristine biomass before industrial-scale fishing and whaling (circa 1850), we consider only the direct impacts of animal capture on the extracted fish and mammal groups. We have not considered the many possible indirect impacts, including trophic cascades, changes in bioenergetic pathways, or habitat loss, and so we do not attempt to estimate possible indirect changes to the various groups of bacteria, plankton, and mesopelagic fish, or nonmesopelagic fish less than 10 g in size, and assume these groups have remained the same through time. This represents an important but unavoidable source of uncertainty.

Reconstructions of pristine fish biomass are also subject to considerable uncertainty. For our estimates of fish (which includes true fish, cephalopods, and benthic invertebrates), we relied on two spatially resolved marine ecosystem models that predict fish biomass from environmental parameters (*39*, *40*). These models were built independently and are each constrained with recent fishery harvest data. For the pristine estimates used here, the models were each run without fishing and forced with preindustrial environmental inputs from an Earth system model (CESM-BGC1) for the decade 1850–1860. The resulting biomass estimates derived from these simulations are necessarily dependent on the assumptions of the two models and the Earth system model used to force them. Despite the inherent uncertainties, both models produce global-scale patterns in catch and biomass consistent with current-day empirical estimates (table S1) (*20*, *41*, *42*) and yield pristine biomass estimates within a factor of two of one another (table S5).

To estimate pristine marine mammal biomass, we used current global population estimates, adding estimates of population declines, obtained from the IUCN (*47*) or else took the upper CI values obtained from multiple present-day global abundance estimates, when there were only qualitative reports of declines. For many of the largest whales, we relied on pristine reconstructions from (*48*). In the absence of any data on pristine estimates or population declines, we assumed that pristine and present-day global populations are the same (*n* = 47 of 126 species; see data S1).

### Estimating major group biomass

Below, we summarize our methods for estimating biomass for each major group. A full description is available in the Supplementary Materials.

#### Heterotrophic bacteria and zooplankton

Our data sources for heterotrophic bacteria abundance did not distinguish between bacteria and archaea, so we include both of these groups in our heterotrophic bacteria biomass estimate. These are often just referred to as bacteria; photosynthetic bacteria are included in phytoplankton. Bacterial abundance was multiplied by mean bacteria cell size to estimate global biomass. We did not find a statistically significant difference in mean bacteria cell size between coastal and open ocean samples, so we assumed a single mean individual bacteria cell size across the global ocean (fig. S4) (*44*). To estimate total zooplankton biomass, we split zooplankton into four groups defined by their linear size ranges: nano (0.8 to 5 μm), micro (5 to 200 μm), meso (200 μm to 2 cm), and macro (0.2 to 10 cm) (*43*, *68*). Nano- and microzooplankton cover protists groups such as heterotrophic flagellates, dinoflagellates, ciliates, and juvenile mesozooplankton (*64*, *69*). Mesozooplankton covers groups such as copepods, larvaceans, amphipods, and giant rhizaria (*65*, *70*). Macrozooplankton includes groups such as chaetognaths, euphausiids, tunicates, fish larvae, ctenophores, and cnidaria (*71*).

Global estimates of abundance for bacteria and biomass for each zooplankton group (micro-, meso-, and macrozooplankton, as well as all other animals that pass through a zooplankton life stage, but excluding nanozooplankton) were calculated using generalized linear models fit to log-transformed sample measurements from across the world’s oceans (*n* = 226,405; Fig. 1A). Bathymetry, satellite sea surface temperature, and chlorophyll *a* measurements were used as environmental predictor variables. For nanozooplankton, we were unable to find a sample source with which to generate a global statistical model, so we used an aggregate estimate of biomass for this group and partitioned biomass across its size range (*42*). Since no uncertainty bound was provided for nanozooplankton estimates, we use the uncertainty range calculated for microzooplankton, which overlaps taxonomically with the nanozooplankton. For bacteria and zooplankton groups, we follow the approach of (*41*, *42*) in reporting our uncertainty interval, which derives from (i) the standard error from 1000 bootstrap predictions of mean global abundance or biomass from the statistical model and (ii) the SD of the log-transformed sample data on which the statistical model was fit (*44*).

#### Phytoplankton

To estimate global phytoplankton biomass, we used annual average satellite chlorophyll *a* observations from MODIS-Aqua, from 2002 to 2016, and empirical equations of chlorophyll *a* with depth and by functional type, to calculate the global biomass of pico-, nano-, and microphytoplankton. Our estimate of total phytoplankton biomass includes both autotrophs and mixotrophs, since both of these groups contain chlorophyll *a* and so are represented in satellite estimates. To convert satellite chlorophyll *a* observations to phytoplankton biomass, we first split total satellite surface chlorophyll *a* in each 1° region into pico-, nano-, and microphytoplankton chlorophyll *a* using equations from (*72*). These equations model the nonlinear shifts in the relative proportions of each group—in regions of low chlorophyll *a*, picophytoplankton tend to dominate the biomass, and in regions of high chlorophyll *a*, the larger nano- and microphytoplankton tend to dominate. We converted surface chlorophyll *a* into total chlorophyll in the water column in each 1° region using an empirical equation in (*72*) and, finally, converted total chlorophyll *a* into carbon and, last, wet biomass following (*73*). We obtained the cumulative 95% CI by multiplying the reported multiplicative 95% CIs from each empirical equation (*44*).

#### Fish

The major group we have called “fish” include epipelagic, mesopelagic, and demersal organisms as well as some benthic organisms that are not included in zooplankton data. Hence, our fish group includes true fish (bony and cartilaginous) and invertebrates of the same size range (mostly cephalopods). To calculate total fish biomass, we estimate nonmesopelagics and mesopelagics separately.

We estimate biomass of epipelagic and demersal fish, cephalopods, and large benthic invertebrates using spatially resolved estimates derived from two process-based global marine ecosystem models (*39*, *40*). These models were built independently, are each comprehensive in their spatial coverage and size range, and both constrained with fishery catch data. The first estimate comes from the BOATS (*39*), a size-based global model that represents harvested organisms (including epipelagic and demersal fish, squid, and benthic invertebrates) from 10 g to 100 kg, using average temperature in the top 75 m with integrated primary production in the water column as environmental inputs. The second estimate comes from the FEISTY (*40*), which represents the global biomass of epipelagic and demersal fish and benthic invertebrates from 1 mg to 125 kg. This model estimates biomass of functional types, with sea surface temperature, zooplankton carbon concentration, and particulate organic carbon flux to the sea floor as environmental inputs.

We used publicly available output from these models to derive our global biomass estimates from simulations where each model is forced with environmental inputs from the CESM-BGC1 Earth system model (both models’ output available at https://www.isimip.org/about/marine-ecosystems-fisheries/). For each model, we calculated their global average biomass using the decadal average from 1850 to 1860 from simulations of pristine ocean biomass with no fishing. We chose this decade because it was the furthest in the past that both models have been run through, and it is far enough back in time that anthropogenic climate impacts are not discernible from existing climate variability (*74*). To obtain the biomass of fish with body sizes greater than 100 kg, we used the scaling relationship between biomass and body size from (*20*) to define the relationship between biomass and body size as a power function with an average exponent of −0.06 (assuming an average ecosystem trophic transfer efficiency of 0.125 and an average predator-prey mass ratio of 1000). The fish component of this biomass was then calculated by subtracting mammal biomass, which was estimated separately (see “Mammals” section). To calculate the uncertainty range for our estimates of epipelagic and demersal fish biomass, we follow the approach of (*42*), taking the 90% CI of global fish biomass from (*20*) as representative of this uncertainty range.

Mesopelagic fish live in the mesopelagic zone (200 to 1000 m) of the global ocean. Although difficult to sample directly, these fish can be detected by the reflectance of acoustic signals from sonar, and this reflectance can be used to estimate their biomass (*75*). For our estimate of mesopelagic fish biomass, we used results from (*75*), who developed a model to estimate global estimates of mesopelagic fish biomass from acoustic surveys, accounting for uncertainties in the body structures of mesopelagic fish (*44*).

#### Mammals

Marine mammal biomass was estimated from species-specific surveys for the majority of all known marine mammal species. Population data of varying quality exist for more than two-thirds of all approximately 126 extant species of marine mammals, with nearly all current estimates of marine mammals taken from the IUCN (*47*) (*n* = 87). We estimated “prewhaling” population counts by taking larger valued abundance estimates or upper CIs when extensive capture levels have been reported or else from expert opinion or modeling studies (*n* = 83). For most species of whales, we relied on the historic reconstruction estimates in (*48*). When no additional information was available beyond current estimates (*n* = 47 species), we assumed pristine numbers were the same as exist currently. We believe this approach is more likely to underestimate pristine abundance than overestimate it in most instances, but the values provided are among the most complete tallies of currently available data for marine mammal biomass.

Estimates of marine mammal uncertainty is obtained from additional species population data from multiple additional sources, summarized in the mammal dataset—available in the Supplementary Materials, which includes minimum, mean, and maximum values for 115 marine mammal species—and drawn from 275 primary published sources (*67*). Given the necessarily limited knowledge of the prewhaling period, we assumed the uncertainty range for pristine mammal biomass to be slightly more than double the range of our calculated present-day mammal biomass [from 2.2 to 5; (*44*)].

### Sources of uncertainty

There are several kinds of uncertainty associated with our reconstructions of the pristine size spectrum and its present-day structure. Below, we list the kinds of uncertainties and how we attempted to deal with each.

#### Boundaries of the “Sheldon hypothesis”

There are conceptual uncertainties in delineating the physical and taxonomic boundaries of what should be included to test the conjecture raised by Sheldon *et al.* (*1*). In particular, the question arises of whether to include all ocean depths, estuaries, benthic and sediment species, seabirds, etc. Sheldon *et al.* made measurements of mostly the pelagic environment in the upper water column, which characterizes most empirical studies of size spectra up to the present (summarized in table S3) (*5*). We have thus tried as closely as possible to reproduce this analysis globally, over all taxa, but our estimates of fish biomass also include demersal fish and large benthic organisms over the continental shelves. We have also excluded brackish waters and mangrove ecosystems, as well as species such as seabirds that do not physically reside in the ocean. Although we have thus focused on the epipelagic, we show results that include biomass estimates down to the seafloor in Fig. 2 and Table 1.

#### Measurement error

There are various kinds of measurement errors associated with the estimation of each major group. For example, phytoplankton estimates from satellite imagery of surface chlorophyll *a* concentrations require well-calibrated ground truthing and reproducible relationships between chlorophyll across each phytoplankton group (*76*). Zooplankton biomass samples have been taken with dozens of different gear types and mesh sizes but cannot capture very small or large zooplankton as reliably (*63*), and it is difficult to evaluate the effects of zooplankton avoidance, aggregation, and diurnal migration (*77*). Fish biomass is particularly difficult to estimate over the global ocean, and although our marine ecosystem models are constrained by global catch data, there are large uncertainties in harvest data due to underreported and illegal catches (*58*). Last, mammal biomass derives from visual observation at the sea surface and in breeding grounds, with unequal observational effort across regions and species (*47*). We did not explicitly attempt to deal with measurement error and assumed that our data sources are the best available, and the specific studies from which they derive had attempted to deal with these measurement issues. More generally, we do not expect these errors to significantly bias our final analysis, which aggregates a great number of measurements into relatively coarse order of magnitude size classes (*44*).

#### Geographic bias

With the exception of phytoplankton, which derives from satellite data with uniform global coverage and is averaged over more than a decade, all other groups are not uniformly sampled over the global ocean or at different depths. As shown in Fig. 1A, sampled observations of heterotrophic bacteria and the various zooplankton groups are patchy, with far fewer samples in the southern hemisphere. We attempted to correct this geographic bias by using generalized linear models that interpolate biomass over the global ocean using environmental predictor variables (chlorophyll *a*, sea surface temperature, and bathymetry), which are consistent in their coverage globally (*44*).

#### Major group biomass

For each major group, we estimated global biomass uncertainty, represented as a multiplicative 95% CI around the average biomass estimate (Fig. 2A). Following the approach of (*41*, *42*), we report these uncertainty ranges as multiplicative fold changes (×/÷) from the mean, rather than additive changes (±). We elected to use a multiplicative factor to represent uncertainty because the distribution of sample data is best approximated by a log-normal distribution, and the geometric mean (i.e., the arithmetic mean on log scale) will give an estimator that is more robust to outliers, particularly when data are sparse (*41*, *42*). Given the range of source methods we use to estimate biomass, our calculations of 95% CI were not the same for all groups, such that uncertainty bounds are not strictly comparable across different methodologies. Nonetheless, our mean biomass and uncertainty ranges for each group are consistent with those from other global biomass studies that use different methodologies (*20*, *41*–*43*, *67*) (table S1). Full methods to calculate uncertainties for each group are given in the Supplementary Materials.

#### Major group size distributions

While most size distributions within plankton groups are well studied and similar [i.e., approximately even biomass across log size classes; table S3; (*5*)], relatively little is known of the overall size distributions of heterotrophic bacteria, fish, and mammals. For mammals, we used average species body mass with each global population count, thus avoiding the need for any assumptions of their size distributions. For groups where less is known about the size distribution, we used an even biomass across log size classes as a starting point and tested this assumption by sensitivity analysis with randomly generated size distributions (see “Building the size spectrum” section). We find that the particular size distribution within major groups has very little effect on the overall global size distribution (Fig. 2C, i, and fig. S12).

### Building the size spectrum

To construct the abundance and biomass spectra in Figs. 1 and 2, we partitioned the global biomass estimates for each major group across their relevant size classes (Table 1). Biomass was assigned to relevant group size classes in different ways, depending on the group, as described above. It is important to note that given the nature of the data, macrozooplankton, for example, may include fish larvae, and mesozooplankton may contain juvenile species of what are otherwise macrozooplankton. For mammals, on the other hand, we used global population estimates for each species and associated these numbers to a mean species body mass. We do not expect individual versus species mean values of body size to bias our results over order of magnitude size classes.

Most research over the past 50 years has revealed that the biomass distribution within major groups is approximately invariant across log size class (Fig. 2C, ii, shows the distribution of slopes over 325 size spectra from 47 different studies; see also table S3). We thus assumed an even distribution of biomass across log size classes in each group and then tested the sensitivity of this assumption to different size distributions. We randomly allocated group biomass across log size classes where total group biomass itself is also drawn at random from a log-normal distribution centered on the mean biomass with SD obtained from the 95% CI fold uncertainty for 10,000 simulations. Our sensitivity analysis shows that even quite extreme variation in group size distribution still yield overall biomass spectrum slopes very near our reported value of −0.04, with little variation, as shown in Fig. 2C, i (see also fig. S12). For the top 200 m, we obtained a normal distribution of slope values with mean of −0.049 and SD of 0.021, thus giving a 95% CI for the slope of the average global biomass of −0.085 to −0.003. Across the entire water column, we obtain a normal distribution with mean of −0.056 and SD of 0.020, giving a 95% CI for the slope of −0.095 to −0.017 (fig. S12).

Although we report best-fit parameters from ordinary least squares (*2*), we also investigated the effect of alternative fitting methods including reduced major axis (RMA) (*78*) and maximum likelihood (MLE) (*79*), finding very slight differences between fitting methods, with biomass spectrum slopes that ranged from −0.03 (MLE) to −0.04 (RMA), as summarized in table S7. We also investigated the use of alternative binning of biomass, such as half order of magnitude, and found this also had very little effect on the overall slope (fig. S11).

### Fishing and climate projections

To estimate the impacts of fishing on the size spectrum, we use two estimates (*45*, *46*), focusing on the depletion caused by industrial fishing up until the early 2000s. We combined these two estimates for the fraction of fish >10 g remaining from pristine fish biomass [58% inferred from (*46*) and 33% inferred from (*45*)] by taking their geometric mean, giving a value of 44% of fish >10 g remaining. We therefore calculated the size spectrum slope that would be consistent with the 44% depletion among the fraction >10 g, assuming that fish ≤10 g have not been significantly affected by current harvesting in the global average. This assumes that the slope has thus steepened by −0.17 for all fish >10 g (*44*).

In addition to steepening in the size spectrum due to fishing, there have also been significant declines in large mammals, particularly whales. We compiled both prewhaling and current abundance for more than two-thirds of all ~126 included species of marine mammals. These data are derived from over 275 primary sources and drawn from meta-analyses of (*48*, *67*), as well as expert opinion and compilation from (*47*). These data suggest that among the smallest marine mammals (10 to 100 kg), only 47% remain, but progressively smaller fractions characterize the larger size classes, with possibly only 25% of mammals remaining in the 10- to 100–metric ton (MT) size class and only 3% of blue whales, making up the largest size class (*47*, *48*). Combining the global estimates of fish reductions (*45*, *46*) with those of marine mammals (*47*, *48*, *67*), we calculated the percentage lost in each size class (Fig. 3B) and the resulting shape of the global biomass spectrum (Fig. 3C) (*44*).

We compare fishing and whaling impacts up to the present with combined projected future fishing and climate change impacts across the entire size spectrum. To assess combined fishing and climate impacts to the end of the 21st century, we assume that the impacts of fishing and climate on biomass are additive, with little nonlinear interaction between them (*50*). We also assume that the cumulative historical fishing impacts are maintained into the future, such that effective fishing effort remains approximately constant. Projected climate change impacts are obtained from Earth system model simulations that estimate the changes in ocean temperature, circulation patterns, and biogeochemical cycling that will result from a given future trajectory of atmospheric greenhouse gases. Here, we use the worst-case scenario trajectory, RCP of 8.5. Rather than relying on a single model, we take the mean estimate from the 10 climate models that participated in the fifth simulation round of the climate model intercomparison project (CMIP5), as analyzed by (*80*).

Estimated changes in phytoplankton, zooplankton, and fish biomass were calculated using ecosystem modules that were run in a fully coupled mode with the ocean modules (*49*–*51*), and we assumed uniform changes across each group’s respective size ranges. Unlike the case for the preceding groups, there are no global process models available for marine mammals. Instead, we use the model of (*51*), combined with the mean projected sea surface temperature increase from the 10 CMIP5 climate models. Last, we were unable to find prior estimates for the relative changes in bacteria concentrations under climate change. Instead, we used an approach analogous to that used by (*51*) for marine mammals and estimated changes in global bacteria biomass using the temperature and chlorophyll *a* dependence from the statistical model we developed to estimate global bacteria abundance, forced by sea surface temperature and surface chlorophyll *a* concentrations for 2090 to 2100 from CMIP5 Earth system models (*44*).

### Energy loss from the reduced biomass spectrum

We estimate the loss in energy use associated with the lost portion of the biomass spectrum (pink hatched area in Fig. 3C). To do so, we make use of resting *W*_rest_ and maximal *W*_max_ metabolic rates of fish using data from (*56*) (data are shown in fig. S15). These data were converted to watts and temperature corrected to 15°C using reported *Q*_10_ values in (*56*). The resulting metabolic scaling relations with body mass (g) are as follows

$$W_{\text{rest}}=2.71\times 10^{-4}m^{1.02}(R^{2}=0.90;n=112\;\text{fish species};m_{\text{min}}=0.5\;g;m_{\text{max}}=7500\;g)$$

$$W_{\text{max}}=1.21\times 10^{-3}m^{1.02}(R^{2}=0.94;n=79\;\text{fish species},m_{\text{min}}=0.5\;g;m_{\text{max}}=8500\;g)$$

We assume that resting metabolism represents a lower bound and that actual energy use in the wild is within these extremes. Given that exponents in both relations are 1.02, we can take the geometric mean of their coefficients as a possible estimate of active energy use (5.7 × 10^−4^). We convert watts into grams per year, following (*81*), who estimated energy content of biomass over many aquatic species to be approximately 4250 J/g. Hence, we convert basal metabolism (in watts ≡ joules per second) into a basal energy use in units of grams per year. Further, we convert this relation into a mass-specific metabolic rate by dividing by body mass, such that mass-specific metabolism is expressed in terms of grams of embodied energy respired per gram tissue (1/year). The relation of estimated active mass-specific energy use *w*_active_ in units of 1/year is

$$w_{\text{active}}=4.25\;m^{0.02}$$

For each log size class >10 g, we multiply the reduced biomass (pink hatched area, Fig. 3C) by the respective *w*_active_ calculated for the geometric mid-point of the size class. The sum over all such size classes is equal to 14.3 Gt/year and represents the total metabolism (Gt/year) associated with the reduced biomass spectrum (pink hatched area, Fig. 3C). Doing the same calculation for resting mass-specific metabolism gives a total reduced metabolism of 6.7 Gt/year. Excluding mammals and considering only fish, the lost active metabolic energy is estimated to be 12.1 Gt/year.

## Supplementary Materials

### This PDF file includes:

Tables S1 to S7 Figs. S1 to S15 Overview Human impacts on the biomass spectrum Statistical considerations Energetic considerations Data and methods References
