# Supplementary material for: The global ocean size spectrum from bacteria to whales
Source: Sci Adv. 2021 Nov 10;7(46):eabh3732. doi: 10.1126/sciadv.abh3732 (PMC8580314; doi:10.1126/sciadv.abh3732)
Supplement: Supplementary file 1 — Tables S1 to S7 Figs. S1 to S15 Overview Human impacts on the biomass spectrum Statistical considerations Energetic considerations Data and methods References [file sciadv.abh3732_sm.pdf]

Science Advances
AAAS

Supplementary Materials for

The global ocean size spectrum from bacteria to whales

Ian A. Hatton*, Ryan F. Heneghan, Yinon M. Bar-On, Eric D. Galbraith

*Corresponding author. Email: i.a.hatton@gmail.com

Published 10 November 2021, *Sci. Adv.* **7**, eabh3732 (2021)
DOI: 10.1126/sciadv.abh3732

The PDF file includes:

Tables S1 to S7
Figs. S1 to S15
Overview
Human impacts on the biomass spectrum
Statistical considerations
Energetic considerations
Data and methods
References

Other Supplementary Material for this manuscript includes the following:

Data S1

Contents

Overview
- Uncertainty

Data and methods
- Bacteria
- Phytoplankton
- Zooplankton
- Fish
- Mammals

Human impacts on the biomass spectrum
- Fishing impacts
- Climate projection impacts

Statistical considerations
- Fitting size-frequency distributions
- Sensitivity analysis of group size distributions
- Slope dependence on group biomass

Energetic considerations

References

Overview

To construct the global ocean size-spectrum, we drew on a diverse assemblage of complementary data, methods and assumptions. Here, we outline our data and methods for each group and our procedures used to reach the estimates of size-specific abundance, biomass and uncertainty values reported in the paper. We also detail the approach used to estimate how humans have altered the size-spectrum through animal hunting and climate change. Finally, we outline our statistical approach, including more general considerations of our treatment of uncertainty and fitting methods. In addition, we provide all data and referenced sources, such that our analysis can be reproduced and updated as new data becomes available: https://doi.org/10.5281/zenodo.5520055.

Table S1. General summary of ocean biomass. Body mass range and total ocean biomass (wet weight; Gt ≡ 10^{15} g) are summarized across major taxonomic groups. Fold uncertainty is a multiplicative factor of the mean (×/÷) that expresses our confidence in the data akin to a 95% confidence interval (see text). These estimates are compared to values in the literature, particularly Bar-On et al. 2019 (*20*, *41–43*, *67*).

Major group	Body mass range (g)		Biomass (Gt)	Fold (×/÷) uncertainty	Bar-On (*41*) biomass (Gt)	Fold (×/÷) uncertainty	Other biomass estimate (Gt)	
Bacteria (to seafloor)	10^{-14}	10^{-11}	**14.48**	3.85	**13**	3	**10**	Buitenhuis 2013 (*43*)
Phytoplankton	10^{-14}	10^{-5}	**5.34**	3.28	**4**	6.3	**6.3**	Buitenhuis 2013 (*43*)
Zooplankton	10^{-13}	10^{3}	**19.71**	7.4	**17.5**	10	**19**	Buitenhuis 2013 (*43*)
Fish	10^{-3}	10^{7}	**10.9**	5	**12**	7	**14**	Jennings 2015 (*20*)
Mammals (current)	10^{4}	10^{9}	**0.068**	5	**0.04**	1.4	**0.065**	Kaschner 2004 (*67*)

There are several ways that a global size-spectrum analysis could be undertaken, from the coarse use of published global biomass estimates aggregated by major group and size class, to a very disaggregated assembly of existing observational data, which could include many tens of thousands of species-specific surveys and mass-specific size-spectra for different taxa and regions. The coarsely-aggregated extreme is shown in Table 1. In contrast, a highly disaggregated approach would require a vast effort, and would be plagued by difficulties regarding spatial and temporal bias. For this paper we elected to focus on a middle path between the two extremes, making use of many kinds of pre-existing data, while employing a number of procedures of intermediate sophistication. Our methods are tailored to the data existing for each major taxonomic group, and are designed to maximize the utility of ancillary information.

Our methods include statistical and process models constrained by large existing surveys or satellite imagery, ensembles of dynamical models developed by independent research groups, and species specific survey data over their global geographic range. Our resulting global biomass estimates are all within a factor of two of previously published global biomass estimates (*20*, *41–43*, *67*) (Table S1). Abundance and biomass estimates for more resolved taxonomic groupings are shown in Table S2 and the geographic distribution of our biomass estimates for each major group are shown in Fig. S1. Finally, greater resolution and summary tables are available as part of the output of the code linked to in the first paragraph.

In order to study the size-spectrum over the entire ocean, we necessarily employed a number of assumptions to delimit the scope of our study, on which our results are strongly dependent. One major assumption is what constitutes "ocean" life. Like Sheldon and the many size-spectra studies that came after, we take oceanic life to refer largely to the pelagic environment (organisms living suspended in the salt water column of the ocean)(*1*, *5*, *85*). We therefore did not consider benthic organisms explicitly or in great depth, though aggregate measures of organisms that live on the seafloor are included in estimates of "non-mesopelagic fish" from the FishErIes Size and functional Type model (FEISTY; (*40*)) and the BiOeconomic mArine Trophic Size-spectrum model (BOATS; (*39*)). We also elected not to include brackish waters, mangroves or seabirds. The exclusion of benthic and sediment organisms has the largest impact on bacteria biomass estimates, given their extremely large estimated biomass in sediments (*86*).

Table S2. Summary of ocean biomass among major groups. Ocean abundance and biomass (wet weight) are compared across major groups at a more resolved level than Table S1 (see Table S1 caption), and include values for the top 200 meters and extended to the sea floor. Mammals and fish are estimated at historic levels**.**

Major group	Body mass range (g)		Global ocean (to seafloor)		Top 200 meters		Fold (×/÷) uncertainty
			Abundance	Biomass (Gt)	Abundance	Biomass (Gt)	
Bacteria	10^{-14}	10^{-11}	2.1×10^{29}	**14.48**	5.5×10^{28}	**3.73**	3.85
Picophytoplankton	10^{-14}	10^{-11}	3.5×10^{28}	**2.19**	3.5×10^{28}	**2.19**	3.22
Nanophytoplankton	10^{-12}	10^{-8}	9.7×10^{25}	**1.8**	9.7×10^{25}	**1.8**	3.22
Microphytoplankton	10^{-9}	10^{-5}	7.3×10^{22}	**1.35**	7.3×10^{22}	**1.35**	3.22
Nanozooplankton	10^{-13}	10^{-10}	2.1×10^{27}	**2.5**	1×10^{27}	**1.21**	6.53
Microzooplankton	10^{-11}	10^{-5}	8.9×10^{24}	**4.79**	4.3×10^{24}	**2.33**	6.53
Mesozooplankton	10^{-6}	10	3.6×10^{20}	**10.88**	1.3×10^{20}	**4.07**	7.7
Macrozooplankton	10^{-3}	10^{3}	3.7×10^{16}	**1.54**	5.8×10^{15}	**0.24**	11.39
Epipelagic fish	10^{-3}	10^{7}	1.2×10^{17}	**5.53**	1.2×10^{17}	**5.53**	5
Mesopelagic fish	10^{-2}	10^{3}	4.1×10^{16}	**5.37**	0	**0**	3
Mammals (historic)	10^{4}	10^{9}	4.4×10^{8}	**0.44**	4.4×10^{8}	**0.44**	5

We also did not explicitly consider several taxonomic groups including the following (global biomass estimates are from (*41*, *42*): viruses (~0.3 Gt); fungi (~2.6 Gt); seagrasses (~2 Gt), and corals (~0.5 Gt). Our methods and estimates include groups such as molluscs including squid (0.5 Gt) and nematodes (0.1 Gt) that we did not consider separately. We do not distinguish heterotrophic bacteria from archaea (3 Gt), and refer to these two groups as "bacteria". Note that cyanobacteria and other photosynthetic or mixotrophic bacteria are included in phytoplankton.

We show estimates of biomass over the top 200 meters of the ocean as well as the entire water column down to the sea floor to provide perspective on the vertical distribution of marine life. The top 200 meters (epipelagic) is where the majority of life's diversity resides and where all major groups commonly exist, as well as where data have the best coverage. Extending our analysis from the top 200 meters to the ocean depths has the greatest effect on estimates of bacteria biomass (Table S2).

Size-spectra have been sampled at various locations and represented in many ways. Individual size may be in units of length, volume, wet or dry mass, and counted over an area or volume, which may be transformed into biomass or energy density. Individuals may be binned into any number of logarithmic or linear size-classes, or represented as a probability or cumulative density function, and fit using various methods such as least squares or maximum likelihood (*2*, *5*, *12*). These varied published representations can obscure commonalities among observed patterns, which, when expressed as a log size vs. log frequency distribution, tend to be strikingly similar with exponents near a value of −1, as revealed by a recent meta-analysis (*5*), and shown in Table S3 and Fig. S2.

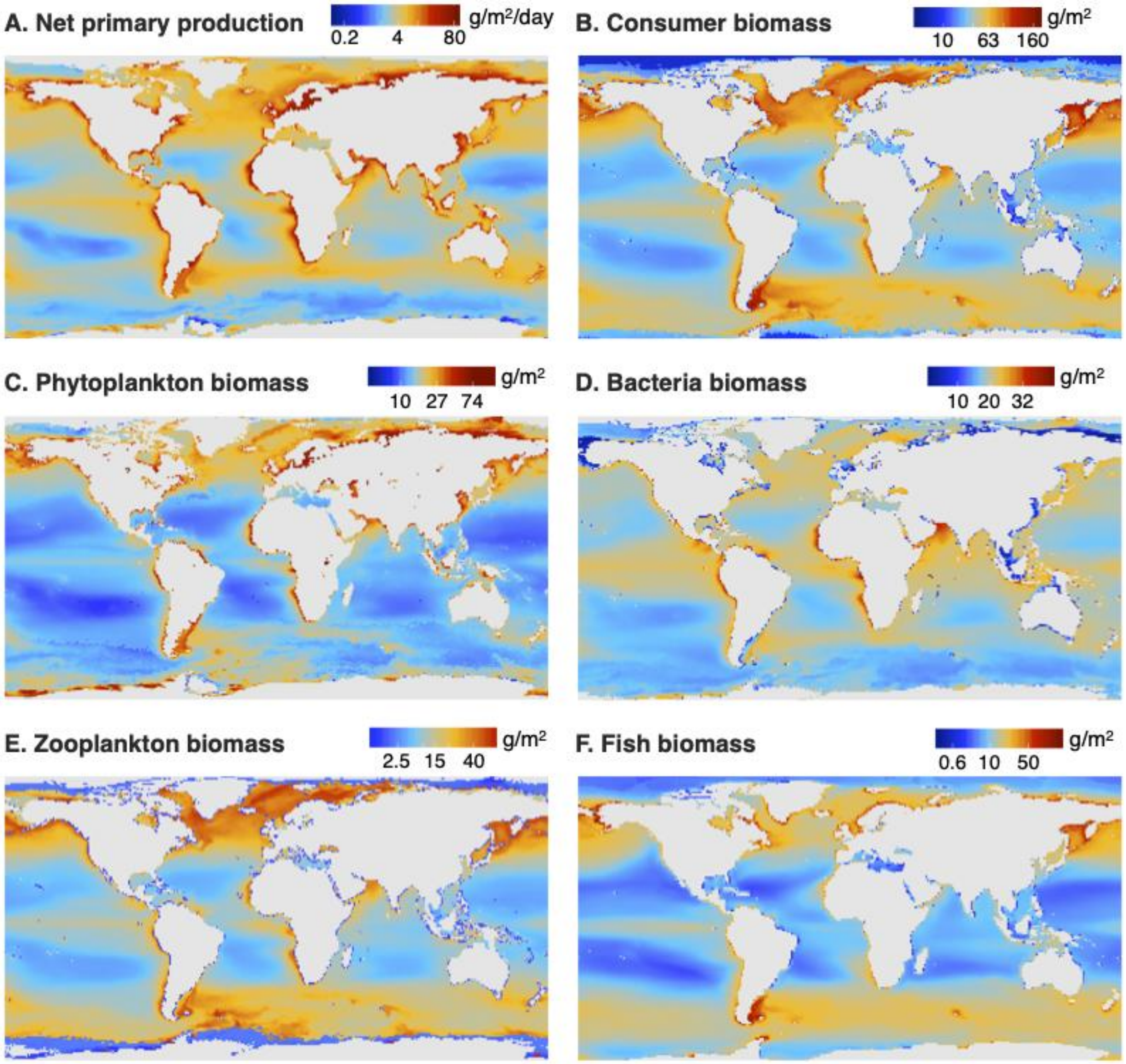

Fig. S1. Geographic distribution of biomass by major taxonomic group. All biomass is for the top 200 m of the global ocean in g/m^2. **A.** Net primary production (NPP) on which the vast majority of all other biomass is based. **B**. All consumer biomass is the sum of biomass in D to F that depends on the flux in A. **C.** Phytoplankton biomass is the standing stock that is largely responsible for NPP in A. **D-F.** Biomass of (D) bacteria, (E) zooplankton and (F) fish. Note the color scale of each major group is not the same, and was instead chosen to better distinguish the geographic distribution of biomass within a group, rather than facilitate cross-group comparison.

Another consideration with respect to the Sheldon hypothesis is that the "pristine" biomass distribution, which existed prior to human hunting and habitat modification, differs from the current biomass distribution. On the one hand, the interest in the Sheldon hypothesis as a possible universal law-like property of intact aquatic systems would prompt use of the reconstructed pristine biomass, which we have done here. On the other hand, it is of significant interest to know how human impacts might have altered the size-spectrum through a long history of harvesting, as

well as the current expectations of how humans may impact the size-spectrum through climate change (*35*). We therefore also provide an estimate of how historical impacts from fishing and whaling have shaped the current size-spectrum, as well as projecting changes in biomass under a pessimistic scenario (RCP8.5) of future changes in climate from year 2000 to 2100 (Fig. 3).

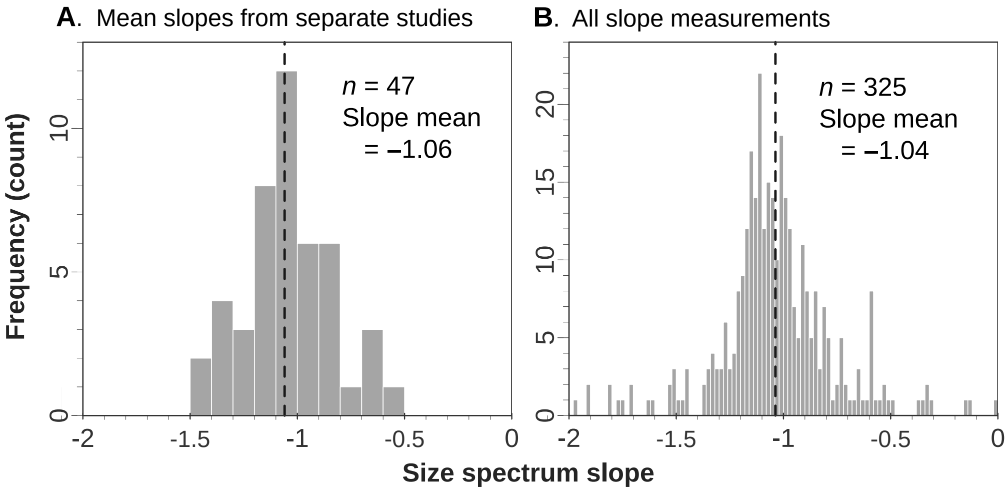

Fig. S2. Summary of size spectra slopes from the literature. These slopes represent the normalized biomass spectra slopes, or equivalently, the log of the number of individuals in each logarithmic size class. **A.** A histogram of the mean slope from each of 47 separate studies. **B.** A histogram of all 325 slopes reported across these 47 studies. Data are from the meta-analysis in (*5*), supplemented with 43 additional size spectra from (*7*, *8*, *87–90*). A summary of this meta-analysis is reproduced in the Supplementary Data file, with some of the more important studies listed in Table S3.

A number of assumptions must be made in order to aggregate individuals into body mass size-classes. We chose the major groups of bacteria, phytoplankton, zooplankton, fish and mammals, as these capture common distinctions between functional groups, but more importantly, they demarcate important differences in our methods for estimating biomass, as described further in the section "Data and methods". Among some major groups, further sub-divisions were necessary for improving our estimates given the data available (listed in Table S2). Finally, these sub-divided groups were split into order-of-magnitude size classes. While admittedly coarse, this represents a common level of resolution that can be applied to all major groups with similar confidence. For many groups, the available data allows much more refined size-class binning, but we instead set the bin size to a coarse order of magnitude to avoid making additional assumptions for less resolved group size distribution. This provided us with 23 separate and relatively well estimated data points on which to construct a regression, which is discussed further in "Statistical considerations".

Table S3. Summary of some important size spectra studies. The number of locations (*n*) may refer to separate regions or distinct time periods (with maximum depth shown in meters; Sheldon 1972 included a few size spectra greater than 500 m, but most were <200m). The reported smallest to largest groups and order of magnitude range of body sizes (grams fresh mass) is approximated. The mean slope of the size-spectrum (normalized biomass) is reported with the range of slope values underneath, where available. Further studies are listed in (*5*, *91*).

Locations	Groups included	Size range (g)		Mean slope	Reference
Various ocean regions, (500 m), $n \approx 80$	Bacteria to mesoplankton	10^{-13}	0.1	**–1**	Sheldon et al. 1972 (*1*)
Northwest Atlantic, (400 m), $n = 9$	Bacteria, nano to mesoplankton	10^{-14}	0.1	**–0.99**<br>–1.01 to –0.96	Quinones et al. 2003 (*3*)
North Pacific Central Gyre, (120 m), $n = 24$	Picoflagellates to macrozooplankton	10^{-9}	10^{-2}	**–1.16**<br>–1.34 to –0.95	Rodriguez & Mullin 1986 (*4*)
Various ocean & lakes, (200 m), $n = 7$	Phytoplankton to fish	10^{-12}	10^{2}	**–1.04**<br>–1.04 to –1.07	Boudreau & Dickie 1992 (*7*)
Antarctic Peninsula, (200 m), $n = 27$	Nanoplankton to macrozooplankton	10^{-12}	10^{-1}	**–1.05**<br>–1.2 to –0.96	Witek & Krajewska-Soltys 1986 (*92*)
Antarctic Peninsula (400 m), $n = 22$	Bacteria to zooplankton	10^{-13}	10	**–0.88**<br>–1.02 to –0.79	Garcia-Munoz et al. 2014 (*93*)
SW Atlantic Shelves (50 m), $n = 10$	Picoplankton to mesozooplankton	10^{-14}	10^{-2}	**–1.12**<br>–1.29 to –0.99	San Martin et al. 2006 (*6*)
North Aegean Sea (100 m), $n = 15$	Microzooplank. to macrozooplankton	10^{-7}	10^{-1}	**–1.12**<br>–1.27 to –1.0	Frangoulis et al. 2017 (*94*)
Eastern Brazil Coast (80 m), $n = 4$	Mesozooplank. to macrozooplankton	10^{-6}	10^{-1}	**–0.94**<br>–1.01 to –0.86	Marcolin et al. 2013 (*91*)
Northwest Mediterranean (70 m), $n = 2$	Plankton (unspecified)	10^{-11}	10^{-6}	**–1.07**<br>–1.2 to –0.95	Vidondo et al. 1997 (*89*)
Western English Channel (50 m), $n = 12$	Bacteria to macrozooplankton	10^{-8}	10^{2}	**–1.11**<br>–1.14 to –1.05	Atkinson et al. 2020 (*5*)
Scotia Sea (1000 m), $n = 3$	Microplankton to macrozooplankton	10^{-10}	10^{2}	**–1.08**<br>–1.09 to –1.06	Tarling et al. 2012 (*95*)
Lake Constance, (20 m), $n = 11$	Bacteria to crustaceans	10^{-13}	10^{-3}	**–1.00**<br>–1.16 to –0.94	Gaedke 1992 (*9*)
Various lakes, (25 m), $n = 18$	Phytoplankton to fish	10^{-11}	10^{3}	**–1.0**<br>–1.14 to –0.86	Cyr & Peters 1996 (*11*)
Lake Ontario & Malawi, (250 m), $n = 2$	Phytoplankton to fish	10^{-12}	10^{4}	**–1.03**<br>–1.04 to –1.02	Sprules 2008 (*96*)
Lake Ontario & Michigan (250 m), $n = 2$	Phytoplankton to prey fish	10^{-12}	10^{4}	**–1.04**	Sprules & Goyke 2008 (*10*)
Lakes in Argentina, (3 m), $n = 3$	Pico to macroplankton	10^{-14}	10^{-4}	**–1.2**	Cózar et al. 2003 (*90*)
Lake Superior, (120 m), $n = 2$	Phytoplankton to prey fish	10^{-11}	10^{3}	**–1.11**	Yurista et al. 2014 (*8*)

Uncertainty

Uncertainties in our estimates come from a range of sources. Below we provide more detail to our description of sources of uncertainty in Materials and Methods.

Boundaries of the Sheldon hypothesis

There is uncertainty regarding the physical and taxonomic boundaries of what ought to delimit the "Sheldon hypothesis" in the ocean. Sheldon et al. first hypothesized that a constant biomass across log size classes extended from bacteria to whales, with little indication of the physical boundaries or the many taxonomic groups, in which this pattern should be relevant (*1*). For example, should we assume that the physical boundary in which the pattern is most relevant is only the open surface waters of the epipelagic, or might it extend to the mesopelagic or all the way to the seafloor and seafloor sediment? Likewise, are we to assume that all taxonomic groups whose niche overlaps with these environments should be included in the Sheldon hypothesis, or are we to include only species that physically reside in these environments, excluding say, seabirds and polar bears? While the inclusion/exclusion of minor taxonomic groups should have little bearing on the overall Sheldon spectrum, the physical boundary, and particularly the depth to which we delimit the problem alters the shape of the distribution, as shown in Fig. 2. In order to facilitate comparison with Sheldon et al's original study, and the many that have come after (Table S3), we have focused largely on the epipelagic, though our marine ecosystem models that were used to estimate fish biomass include some benthic and demersal groups along the continental shelves. Nonetheless, we show results for two domains in the ocean: the upper 200m and the full water column (Fig. 2, Table 1 and Table S2). In addition, we have excluded most species that do not physically reside in the water column, and have excluded brackish waters, estuaries and mangroves.

Measurement error

Estimation of phytoplankton biomass depends on satellite estimates of chlorophyll a concentration at the surface, while estimates of bacteria and zooplankton are based on interpolations from hundreds of thousands of sample estimates. These estimates depend on well calibrated observing instruments. Small drifts in instrument calibration can cause significant errors in reported chlorophyll concentration, and abundance measures, which in turn drive errors in estimates of group biomass (*76*). Zooplankton biomass samples have been taken with dozens of different gear types and mesh sizes over the past 60 years (*63*). However, many of these gear and mesh types are not suitable to capture small or large zooplankton due to passive or active avoidance, aggregation and diurnal migration, leading to (usually) underestimates of total zooplankton biomass (*77*). Further, the ability of many mesopelagic fish to avoid nets can produce taxon-specific biases that are difficult to estimate (*97*). Because of the inherent difficulties with scientific sampling, some fish estimates make use of fishery data to help constrain biomasses. Industrial fishing activities capture a large fraction of global fish production, providing independent constraints on coupled fish-fishery models (*39*), though they also have large uncertainties due to unreported, underreported and illegal catches (*58*). Finally, in the case of mammals, most abundance estimates come from visual observation at the sea surface or in breeding grounds. In addition to the measurement errors associated with different methods, there is also uncertainty in how comparable these different methodologies are (*43*). This is particularly relevant for attempting to compare estimates of uncertainty intervals between different groups.

Sampling bias

The ocean covers 2/3 of the planet, and large parts of it have not been sampled thoroughly. What is more, the environment is continually fluctuating, which can cause large temporal variability at any point in the ocean. As a result, there is the potential for large biases in the spatial and temporal distribution of observational samples (*98*) which is likely to greatly exceed the measurement error. We address this bias using statistical and/or process-based models that capture how biomass abundance varies with the environment, and then resample the models at the global scale using realistic average environmental conditions. Although the models have large uncertainties for predicting the biomass under any given set of environmental conditions, they have much less uncertainty associated with the global mean values, which are the target of interest here.

Major group size distributions

Much research over the past 50 years has revealed that, within limited taxonomic domains, the size-frequency distribution is characteristically the same as the broader size-spectrum that we show in Fig. 1 (power law distribution with an exponent near -1; see Table S3). For some major groups, however, very little is known of the size distribution, which is particularly true for bacteria, for which we are aware of only a single study that explicitly studied their size distribution in the open ocean (*99*). We therefore test this uncertainty by using alternative size distributions and undertaking a sensitivity analysis of our assumption of an equal distribution of biomass across order of magnitude size bins for each major group, described below in the section "Group size distributions and sensitivity analysis".

Calculating uncertainty bounds by major group

For each major group, uncertainties are represented as 95% confidence intervals of the average biomass estimate (Fig. 2 A). Following the approach of (*41*), we report these uncertainty ranges as multiplicative fold changes (×/÷) from the mean, rather than additive changes (±). This is done because the distribution of sample data is best approximated by a log-normal, and the geometric mean (i.e. the average on log scale) will give an estimator that is more robust to outliers, particularly when data are sparse. Multiplicative uncertainty is more robust to large fluctuations and appropriate for log-normally distributed data. Moreover, much of the error in estimation, as well as the natural fluctuations in time or in space or among taxa, are multiplicative rather than additive. Given the range of sources we use to calculate our biomass estimates, the method we used to estimate uncertainty was not the same for all groups. The extent to which uncertainty bounds across methodologies can be compared remains difficult to evaluate. We summarize the method used to estimate uncertainty intervals for each major group below.

Table S4. Summary of estimation methods by major groups and key gaps in knowledge.

Major group	Groups included	Estimation method	Key knowledge gaps
Bacteria	Bacteria & archaea	Georeferenced samples (*n* = 47254) & statistical model	Sampling bias and size distribution of group
Phytoplankton	Pico, nano & microphytoplankton	Satellite chlorophyll a globally & empirical models	Chlorophyll a to biomass conversion
Zooplankton	Nano, micro, meso, & macrozooplankton	Georeferenced samples (*n* = 205,190) & statistical model	Sampling bias due to varying gear types
Fish	Epipelagic, demersal & mesopelagic	FishMIP (two independent global fisheries models)	Estimates are sparse, fish < 1 g poorly known
Mammal	All marine mammal species (*n* = 127)	Species specific global surveys and geographic range maps	Historical abundance and human influence

Bacteria and zooplankton uncertainty. Global estimates of abundance for bacteria and biomass for all zooplankton groups (nano-, micro-, meso-, and macro-zooplankton, as well as all other animals that pass through a zooplankton life-stage) were calculated using generalized linear models fit to log-transformed sample measurements. For our statistical models of bacteria and zooplankton we follow the approach of (*41*, *42*) in reporting our uncertainties using the geometric mean of two measures of uncertainty: 1) the standard error from 1000 bootstrap predictions of mean global abundance or biomass from the statistical model, and 2) the standard deviation of the log-transformed sample data on which the statistical model was fit. The geometric mean of the two uncertainty measures is then multiplied by 1.96 to obtain the 95% confidence interval for the mean estimate in log-space. We use the geometric mean of these two uncertainty measures. For the first uncertainty estimate, the standard error of the global mean biomass estimate will be smaller the better the predictive capability of our statistical model. However, this uncertainty does not consider the presence of bias or measurement error in the sample data used to fit the statistical model, therefore this uncertainty estimate is most likely an underestimate. On the other hand, the standard deviation of the log-transformed data would be an overestimate of the uncertainty of average biomass, given it does not take into account the decrease in uncertainty that comes from the averaging that takes place when the statistical model is fit.

For bacteria, an additional step is required where mean global abundance is multiplied by average bacteria cell size to estimate mean global biomass. We incorporate this added uncertainty in our global bacteria biomass estimate by multiplying 95% confidence interval of geometric mean bacteria cell size with the 95% confidence interval of the geometric mean global abundance of bacteria.

Phytoplankton uncertainty. Global phytoplankton biomass was calculated from satellite surface chlorophyll observations. We used an empirical equation from (*72*) to derive total chlorophyll in the water column from satellite surface estimates, and then another empirical equation from (*73*) to convert total chlorophyll into phytoplankton carbon and finally wet biomass. These empirical equations were published with reported 95% confidence intervals in log-space, and we obtained

the cumulative 95% confidence interval by multiplicatively combining the reported 95% confidence intervals from these two empirical equations.

Fish uncertainty. We split 'fish' (which includes large cephalopds and invertebrates) into two groups; mesopelagics and non-mesopelagics. For mesopelagic fish, we quantified the uncertainty of our estimate using results from (*75*), who assessed how uncertainty in the composition of the mesopelagic community causes uncertainty in estimates derived from bioacoustics surveys. For non-mesopelagic fish, including cephalopods and benthic invertebrates, we obtained estimates of global biomass from two process-based marine ecosystem models; the FishErIes Size and functional Type model (FEISTY; (*40*)) and the BiOeconomic mArine Trophic Size-spectrum model (BOATS; (*39*)). We used publicly available output from these models to derive our global biomass estimates (both models' output available at https://www.isimip.org). To calculate the uncertainty range for our estimates of non-mesopelagic fish biomass, we follow the approach of (*42*), who took the 90% confidence interval of global fish biomass from (*20*) as representative of the uncertainty of their non mesopelagic fish biomass estimate.

Mammal uncertainty. Estimates of marine mammal uncertainty is obtained from additional species population data from multiple additional sources. These include current mammal abundance estimates listed on IUCN species pages, as well as data drawn from Appendix 4 of (*67*), which lists minimum, mean and maximum values for 115 of 126 marine mammal species. The vast majority of these estimates tend to be within a multiplicative factor of three of our current values. Obtaining uncertainty estimates for pre-whaling abundance is more challenging, but are seldomly more than a factor of ten different from other estimates, and on average are a factor of 4.3 of maximum current values from (*67*). Pooling all estimates including those of IUCN (*47*) and current values of (*67*) (which may not be independent of one another) gives a mean fold uncertainty of 2.2 (following methods described in (*41*, *42*)). Given that our knowledge of the pre-whaling period is necessarily vague, we considered a fold uncertainty value of 5 to be cautious, and include all additional abundance estimates used for estimating uncertainty in the Supplementary Data file.

Further information about uncertainty are described under major group headings in the section "Data and methods" below.

Data and methods

In this section, we describe our data sources, methods and models for estimating global ocean abundance and biomass, as well as procedures for estimating uncertainty in these values.

Bacteria

There were two steps to obtain our estimate of global bacteria biomass. First, we fit a statistical model of bacteria abundance with monthly georeferenced chlorophyll a, sea-surface temperature and 1° bathymetry data to interpolate global bacteria abundance. These environmental variables were taken from monthly climatologies of MODIS-Aqua (Moderate Resolution Imaging Spectroradiometer aboard the Aqua spacecraft; (*100*)) 4 km measurements, from 2002-2016. Similarly, 1° bathymetry data was used to calculate the depth-integrated biomass of bacteria in each 1° region of the global ocean, with data drawn from GEBCO (*101*). We then used *in situ* samples of individual bacteria cell size from coastal and open ocean sites to estimate the average individual bacteria cell size, as well as the size range of bacteria. We then multiplied our global bacteria abundance estimate by our estimate of average bacteria cell size to obtain global bacteria biomass. Our data sources for bacteria abundance did not distinguish between bacteria and archaea, so we include both of these groups in our bacteria biomass estimate.

Data sources of bacteria abundance

1. 5671 observations from Wigington et al. 2016 (*60*) https://doi.org/10.1038/nmicrobiol.2015.24
2. 39767 observations from Buitenhuis et al. 2012 (*61*) https://doi.pangaea.de/10.1594/PANGAEA.779142
3. 1816 observations from Lara et al. 2017 (*62*) http://doi.org/10.1126/sciadv.1602565

Statistical model of bacteria abundance

We fit a generalised linear model, with $\log_{10}$ bacteria abundance as the response variable, with a linear relationship for the environmental predictors $\log_{10}$ chlorophyll, temperature, and a third order polynomial for $\log_{10}$ sample depth (Figure 1). All predictors are significant, and the model has an R^2 of 74%.

Total global bacteria abundance

For each grid square, we use temperature, bathymetry and chlorophyll to calculate the depth-integrated density of bacteria (count/m^2). We then summed all grid squares to obtain a total global abundance of $\mathbf{1.41 \times 10^{29}}$ bacteria (×/÷) 3.4-fold 95% confidence interval). This number is close to Bar-On et al. 2019 (*42*) estimate of 1.2×10^{29}, which was derived from three previous studies of total bacteria abundance (*102*): 8×10^{28}; (*103*): 1.7×10^{29}; (*61*): 1.2×10^{29}).

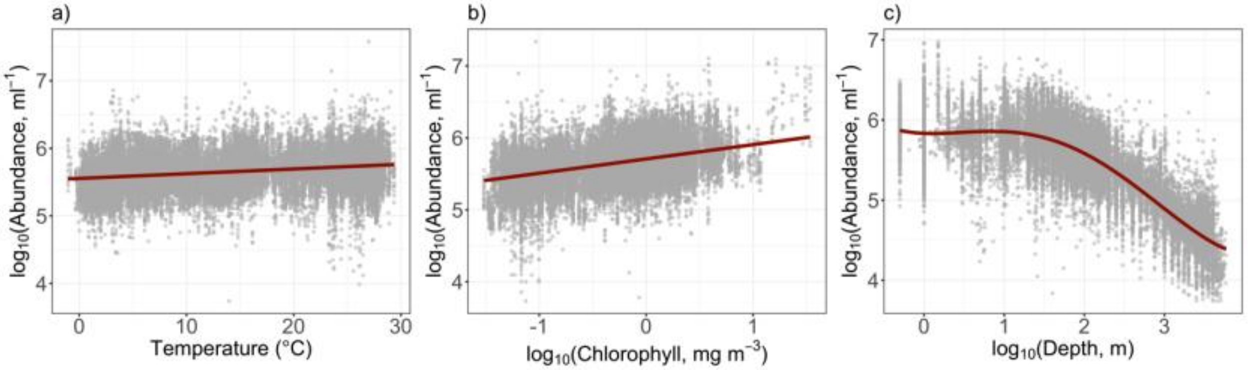

Fig. S3. Model fit to each of the predictor variables for bacteria abundance, with partial residuals.

Mean individual bacteria wet weight

We obtained individual bacteria cell size observations (both carbon and wet weight) from five studies where *in situ* samples were taken from pelagic and coastal regions (*104–108*). For the two largest studies ((*104*): *n* = 145, Sargasso Sea) and ((*105*): *n* = 201, coastal Norway and Denmark), individual bacteria were measured with an electron microscope, after microplankton cells containing chlorophyll were identified and removed. Carbon content was determined using X-ray microanalysis, calibrated on particles with known chemical composition. The cell size distributions (in wet weight) for these studies are similar to distributions from other studies (*99*, *109*) that used flow cytometry—considered a reference technique in bacteria community analysis (*110*)—to count and measure individual bacteria. Contrary to (*111*), we didn't find a statistically significant difference between the distribution of open ocean – Sargasso Sea – observations (95% CI for mean = 7.2-11.6 fg C), and combined coastal observations (95% CI for mean = 9.1-11.4 fg C), so we combined both coastal and open ocean data together, and assumed that individual bacteria cell sizes across the global ocean can be well approximated by a single lognormal distribution.

For each sample used to build the distribution of individual bacteria size in carbon, we also obtained wet weight (biovolume). Bacteria wet weight observations also follow a lognormal distribution (Fig. S4) with a mean individual bacteria wet weight of 0.109 pg (95% confidence interval – 0.096, 0.124 pg; ×/÷ 0.14-fold uncertainty).

Global bacteria biomass and size range

Using our mean wet weight bacteria cell size of 0.109 pg, we calculate global bacteria biomass to be **14.48 Pg** (cumulative 95% confidence interval is ×/÷ 3.85-fold). We used the 99% confidence interval of our bacteria cell size distribution (Fig. S4) to define the size range of bacteria for this study to be 10^{-2} – $10^{0.5}$ pg (10^{-14} – $10^{11.5}$ g).

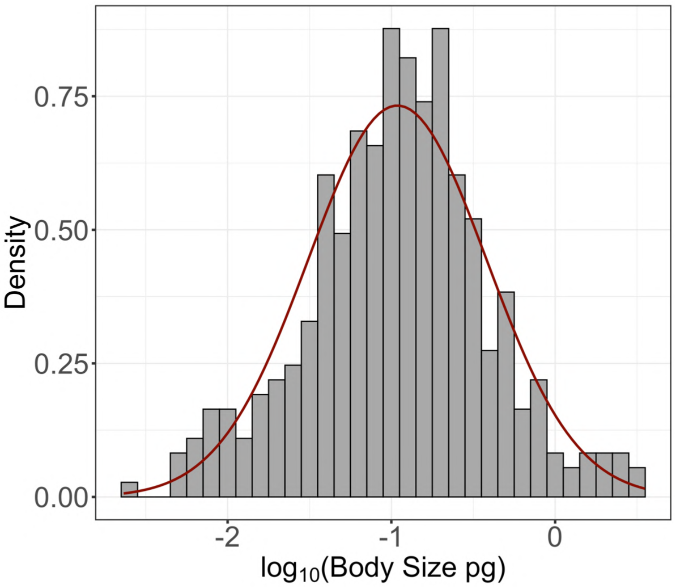

Fig. S4. Histogram of bacterial cell sizes in $\log_{10}$ pg wet weight.

Phytoplankton

To estimate global phytoplankton biomass, we used satellite chlorophyll observations from MODIS-Aqua (*100*), averaged over 2002-2016, and empirical equations of chlorophyll with depth and by functional type, to calculate the global biomass of pico, nano and microphytoplankton. Our estimate of total phytoplankton biomass includes both autotrophs and mixotrophs, since both of these groups contain chlorophyll and so are represented in satellite estimates.

Chlorophyll a from pico, nano and microphytoplankton

First, we calculate the percentage of surface chlorophyll from pico (< 2μm), nano (2-20μm) and microphytoplankton (20-200μm) with the empirical model developed by (*82*). The model was developed with a dataset of phytoplankton pigments from the Atlantic Ocean, and shows close agreement with other global models of phytoplankton functional types developed with datasets for different regions (Figure 4.3 of IOCCG Report, (*112*)). We assume the percentage of chlorophyll from each group is the same as the surface throughout the entire water column. This assumption is supported by Figure 4.3 in the IOCCG (*112*), which shows that the contribution of different phytoplankton groups from the chlorophyll a across the entire euphotic zone (*72*) is in close agreement with the contribution from the Brewin model (*82*), which used only surface chlorophyll a.

Chlorophyll a in euphotic zone

To calculate total chlorophyll a in the euphotic zone from surface chlorophyll a, we use the empirical equations in (*72*). Uitz et al., (*72*) gives two equations for total chlorophyll a from surface chlorophyll a, one for stratified waters (defined as water where euphotic zone depth is less than the mixed layer depth) and another for mixed waters (defined as water where euphotic zone depth is greater than mixed layer depth). To apply Uitz's equations, we divide the global ocean into

mixed and stratified grid squares, using average euphotic zone depth for 2002-2019 from Modis-AQUA, derived with the Lee algorithm (*83*), and annual average mixed layer depth derived from Argo profiles (*84*).

The Uitz equations (*72*) are fit to a sample set from 2419 stations across the global ocean, with ~1800 from stratified waters and ~600 from mixed waters. The fit of the Uitz equations to the data is close to that of earlier work from (*113*), and was chosen for its use of an extensive dataset to derive total chlorophyll a in the euphotic zone from satellite output. In terms of uncertainty, there was a 1.4-fold 95% prediction interval in the relationship between surface chlorophyll a and total chlorophyll a in the euphotic zone (*72*).

Conversion to wet biomass by size class
To convert total chlorophyll a concentration to carbon, we used the empirical equation from (*73*), who fit a relationship between chlorophyll and phytoplankton biomass (carbon concentration), with surface observations from coastal and open ocean stations across the global ocean. The linear relationship between log chlorophyll and log carbon concentration in (*73*), had an R^2 value of 0.9 and a 2.3-fold 95% prediction interval. In each grid square, to calculate total carbon content in the water column, we divided total chlorophyll from step 2 by euphotic zone depth and then used this average chlorophyll concentration to calculate the average carbon concentration with the Marañón equation (*73*). We then calculated average total carbon content in the water column in each grid square by multiplying average carbon concentration by the euphotic zone depth. We then used the percentages calculated in step 1 to break total carbon into pico, nano and microphytoplankton groups. Finally, to convert total carbon of each group into wet weight, we assumed a wet weight to carbon ratio of 10:1 (*7*).

Individual cell size ranges converted to wet weight
The size ranges for pico, nano and microphytoplankton are typically expressed in um ESD: 0.2 μm – 2 μm for picophytoplankton, 2 μm – 20 μm for nanophytoplankton and 20 μm – 200 μm for microphytoplankton (*82*). We convert these cell size ranges to wet weight, assuming 1 g wet weight is equal to 1cm^3.

We calculated global picophytoplankton biomass to be 2.15 Pg, nanophytoplankton biomass 1.8 Pg and microphytoplankton biomass to be 1.35 Pg. Altogether, total phytoplankton biomass **is 5.3 Pg** (cumulative 95% confidence interval is ×/÷ 3.2).

Zooplankton

To interpolate sample estimates of zooplankton biomass, we used satellite chlorophyll a, bathymetry and sea-surface temperature as environmental predictor variables. Monthly chlorophyll a and sea-surface temperature measurements were appended to observations, based on the month in which they were taken, as well as their latitude and longitude. These environmental variables were taken from monthly climatologies of MODIS-Aqua (*100*) 4km measurements, from 2002-2016. Similarly, 1° bathymetry data from GEBCO (*101*) was used to calculate the depth-integrated biomass of zooplankton in each 1° region of the global ocean.

Zooplankton can be broken into four size ranges: nano (0.8µm - 5µm), micro (5µm - 200µm), meso (200µm-2cm), macro (0.2-10cm) (*43*, *68*). Nano and microzooplankton cover protists groups such as heterotrophic flagellates, dinoflagellates, ciliates and juvenile mesozooplankton (*64*, *69*). Mesozooplankton cover groups such as copepods, larvaceans, amphipods and giant rhizaria (*65*, *70*). Macrozooplankton include groups such as chaetognaths, euphausiids, tunicates, fish larvae, ctenophores and cnidaria (*71*).

Nanozooplankton

For this group, we were unable to find reliable resources with which to generate a global statistical model, so we used the estimate of (*42*) of **2.5 Pg** wet weight biomass (assuming a 10-1 wet weight to carbon conversion) for this group. No uncertainty bound was provided for this estimate, as it is derived from only one source (*69*), so we use the ×/÷ 6.5-fold 95% confidence interval calculated for microzooplankton (see below), which overlaps taxonomically with the nanozooplankton (*64*, *69*).

Microzooplankton

Data derived from 4579 observations from Buitenhuis et al. 2010 (*64*).
https://doi.org/10.1029/2009GB003601

After applying quality control to the data (e.g., filtering NA observations for biomass, depth sampled) we were left with 3866 observations. For the 46 observations where depths are greater than 1000m, depth was set to 1000m.

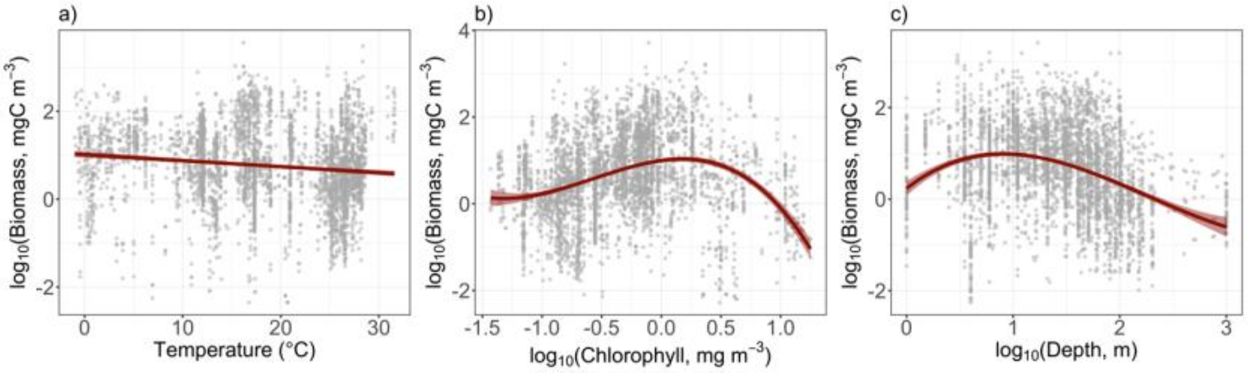

Fig. S5. Model fit to each of the predictor variables for microzooplankton, with partial residuals.

We used a generalised linear model, with a linear relationship between $\log_{10}$ zooplankton biomass and temperature, a third order polynomial for $\log_{10}$ sample depth and another third order polynomial for $\log_{10}$ chlorophyll. All predictors are significant, and the model has an R^2 of 27%. Although the R^2 is low, the relationship of log biomass with environmental variables is reasonable (Fig. S5). For instance, the chlorophyll relationship is not uniformly increasing, but is highest in mesotrophic waters, which matches the results of the model in (*64*).

For each grid square, we use temperature, bathymetry and chlorophyll, and a carbon to wet weight conversion factor of 10 (*7*) to calculate the depth-integrated wet weight biomass of microzooplankton (g m^3) over the water column in each grid square, to obtain a total global biomass of **4.79 Pg** (95% confidence interval for this estimate is ×/÷ 6.5-fold).

Mesozooplankton
Mesozooplankton are not only comprised of groups such as meroplanktonic larvae and copepods, but also giant protists from the group Rhizaria. A recent study (*65*) indicated that although giant Rhizaria are a major fraction of the mesozooplankton, their delicate nature means that they are heavily undersampled in conventional sampling techniques such as the ones used to build the COPEPOD database. To address this, we obtained an estimate of global giant rhizaria biomass separately (see next sub-section) to ensure that this group was properly incorporated into our overall estimate of mesozooplankton biomass. Here we describe all other mesozooplankton biomass estimates.

Data derive from 214,922 observations from the COPEPOD database (*63*):
https://www.st.nmfs.noaa.gov/copepod/atlas/html/taxatlas_b400.html.

After applying quality control to the data (e.g., filtering NA observations for biomass, volume filtered, depth sampled, removing samples from mesh sizes less than 200µm and greater than 2000µm) we were left with 176,000 observations.

We used a generalized linear model, with a linear relationship between $\log_{10}$ zooplankton biomass, and $\log_{10}$ chlorophyll, temperature, and a third order polynomial for $\log_{10}$ sample depth. Observations across measurement types were not converted to carbon, so we also included a factor variable for the measurement type (Fig. S6). All predictors are significant, and we get an R^2 of 83% for this model. The majority of this variance explained comes from the measurement type factor, and when that factor is excluded the R^2 is 18%. The relationships with the environmental variables and sample depth is reasonable, as is the measurement type factor – with wet weight biomass having a higher mean value than the other methods. The spread of the residuals is very wide, about two orders of magnitude around the predicted values (Fig. S6).

Giant rhizaria
Data derive from 1509 observations from the supplement to Biard et al. 2016 (*65*).
https://doi.pangaea.de/10.1594/PANGAEA.858156

Each observation gives the total integrated carbon biomass mg C m^{-2} over a transect of the water column, with 681 observations covering 0-200m, and the remaining 828 observations covering 0-500m. We fit a statistical model to the 0-200m and 0-500m datasets separately, using the 0-500m dataset to estimate total giant rhizaria biomass over the water column (following the approach of (*42*)). Although cell abundance for protists is much lower across the deep ocean (*114*), the lack of sampling below 500m means that total rhizaria biomass may be underestimated.

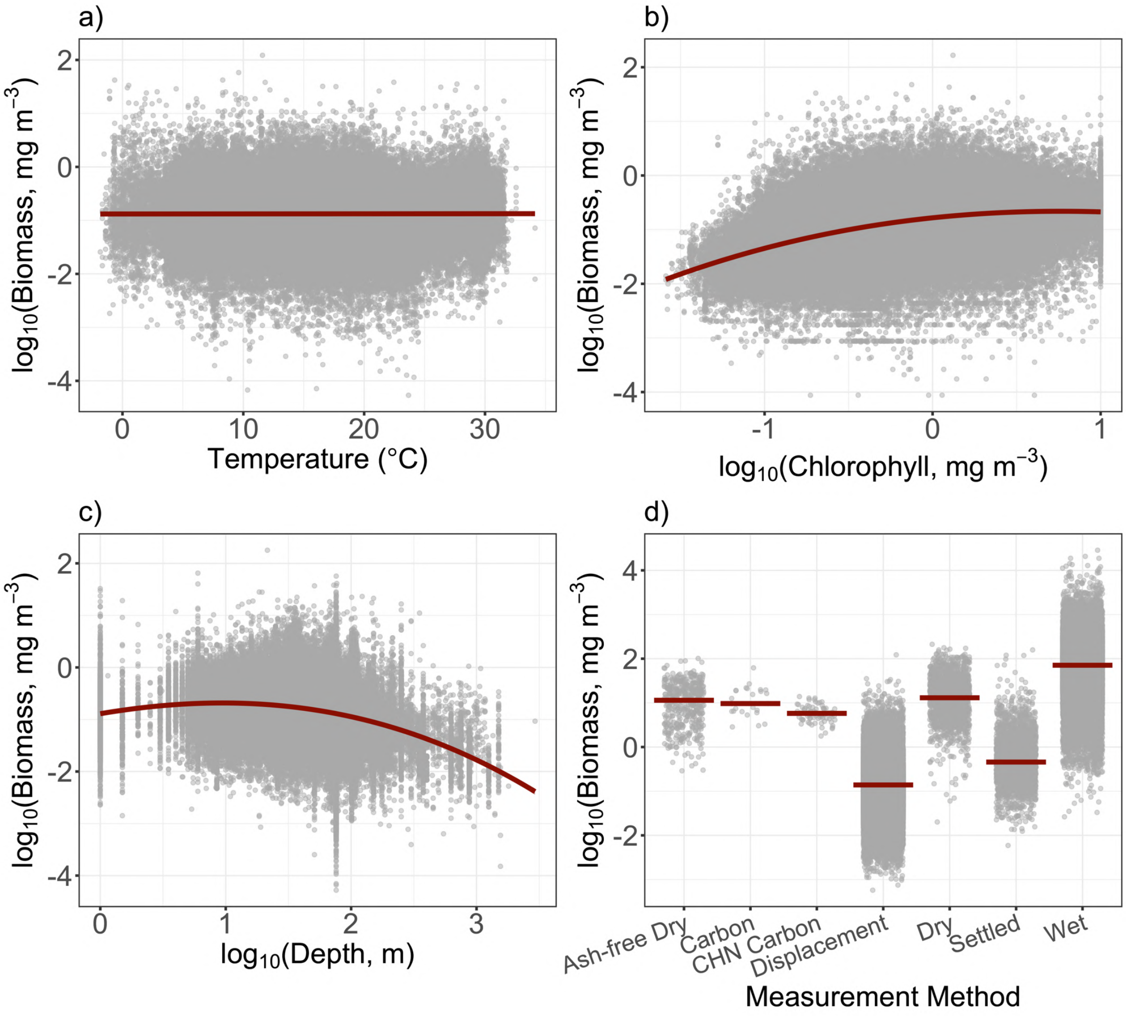

Fig. S6. Model fit to each of the predictor variables for mesozooplankton, with partial residuals.

For our estimate of giant rhizaria biomass over the total water column, and from 0-200m, we used separate generalized linear models, each with a second order polynomial relationship between $\log_{10}$ rhizaria biomass (from 0-500m or 0-200m), and $\log_{10}$ chlorophyll and temperature (Fig. S7). Both temperature and $\log_{10}$ chlorophyll predictors are significant for both models, and we get an R^2 of 25.9% for the model fitted to 0-200 m rhizaria biomass samples, and an R^2 of 22.7% for the model fitted to 0-500 m samples. The fitted relationships between $\log_{10}$ chlorophyll, temperature and $\log_{10}$ biomass were similar across both models (Fig. S7).

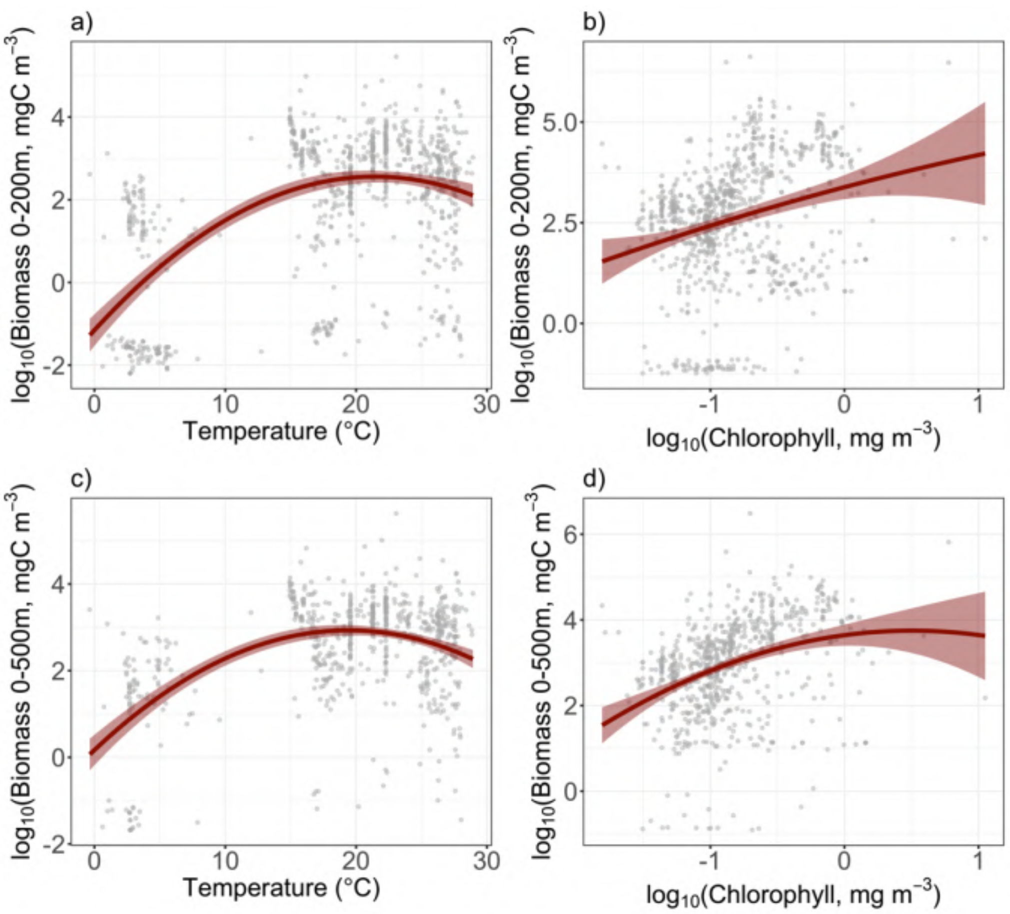

Fig. S7. Model fit to each of the predictor variables for giant rhizaria with partial residuals. Top row is 0-200m, bottom row is 0-500m.

Total mesozooplankton biomass

For each grid square, we use temperature, bathymetry and chlorophyll and the wet weight factor level to calculate the depth-integrated wet weight biomass of mesozooplankton (excluding rhizaria) over the water column in each grid square to get a total global wet weight biomass is 8.85 Pg (95% confidence interval for this estimate is ×/÷ 7.5-fold). For giant rhizaria, we use temperature and chlorophyll and a 10-1 carbon to wet weight conversion (*7*) to calculate a global rhizaria biomass estimate of 2.2 Pg wet weight (95% confidence interval for this estimate is ×/÷ 8.5-fold). This is close to the estimate of 0.204 Pg carbon weight from (*65*), when converted to wet weight. Rhizaria biomass in the top 200m was 1 Pg wet weight (using a 10-1 wet weight to carbon conversion), which is also close to Biard et al.'s estimate of 0.089 Pg carbon weight (*65*). We combine our global mesozooplankton (excluding giant rhizaria) with our global giant rhizaria biomass estimate to obtain a total mesozooplankton biomass estimate of **10.9 Pg** wet weight biomass, with a 95% confidence interval for this estimate of ×/÷ 7.7-fold.

Macrozooplankton
Data derive from 36,268 observations from MAREDAT, Moriarty, 2012 (*66*)
https://doi.pangaea.de/10.1594/PANGAEA.777398

After quality controlling the data (e.g., filtering NA observations for biomass, volume filtered, depth sampled), we were left with 23,815 observations We also removed about 8000 observations that recorded zero biomass density because most of the observations (34,938 out of 36,268) were converted from abundance to biomass using conversion equations, but only if the observations recorded species-level information (so as to use median species size). This means that many observations were recorded as zero carbon biomass even though there was non-zero abundance recorded, because species-level information was not retained for that sample, meaning they are not true zero observations.

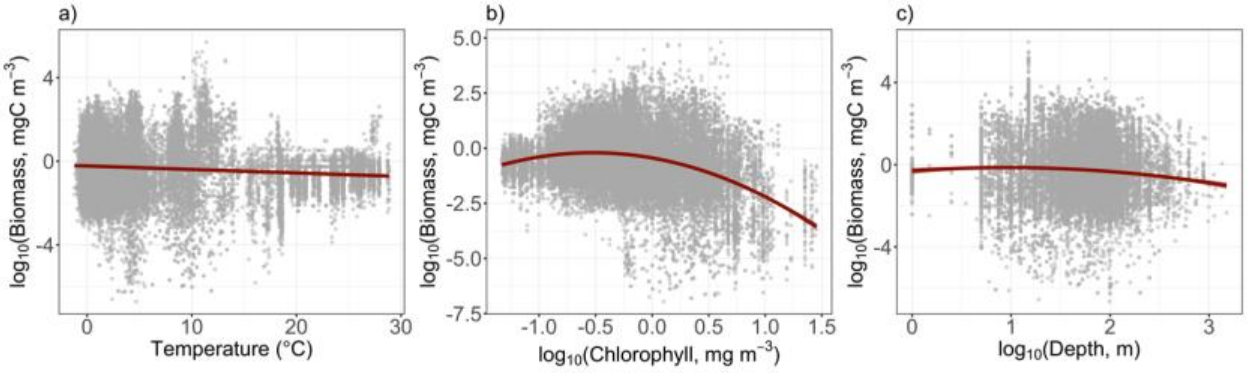

Fig. S8. Model fit to each of the predictor variables for macrozooplankton, with partial residuals.

We used a generalized linear model, with a second order polynomial relationship between $\log_{10}$ zooplankton biomass, a linear relationship with $\log_{10}$ chlorophyll temperature, and a second order polynomial for $\log_{10}$ sample depth (Fig. S8). All predictors are significant, and the R^2 for this model is 13%, with a very wide spread of residuals. Similar to microzooplankton, macrozooplankton biomass declines at high chlorophyll, but also similar to microzooplankton most of the data comes from areas with less than 1mg m^{-3} of chlorophyll a.

For each grid square, we use temperature, bathymetry and chlorophyll and a carbon to wet weight conversion factor of 10 (*7*) to calculate the depth-integrated carbon biomass of macrozooplankton (g/m^3) over the water column in each grid square, to get a total global wet weight biomass of **1.5 Pg** (95% confidence interval for this prediction is ×/÷ 11.4-fold).

Fish

In the analysis here, 'fish' include true fish (bony and cartilaginous) as well as invertebrates of the same size range (mostly cephalopods). Fish include epipelagic, mesopelagic and demersal organisms as well as some benthic organisms that are not covered by zooplankton. We split this group into two groups; mesopelagics and non-mesopelagics, and estimate both separately. We then

compare our cumulative estimate of these groups with published estimates to assess how our value compares with other studies.

Mesopelagics

Mesopelagic fish live in the mesopelagic zone (200-1000m) of the global ocean. Although difficult to sample directly, these fish can be detected by the reflectance of acoustic signals from sonar, and this reflectance can be used to estimate their biomass (*75*). However, acoustic estimates are sensitive to assumptions about the physiological structure of individual mesopelagic fish, as well as the presence of siphonophores (cnidarians), which have a high acoustic reflectance but low biomass. To address these issues, we used results from (*75*), who developed a model to explore how the percentage contribution of siphonophores to the acoustic backscatter as well as different assumptions about the proportion of fish with gas bladders (which have high reflectance but low biomass), would impact global estimates of mesopelagic fish biomass from acoustic surveys. Taking these uncertainties into account, they reported a range of 1.8 – 16 Pg wet weight (the lower and upper quartile from 500,000 simulations) for the biomass of mesopelagic fish from 70° north and south across the global ocean. Taking the geometric mean of this range, as well as the maximum fold-change from the upper and lower range bound to the geometric mean for our 95% confidence interval estimate, we obtain a global estimate for mesopelagic fish biomass of **5.4 Pg** with a $\times/\div$ 3-fold 95% confidence interval.

This estimate is similar to (*42*) estimate of 5 Pg wet weight (assuming a wet-weight to carbon conversion of 10), which was based on the geometric mean of a global acoustic survey estimate of 1.5 Pg C (which is probably an overestimate given it assumes most mesopelagic fish have no gas bladder and so have high density) and with a net trawl survey estimate of 0.15 Pg C from (*115*) (which is probably an underestimate given the ability of mesopelagic fish to avoid survey nets; (*97*)). Our estimate is also within the range of uncertainty given by (*12*), who reported a mesopelagic fish biomass between 40° north and south of 2.4 Pg, with approximately a 5-fold uncertainty.

To define the size range of mesopelagic fish, we used the minimum and maximum recorded sizes from three datasets of mesopelagic fish length-weight measurements (*116–118*). The combined datasets contained samples from the trophic and equatorial Atlantic as well as the Southern Ocean and gave a body size range of 0.01-500 g for mesopelagic fish.

Non-mesopelagics

For our estimate of the cumulative biomass of epipelagic and demersal fish, cephalopods and large benthic invertebrates, we used spatially-resolved global estimates from two process-based marine ecosystem models (*39*, *40*), chosen due to their comprehensiveness and use of data constraints. The first estimate comes from the Bioeconomic mArine Trophic Size-spectrum model (BOATS; (*39*)), a size-based global model that represents harvested organisms (including epipelagic and demersal fish, squid and benthic invertebrates) from 10 g-100 kg, using average temperature in the top 75m with integrated primary production in the water column as environmental inputs. The second estimate comes from the FishErIes Size and functional TYpe model (FEISTY; (*40*)), which represents the global biomass of epipelagic and demersal fish, and benthic invertebrates from 1 mg to 125kg, using a functional-type framework, with sea surface temperature, zooplankton carbon concentration and particulate organic carbon flux to the sea floor as environmental inputs.

Both models are constrained with catch data, and each are able to reproduce global-scale patterns in catch of pelagic and demersal fish, cephalopods and benthic invertebrates that agree with empirical estimates. These models are members of the FISHeries and marine ecosystem Model Intercomparison Project (FishMIP), and we used publicly available output from these models to derive our global biomass estimates from simulations where each model is forced with environmental inputs from the CESM-BGC1 earth system model (both models' output available at https://www.isimip.org). For each model, we calculated their global average biomass using the decadal average from 1850-1860 from simulations of pristine ocean biomass with no fishing. We chose this decade because it was the furthest in the past that both models have been run through, and it is far enough back in time that anthropogenic climate impacts are not discernible from existing climate variability (*74*).

Only FEISTY covered the size range from 1mg to 10 g, so we used this model to calculate a global biomass estimate of **1.35 Pg** for pelagic and demersal fish, and benthic invertebrate biomass in this size range. From 10 g to 100 kg, the FEISTY model gave a global biomass estimate of 2.25 Pg, and the BOATS model provided an estimate of 5 Pg. Taking the geometric mean of these two values, we estimate the global biomass of pelagic and demersal fish, benthic invertebrates and cephalopods from 10 g to 100 kg to be **3.6 Pg**. To obtain the biomass of fish with body sizes greater than 100 kg we used the scaling relationship between biomass and body size from (*20*), who used macroecological theory from (*32*) to define the relationship between biomass and body size as a power function with an average exponent of -0.06 (assuming an average ecosystem trophic transfer efficiency of 0.125, and an average predator-prey mass ratio of 1000). According to this scaling, total biomass in any given order of magnitude size range is 88% of the biomass in the preceding order of magnitude range. Using this relationship, and their reported median biomass of all fish between 1 g and 1 tonne of 4.9 Pg, we were able to obtain a global biomass estimate of total biomass from 100 kg-1 tonne (covering groups such as adult salmon and tuna) of **0.57 Pg**, from 1 tonne-10 tonne (covering groups such as sunfish, rays and sharks) of **0.48 P**g. The fish component of this biomass was then calculated by subtracting mammal biomass (derived below).

To calculate the uncertainty range for our estimates of non mesopelagic fish biomass, we follow the approach of (*42*), who took the 90% confidence interval of global fish biomass from (*20*) as representative of the uncertainty of their non mesopelagic fish biomass estimate. Jennings and Collingridge (*20*) calculated a median biomass of fish between 1e-5g and 1 tonne of 14 Pg, with a 90% confidence interval of 2.9 Pg to 46 Pg. Using this interval, we calculated a ×/÷ 5-fold 95% confidence interval for our estimate of non-mesopelagic fish.

Comparison of total fish biomass with other global estimates

When combined, our estimate of global fish biomass is within the range of estimates reported in previous studies. For example, our estimate of total biomass from 1g to 1 tonne is 8.2 Pg, almost double the 4.9 Pg estimate of (*20*) but within the 50% uncertainty interval for their estimate (2 – 10.4 Pg). More broadly, for all organisms from 1e-5g to 1 tonne, covering mesozooplankton, macrozooplankton and all fish, we obtain a global biomass estimate of approximately 23 Pg, which is almost double the 14 Pg estimate of (*20*), but still within the 50% uncertainty interval of their estimate for this size range (7.7 Pg – 23 Pg). Finally, (*42*) estimated the global biomass of mesopelagic and pelagic fish, benthic organisms and cephalopods to be approximately 10 Pg wet

weight (assuming 10-1 wet weight to carbon conversion), which is close to our total estimate for these groups of 10.9 Pg.

Table S5. Summary of fisheries models, their size ranges and global fish biomass estimates.

Marine model	Size range (g)	Earth system model	Biomass (Pg)
BOATS (Carozza et al., 2016 (*39*))	$10 – 1x10^5$	CESM1-BGC	4.94
FEISTY (Petrik et al., 2019 (*40*))	$1x10^{-3} – 1.25x10^5$	CESM1-BGC	3.55

Mammals

Marine mammal biomass was estimated from species-specific surveys for the majority of all known species. These are usually underestimates due to limited observation over portions of a species full geographic distribution, or else due to the inherent difficulties of counting individuals that dive for long periods or actively evade observers. Moreover, we seek both current and "pristine" numbers, the latter of which might characterize the pre-whaling period, before any observations of population numbers were systematically taken. This necessarily involves guesswork, but the range of possibilities is narrowed by making use of multiple independent estimates for the current abundance, body mass, and geographic range, along with growth rates and human capture data. These complementary data are available for most marine species, as well as for most non-marine mammals, against which the marine estimates can be compared.

Population data of varying quality exist for more than 2/3 of extant species of marine mammals. Although there are more than 130 species of marine mammal (WoRMS (*119*)), we excluded some species (e.g. fish-eating bats) that did not physically reside in the ocean, leaving us with 126 marine mammal species. Nearly all current estimates of marine mammals are from IUCN (*47*) (*n*=87). We estimated "pre-whaling" population counts by taking larger valued estimates or upper confidence intervals when extensive capture levels have been reported, or else from expert opinion or modeling studies (*n*=83). For many of the larger whales, we relied on estimates in (*48*), which is in part based on a study by (*67*). When no additional information was available beyond current estimates, we assumed pristine numbers were the same as exist currently. We believe this approach is more likely to underestimate abundance than overestimate it in most instances, but they provide among the most complete tallies of currently available data for marine mammal biomass yet assembled.

1.

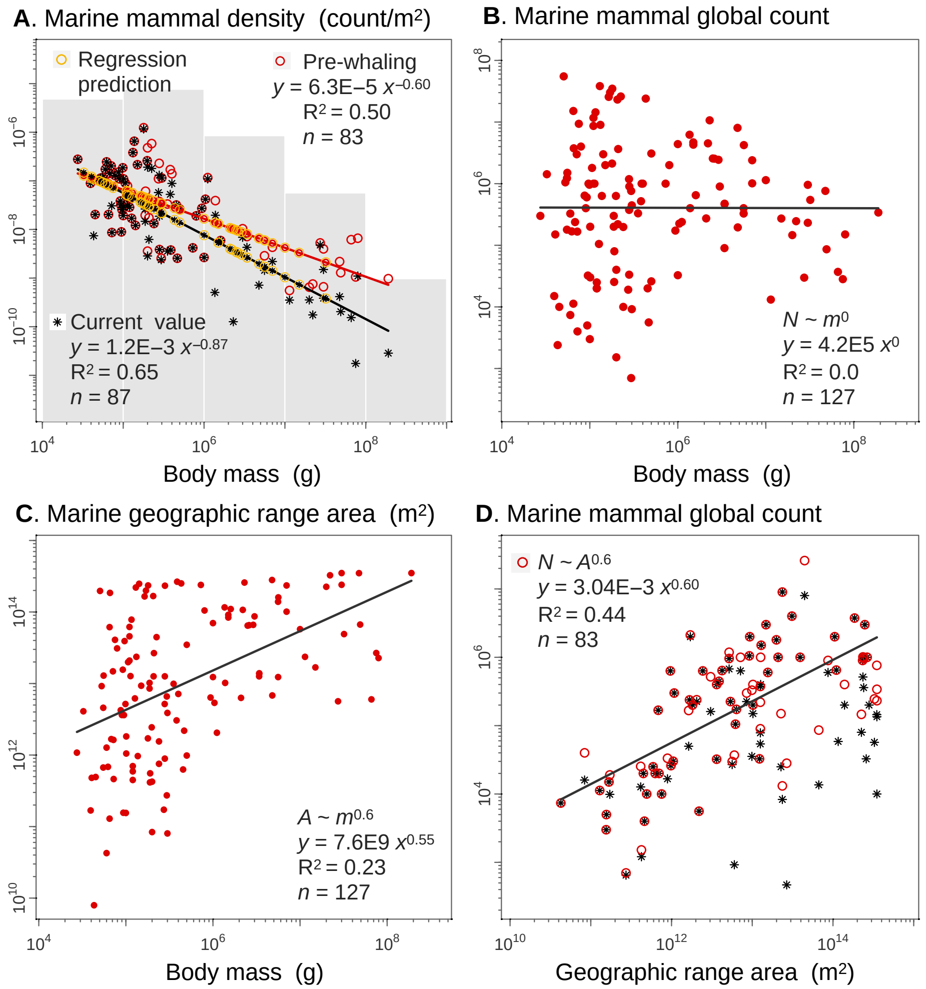

A.	$\boldsymbol{N/A \sim m^{-0.6}}$	Pop. density vs. body mass	m	Body mass
B.	$N \sim m^{0}$	Global count vs. body mass	N	Global count
C.	$A \sim m^{0.6}$	Range area vs. body mass	A	Range area
D.	$N \sim A^{0.6}$	Global count vs. range area	N/A	Pop. density

Dividing B by C, gives: $N/A \sim m^{0}/m^{0.6}$ ➔ $\boldsymbol{N/A \sim m^{-0.6}}$ (matches A)

Dividing D by area, gives: $N/A \sim A^{0.6}/A$ ➔ $N/A \sim A^{0.6-1}$ (substitute C in RHS)

Multiply mass exponents: $N/A \sim (m^{0.6})^{(0.6-1)}$ ➔ $\boldsymbol{N/A \sim m^{-0.24}}$ (does not match A)

Fig. S9. Marine mammal population regressions. A. Mammal population density is calculated by dividing the global species count by geographic range area. Regressions for both pre-whaling estimates (red circles) and current densities from IUCN (black stars) allow species with no global data to be predicted (yellow circles). The grey bins sum total marine mammal population density in each size class. **B.** Total global counts of mammal species are uncorrelated with body mass ($N \sim m^{0}$). **C**. Mammal geographic range area shows a positive relation with body mass ($A \sim m^{0.6}$). **D**. Global mammal population counts show a positive relation with geographic range area ($N \sim A^{0.6}$). Regression line is to pre-whaling values (red circles) and we did not account for shifts in range size. Regressions B to D show considerable scatter, giving different predictions for the regression in A. For example, pre-whaling density (A; $N/A \sim m^{-0.6}$) is consistent with combining B and C, ($N/A \sim m^{-0.6}$), but is not consistent with combining C and D ($N/A \sim m^{-0.24}$).

To provide population estimates for the 1/3 of species that have not been surveyed, we used regression predictions for the 2/3 of species for which observations are available. These data and associated species body mass and geographic range area were used to build relations between mammal population density and body mass. By dividing global population counts by geographic range area, we obtain mean population density, which we regressed against body mass for the 2/3 of marine mammals for which empirical counts exist (Fig. S9 A). These regressions were used to estimate pristine and current densities for the remaining 1/3 of species for which no current or prior estimates exist. Mean body mass estimates were obtained from (*120*), while range areas were obtained from shapefiles from (*47*, *121*) for all 126 marine mammal species. This allowed us to estimate the population density over their range for the remaining 40 some species (yellow points in Fig. S9 A), and to calculate their total numbers by multiplying by geographic range. While marine mammal species generally exhibit poor relations between global count vs. body mass (Fig. S9 B), geographic range area vs. body mass (Fig. S9 C) and global count vs. geographic range area (Fig. S9 D), their population density vs. body mass relation (Fig. S9 A) is comparatively robust for both the pre-whaling period and current estimates. Nonetheless, these regression predictions for species abundance without extensive surveys tend to be considerably higher, sometimes by orders of magnitude, than the estimates listed in (*67*), most of which are species with the lowest confidence and limited survey over their geographic distribution (see Supplementary Data file).

Mammal population densities and population biomass are also not notably different from terrestrial species, but tend to fall below the terrestrial body mass regression for both pre-whaling and particularly for current estimates (Fig. S10 A and B). The regression of terrestrial and pre-whaling densities has an exponent of −0.9, which is considerably steeper than what has been shown over a more limited range to be near −0.75 (*34*, *122*, *123*). Marine mammals, and particularly whales, extend the terrestrial mammal size-density relationship by a full two orders of magnitude.

Marine mammal abundances may be underestimated for many reasons, including i) difficulties in counting individuals hidden deep under the surface, ii) poor estimates of geographic range areas used to estimate densities or iii) centuries of human capture. Disentangling these many factors is not possible with currently available information. For reference, we also show how geographic range area vs. body mass compares between terrestrial and marine mammals, with all body masses capable of occupying the entire surface of land or sea, but larger animals apparently incapable of occupying very small areas (Fig. S10 C). We also show for all mammal species how species diversity (count of species in each size class) compares between terrestrial and marine species (Fig. S10 D).

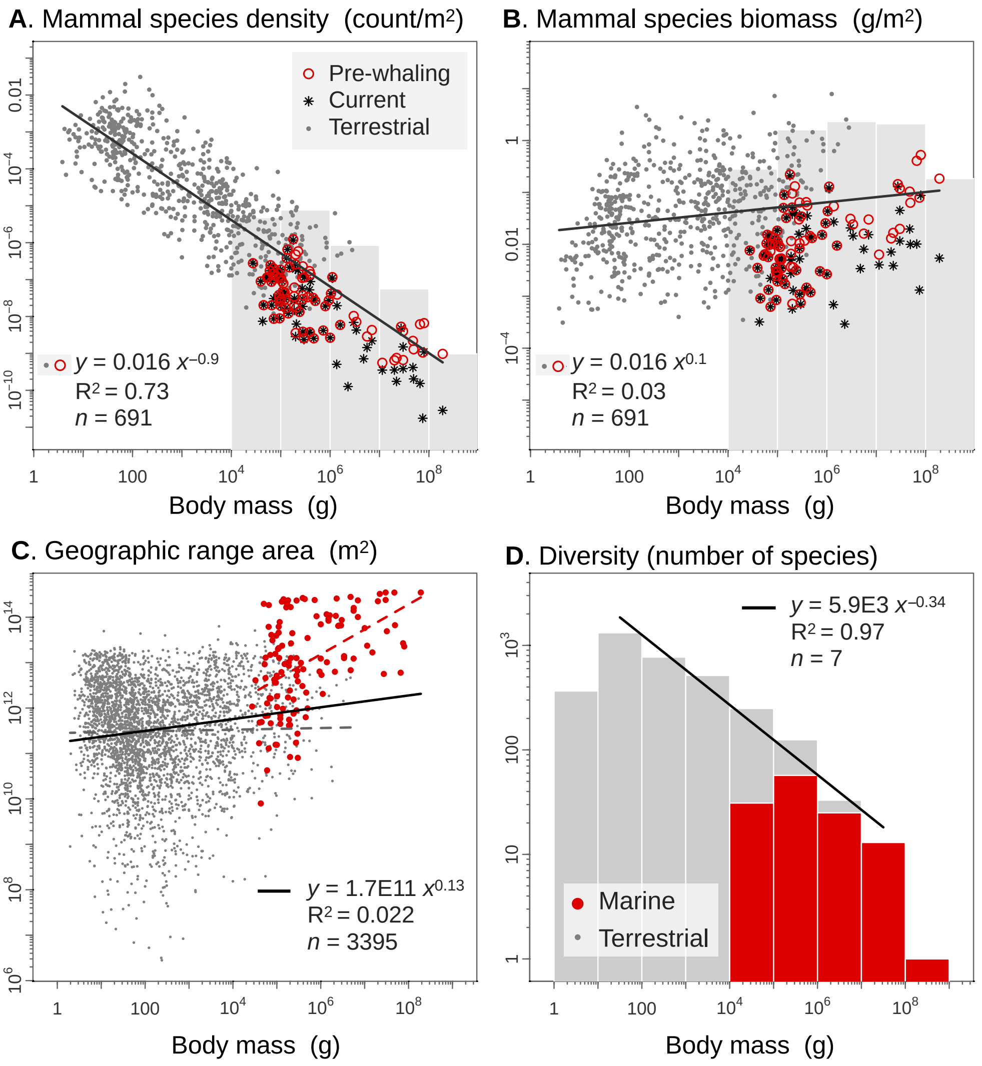

Fig. S10. Comparing marine and terrestrial mammal body mass regressions. A. Marine mammal population density from Fig. S9 A are compared with (mostly) terrestrial mammal species (grey circle). The regression line is for terrestrial and pre-whaling estimates (excludes current densities). **B**. Mammal population biomass is calculated by multiplying densities from A and body mass. The grey bins represent total marine mammal population density sums over each order of magnitude size class. **C.** Marine (red, from Fig. S9 C) and terrestrial (grey) mammal global geographic range areas. **D.** Mammal diversity (species richness) across order of magnitude size classes. The regression line excludes the smallest and largest (blue whale) size classes.

Our measure of uncertainty for large mammals derives from considering extensive additional data that we include in our dataset, but are not shown in the figures. In addition to "pre-whaling" and current best estimates of marine mammals, we made use of additional current estimates of marine mammals also listed on IUCN species pages (*47*), as well as data drawn from Appendix 4 of (*67*), which is derived from some 275 published sources, and lists minimum, mean and maximum values

for 115 of 126 marine mammal species. Many of these estimates are reportedly of very low confidence over a limited geographic range, and differ from our current best estimates to varying degrees, but tend to be within a factor of three of current values. It is more difficult to obtain a measure of uncertainty for pre-whaling abundance, but even the most extreme species specific values are rarely more than a factor of ten different from other estimates, and on average are within a multiplicative factor of 4.3 of maximum current values from (*67*). Pooling all estimates including those of (*47*) and the minimum, mean and maximum current values of (*67*) gives a mean fold uncertainty of 2.2 (following (*41*, *42*)). However, these separate estimates are not necessarily independent, and our knowledge of the pre- whaling period is necessarily vague. We considered a fold uncertainty value of 5 to be cautious, and include all additional marine mammal estimates in the Supplementary Data file.

Further assumptions about how we chose to deal with uncertainty and our regression methods are covered in the section "Statistical considerations", below.

Human impacts on the biomass spectrum

The most dramatic human impacts on marine biomass to date have been the hunting of wild animals, including fishing, sealing, and whaling. Climate change, on the other hand, is rapidly growing in importance and is expected to be a major driver of change in abundance over the current century. Because dramatic fishing impacts have already occurred and regulation efforts are now slowing the rate of overfishing in many parts of the world (*124*), we use empirically-informed estimates of the impact that fishing has had to date, focusing on the depletion caused by industrial fishing up until the early 2000s. We compare this with the climate change impacts expected under a pessimistic scenario for the 21st century, assuming that the impacts of fishing and climate on biomass are approximately additive, with little non-linear interaction between them (*50*, *125*). Thus the estimated historical fishing impacts are summed with projected climate changes to provide an estimate of the total impacts that would occur in future if the effective fishing effort remains approximately constant. Although humans have also impacted biomass by physically modifying habitats, most importantly coastal regions, estuaries and waterways used in anadromous fish migrations (*126*, *127*), we do not address these directly.

Annual mammal and wild fish harvests were estimated at 0.12 Gt from (*58*), while total annual human consumption is estimated at 4.7 Gt from (*128*), making human harvests over the ocean a small fraction < 3% of what humans consume globally. We use values for total human population of 1 billion in 1800, 8 billion currently and 11 billion in 2100. We multiply these values by a reference human being of 60 kg to get biomass values for humans shown in Fig. 3 A.

Fishing impacts

Fishing has had a major impact on the biomasses of fish (including true fish as well as targeted invertebrates). The available estimates that have been made for the global biomass depletion by fishing do not explicitly address changes in the distribution of biomass depletion over the size-spectrum, however, some local studies have addressed the size-distributed impact. Thus, we first consider the global fish biomass depletion, and second the changes in the size distribution based on local observations.

Biomass decline

There are many local and taxon-specific estimates of the decline of fish biomass caused by fishing activity, but relatively few global estimates. Furthermore, the long history of modification of ecosystems by fishing, which often began long before data was collected, leads to a problem of shifting baselines (*129*), making it difficult to reconstruct the pristine state. We include two different global estimates that approximately target the timespan of 1950 to 2000, each of which is methodologically independent.

1. Worm and Branch (*46*) present a compilation of fish stocks assessed by scientific management agencies. These covered only 20-25% of fished stocks at that time, but suggested that, for these stocks, only 31% of the pristine biomass remains. This is consistent with fishing pressure being exerted slightly in excess of that which would produce maximum sustainable yield, generally expected at roughly 40% of pristine biomass (*130*). Fishing pressure generally occurs on fish >10 g, and so this estimate is applied across all fish larger than 10 g. A similar value is obtained from (*131*) using a process model of the global fishery, including all potentially commercial fish >10g. These estimates are subject to uncertainty in the unfished biomass fraction, i.e. the proportion of the total fish biomass that is not harvested. This unfished biomass is not included in stock assessments, and is absent from global fishery models. We are not aware of published comprehensive assessments of this unfished fraction, but a recent model-based study provides a global estimate that 58% (±22%) of fish biomass is targeted by commercial fisheries (*132*), and that 42% is not targeted. In the absence of empirical data, we use this model estimate of commercial fisheries fraction to calculate the total fractional decline in fish biomass as follows: the commercial fisheries decline (0.31) multiplied by the commercial to total fisheries fraction (0.58), plus the fraction of fish not targeted by fishing (0.42), gives an estimated 60% of total fish biomass >10g remaining from pristine levels.

2. Christensen et al. (*45*) provide a compilation of more than 200 Ecopath models constructed for globally-distributed ecosystems and incorporating data on fish biomass. They fit a multi-variate linear regression to the simulated biomasses of predatory and forage fish in order to estimate the changes over time. They estimate a decrease in global biomass of all predatory fishes by two-thirds (60.2 - 71.2 %, 95% C.I.) over the century from 1910-2010, thus leaving only 33% of fish biomass >10 g remaining from estimated pristine levels. Christensen et al. (*45*) do not provide a size estimate for these fish, but here we assume this applies to all fish >10 g, commercial and non-commercial. They also suggest that small forage fish have doubled in biomass, potentially by as much as 130%, though we have not incorporated this suggestion, which remains an intriguing possibility.

We combined these two estimates for the fraction of fish >10g remaining from pristine fish biomass (60% inferred from (*46*), and 33% inferred from (*45*)) by taking their geometric mean, giving a value of 45% of fish >10g remaining.

Impacts on the size structure

As first demonstrated by (*38*), fishing has very strong effects on the size structure of exploited species and associated bycatch for three main reasons, all of which cause a steepening of the size-spectrum. First, larger fish are preferentially targeted given that most gears select sizes larger than some mesh cutoff size, increasing bycatch of larger organisms, and larger fish tend to be more desirable (*133*). Second, because large fish take many years to grow slowly from smaller fish, high rates of exploitation of young fish prevent the replacement of large, older fish (*39*). Third, because heavy exploitation favours quickly-reproducing individuals, there are strong evolutionary pressures towards smaller maximum sizes (*134*). Jennings and Blanchard (*134*) show trawling data for the heavily exploited North Sea, revealing a biomass slope of -1 (abundance slope of -2) over the range of 1-66 kg, which they interpret as a sharp steepening of the pristine slope of this size range of the benthic community by -1. Together, these factors have been shown to produce strong increases in the slopes of size spectra in most exploited communities (*2*).

We therefore calculated what size-spectrum slope would be consistent with the 45% depletion among the fraction >10g, assuming that fish ≤10g have not been significantly affected by current harvesting in the global average. This would require that the slope has become steeper by −0.17 for all fish >10g. Thus, we assumed no change in the size-spectrum for fish ≤10g, but above this the size spectra slope has been steepened by −0.17, account for the inferred fraction of fish remaining relative to pristine levels.

We note that different ecosystems seem to show different sensitivities of the size-spectrum to fishing. Demersal fish, which may have steeper size spectra slopes, appear to be relatively sensitive to harvesting (*135*). In contrast, coral reef size-spectrum slopes tend to be less steep, with baseline slopes > −1 and proving relatively robust to fishing pressure (*136*). Thus it appears likely that coral reef size structures have been less impacted than the global average, possibly due to the stabilizing effect of very large grazers. These observations serve to emphasize that our global inferences for fish declines will not likely apply to particular ecosystems and are necessarily a first order approximation.

In addition to steepening in the size-spectrum due to fishing, there have also been significant declines in large mammals, particularly whales. As described in the section "Data and methods" under "Mammals", along with accompanying data at the doi link provided in the first paragraph of this file, we compiled both pre-whaling and current abundance for more than 2/3 of all ~ 126 extant species of marine mammals. These data derive from over 275 primary sources, as well as meta-analyses of (*48*, *67*), as well as expert opinion and compilation from (*47*). While these data do not include all species, and are more limited for pristine species biomass estimates, they summarize the state of current knowledge and suggest some startling declines particularly among the largest size classes. In particular, these data suggest that among the smallest marine mammals (10-100kg), only 47% remain, but that progressively smaller fractions characterize the larger size classes, with possibly only 25% of mammals remaining in the 10 to 100 tonne size class, and only 3% of blue whales, making up the largest size class (*48*).

Combining the declines due to fishing and whaling results in significant changes of the size-spectrum, not only among harvested size classes, but with noticeable effects on the overall slope from bacteria to whales, as shown in Fig. 3 C and Table S6 (compare i and ii).

Climate projection impacts

To provide an illustration of expected impacts of climate change on the full size-spectrum of marine life, we assembled estimates of relative changes in biomass obtained by previously-published model projections for different taxonomic groupings. In all cases these studies compared the last decade of the 21st century with the last decade of the 20th century – thus, these do not estimate the total impact of climate change from the pre-industrial state, but rather serve as a consistent metric in the literature that is used here to gauge relative impacts across size.

Table S6. Human impacts on the size-spectrum. The global ocean regression (i) represents the estimated pristine (before 1800) size-spectrum, while regression (ii) is the size-spectrum estimated currently (2020) that includes the impacts of fishing, and (iii) is estimated under climate projected impacts to 2100.

	Size-spectrum regression	*n*	R^2	Constant	Exponent & 95% C.I.			Body mass range (g)		*p*–value
i	Before 1800, top 200m	23	0.999	6.4E+14	**-1.042**	-1.057	-1.027	10^{-14}	10^{9}	10^{-32}
ii	Current 2020, fishing	23	0.997	2.96E+14	**-1.087**	-1.115	-1.059	10^{-14}	10^{9}	10^{-27}
iii	Projected 2100, climate	23	0.997	2.45E+14	**-1.092**	-1.12	-1.063	10^{-14}	10^{9}	10^{-27}

At the root of all these projected changes are Earth System model simulations, that estimate the changes in ocean temperature, circulation patterns and biogeochemical cycling that will result from a given future trajectory of atmospheric greenhouse gases. Here we use the worst-case scenario trajectory, Representative Concentration Pathway (RCP) 8.5.

Rather than rely on a single model, we take the mean estimate from the ten climate models that participated in the fifth simulation round of the Climate Model Intercomparison Project (CMIP5) and included biogeochemical components, as analyzed by (*80*). The multi-model mean sea surface temperature (SST) changes from 1990-2000 to 2090-2100 under RCP8.5 is 2.73 ±0.72 °C. This temperature change would be expected to be representative of the average warming within the continually-mixed surface layer (the upper 20-80 m). At greater depths the warming would be less, given the time required for heat to penetrate from the atmosphere to the subsurface.

Changes in plankton concentration were calculated by the same Earth System models that estimate the physical changes, using ecosystem modules that were run in a fully-coupled mode with the ocean modules. According to the analysis of (*49*), these models simulate a global mean phytoplankton decrease of 6.1% ± 2.5% and a global mean zooplankton biomass decline of 13.6 ±3.0 %. These changes were applied uniformly across the full size ranges of phytoplankton and zooplankton, respectively.

Climate-driven changes in fish biomass are also provided by global dynamical process models, run offline using the output of the Earth System models. Typically the most important drivers are net primary production and water temperature. The multi-model analysis of (*50*) projected a 17 ±10.7% decrease in animal biomass, for the resolved animals >10 cm.

Unlike the case for the preceding groups, there are no global process models available for marine mammals. Instead, we use the statistical model of (*51*). These authors argued for a competitive niche of marine mammals that shrinks with rising temperatures, so that a 1 °C increase of sea surface temperature results in a 12% decline of marine mammal abundance (24% for pinnipeds). Thus, the CMIP5 estimated warming of 2.73 °C implies a 30% reduction in marine mammals and a 53% reduction in pinnipeds.

Finally, we were unable to find prior estimates for the relative changes in bacteria concentrations under climate change. Instead we estimated the changes using the temperature and chlorophyll dependence from the statistical model we developed to estimate global bacteria abundance, an approach analogous to that used by (*51*) for marine mammals. We obtained decadal averages of global sea-surface temperature and surface chlorophyll-a concentration for 1950-1960 and 2090-2100 from the GFDL-ESM2M and IPSL-CM5A earth-system models, forced with historical and RCP85 scenarios. We used these decadal averages as inputs to our statistical model of global bacteria abundance, and obtained an estimate of the relative change in bacteria abundance in 2090-2100 compared to 1950-1960 under the RCP85 warming scenario for each earth-system model. Contrary to other major groups, we estimate that global bacteria abundance will see a moderate increase of approximately 2.3% by the end of this century under an RCP8.5 warming scenario, compared to 1950-1960 levels.

While climate change projections could have significant unanticipated changes on marine ecosystem function, they appear to pale in comparison to the impacts on the size-spectrum resulting from fish and mammal harvesting, as shown in Fig. 3 B and Table S6 (compare ii and iii).

Statistical considerations

In this section, we outline the various ways of representing the size-spectrum, our fitting methods, and the sensitivity of our assumptions of group size distributions and group biomass to our overall findings.

Fitting size-frequency distributions

Unlike the size-frequency distribution of people, which follows the classic 'bell-curve' or normal distribution, a power law distribution is extremely right skewed, also referred to as heavy-tailed or fat-tailed. The power law distribution (also called the Pareto distribution) is more like a log-normal distribution but with no central mode, such that the smallest size class is overwhelmingly the most

frequent, and the largest size class is extremely rare, but orders of magnitude larger than the smallest (*59*, *137*, *138*).

A power law is revealed as a straight line when observations are plotted on log-log axes of size and frequency. The same data appears as a capital "L" on regular axes, and is not informative over larger size ranges. Many claims of underlying power law mechanisms have been made from the appearance of a straight line on log transformed axes, some of which are undoubtedly false (*79*, *139–141*). In the past decade, methods have been developed based on maximum likelihood that better evaluate if the underlying data conform to a power law based on more principled statistical methods, and claim to estimate the exponent with greater precision than ordinary least squares. Unfortunately, there has been considerable criticism of these methods (*79*, *140*, *141*), implying that the statistics of fitting power law distributions has failed to reach broad consensus.

An additional source of confusion is the proliferation of different ways of constructing these size-frequency distributions, which is not only extensive among aquatic ecologists, but also among the many other fields of science that study power law frequency distributions. Here we list several of the many ways that the size-spectrum can be represented (*2*):

- *Size-spectrum or abundance-spectrum*: This is the size-frequency distribution shown in Fig. 1, representing numerical abundance (y-axis) vs. size class (x-axis), both in logarithmic scale (including logarithmic bin width). This is a common way that the distribution of individual sizes is represented. A size-spectrum slope of −1 indicates that size and abundance are inversely proportional, i.e. individuals decrease in number by the same factor as they increase in body mass.

- *Biomass-spectrum*: This is the size or abundance spectrum multiplied by the body mass size-class, as shown in Fig. 2, and is also a common way that these distributions are represented. For example, abundance-spectrum slope of −1 is equivalent to a biomass-spectrum slope of 0. Biomass may be represented on logarithmic (Fig. 2 A) or linear axes (Fig. 2 B).

- *Normalized size-spectrum*: This is the size-spectrum but the count of individuals in each bin is divided (or "normalized") by the bin width (although bins appear to have equal width on log axes, they are in fact getting multiplicatively larger as size increases) (*2*). An abundance-spectrum (with log bins) slope of −1 is equivalent to a normalized size-spectrum slope of −2. The normalized size-spectrum exponent is equivalent to the probability density function exponent (*59*, *142*).

- *Normalized biomass-spectrum*: Similarly, this is equivalent to the biomass spectrum except that the biomass in each size-class is normalized (divided) by bin width. A biomass-spectrum (with log bins) slope of 0 is equivalent to a normalized biomass-spectrum slope of −1.

- *Cumulative distribution*: Instead of binning the data, the distribution can be expressed as the probability that a randomly selected individual is greater than or equal to a given size. The cumulative distribution is sometimes thought to be a superior way of representing the data, given that it eliminates the requirement for arbitrary decisions about how to construct bins (*59*, *139*).

- *Rank-size distribution*: Instead of plotting the cumulative distribution on the y-axis and size on the x-axis, we can instead plot the rank of the cumulative distribution against body mass. This is typically done by reversing the axes, so that rank is on the x-axis and size is on the y-axis. This is also known as Zipf's law (*59*, *138*). If we rank the sizes of individuals in order, then there are n individuals with frequency greater than or equal to that of the nth largest size. The rank and cumulative distribution are thus proportional, and since the axes have been reversed then the

exponents of the distributions are inverse, which in the particular case of a slope of −1 remains the same (see also (*143*).

In addition to these different ways of expressing the size distribution, ecologists have expressed size on the x-axis in terms of length (e.g. diameter), volume or mass (e.g. wet or dry mass) or energy density (e.g. kilocalories or watts). Although the exponent is unlikely to be much affected by conversions between volume, mass or energy density, the use of length as a measure of size yields very different exponent values. For example, if the size-spectrum uses length, and has a cumulative distribution function slope of $-\alpha$, then to convert to volume, the slope will be $-\alpha/3$. If the probability density function uses length and has a slope of $-\alpha$, then to convert to volume, the slope will be $-(\alpha+2)/3$. Although Sheldon et al. originally represented size in terms of length (equivalent spherical diameter; Sheldon et al. 1972, Sheldon and Parsons 1969), they binned their data in units of volume, so that converting length to volume would not alter the exponent.

Our estimated size-spectrum extends over a size range of some 23 orders of magnitude. Very few power laws in other fields of science, with the exception of perhaps astronomy, come anywhere close to these size ranges (e.g. see list in (*59*)). Many claimed empirical power laws that have later been claimed to be spurious under alternative statistical approaches typically span only two to five orders of magnitude. Our purpose was to show that the Sheldon hypothesis is largely validated (with two broad exceptions being bacteria and whales), rather than to provide precise estimates of the overall slope. Nonetheless, our best estimates of abundance in each size class exhibit a highly regular pattern over this extraordinary size range, such that different estimators of the exponent are quite similar, as shown in Table S7.

Table S7. Comparing exponents from different fitting methods. Regression statistics for the global ocean and that restricted to the top 200m are shown for ordinary least squares (OLS), with exponents from maximum likelihood (MLE) and reduced major axis (RMA) shown for comparison. "*n*" refers to the number of size class bins, R^2 is the coefficient of determination, "Constant" is the abundance when size is 1 g, and 95% C.I. is the 95% confidence interval for the OLS derived exponent.

	Size-spectrum regression	*n*	R^2	Constant	Exponent			95% C.I. (OLS)		Body mass range (g)		*p*–value
					MLE	RMA	OLS					
ii	Global ocean, top 200m	23	0.999	6.61E+14	**-1.028**	**-1.040**	**-1.040**	-1.054	-1.026	10^{-14}	10^{9}	10^{-32}
ii	Global ocean to seafloor	23	0.998	1.09E+15	**-1.033**	**-1.052**	**-1.051**	-1.072	-1.03	10^{-14}	10^{9}	10^{-27}

We show statistics obtained from ordinary least squares (OLS), and compare these exponents with those of maximum likelihood (MLE; following methods outlined in (*79*) for binned data), and reduced major axis (RMA; following methods outlined in (*78*, *144*)). The latter method partitions error in the data equally to both x- and y-axes (unlike OLS, which assigns all error to the y-axis), and so is not really appropriate for our data. Although there is undoubtedly some error in our assignment of different major groups to different size classes, the vast majority of our uncertainty lies in our ability to estimate abundance over the global ocean in each size class. Nonetheless, we report RMA exponents for reference, and note the similarity in the exponents among all methods (OLS, MLE and RMA).

Sensitivity analysis of group size distributions

Most research over the past 50 years has revealed that the biomass distribution within major groups is approximately invariant with body mass (Table S3, and references therein). Nonetheless, to make such an assumption *a priori* across each of our 11 major groups (see Table S2) would potentially weaken our ability to test the hypothesis of invariant biomass across all groups together. As we show, however, alternative assumptions about the size distribution within major groups, even those that are quite unrealistic, all give very similar global patterns from bacteria to whales.

For example, instead of assuming an invariant biomass within each major group, we can assume a log-normal distribution of biomass across each group, which tends to characterize more resolved taxonomic groups, but we are not aware of the log-normal describing the size distributions of any of the major groups we have distinguished (see Tables 1, S2), with the exception of bacteria in the Sargasso Sea (*99*). Nonetheless, this assumption served to illustrate that quite different major group size distributions have little effect on the overall size distribution.

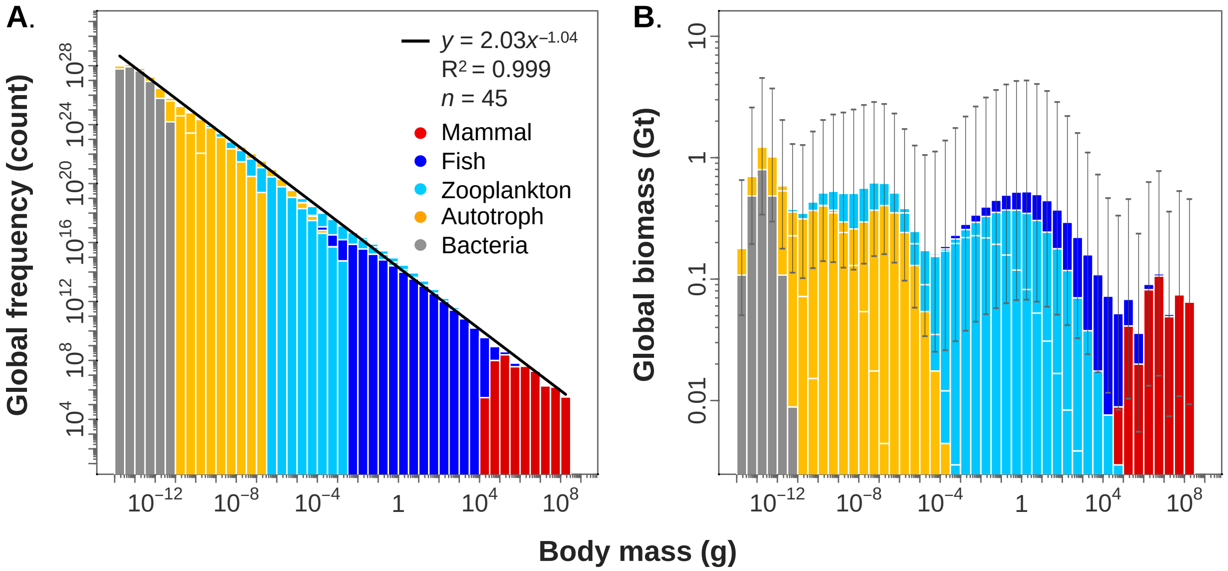

Fig. S11. The size-spectrum assuming a log-normal distribution of biomass. Total global frequency (**A**) and biomass (**B**) vs. body mass size-class assuming each of eight major groups are log-normally distributed with respect to size. We have assumed that our best estimate of the logarithm of size range for each group corresponds to four standard deviations. The bin width can be modified arbitrarily without altering the slope (shown here are half an order of magnitude size bins). Depending on how groups are stacked within a given size class determines which group is given prominence on the logarithmic plot (plots in A and B are the same data, but are stacked differently so that they appear different).

We assumed the size range of each major group corresponds to four standard deviations, and that the total biomass independently estimated for each group constrains the distribution. We also binned groups into half order of magnitude width, though this has a negligible effect on our results. We show that the slope for the size-spectrum and the biomass spectrum is essentially unchanged from Figs. 1 and 2 (see Fig. S11). Similar analysis using other long-tailed distributions and binning criteria yield little differences in slope across all groups.

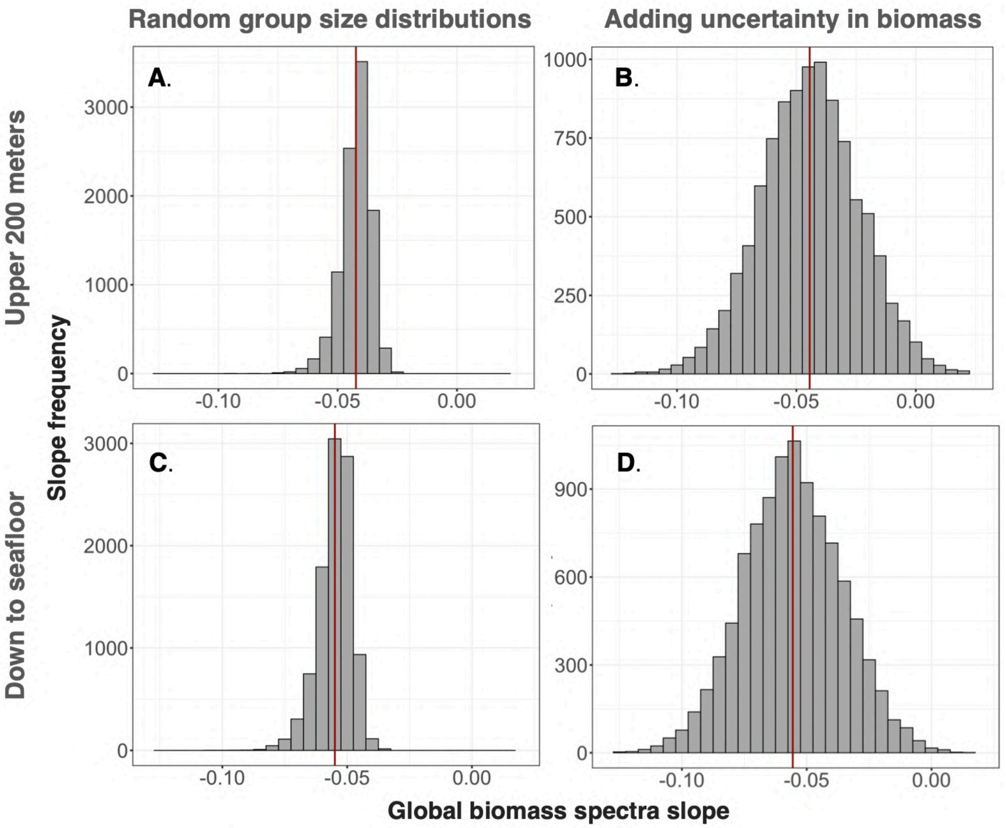

Fig. S12. Biomass spectra slopes under different assumptions. Spectra slopes are obtained from 10,000 simulations where we assume no knowledge of the distribution of biomass for each of 12 major groups, listed in Table S2. **A** and **B** show exponent values for the upper 200 meters of the ocean, while **C** and **D** show values for waters to the seafloor. In A and C, we use our best estimates of group biomass, and allocate this at random to different size classes within the group. In B and D, we additionally randomize the assignment of group biomass based on our uncertainty bounds shown in Fig. 2 A.

We have also undertaken sensitivity analysis on the size distribution of major groups and find that even quite extreme variation in group size distributions still yields slopes centered on the slopes reported in Figs. 1 and 2, with little variation about the mean.

When we assume that the biomass in each group is uniformly distributed within each group's size range, we obtain a slope of -0.060 for the total depth integrated global biomass size-spectrum, and -0.040 for the global biomass size-spectrum in the top 200 m. To assess the uncertainty of these slope estimates, we started by assuming that the only knowledge we have is the size ranges of the different groups, but no knowledge of how the biomass of each group is distributed within its size range. To do this, we generated for each group a random allocation of total group biomass across the log-equal bins in its size range, with the total allocation summing to 1. Fig. S12 A and C are the distribution of slopes after 10,000 simulations for the top 200 m, and the entire water column, respectively. For the top 200m, we obtained a normal distribution with mean -0.042 and a standard deviation of 0.006. This gives a 95% confidence interval for the slope of the total global biomass-spectrum in the top 200m of -0.054 to -0.030. For the global biomass-spectrum for the entire water column, we obtained a normal distribution with mean -0.054 and a standard deviation of 0.007.

This gives a 95% confidence interval for the slope of average global biomass from bacteria to whales across the entire water column of -0.068 to -0.040.

We not only have uncertainty in the distribution of biomass for each group within its size range, but we also have uncertainty in our estimates of the total biomass for each group. In addition to considering the uncertainty in the slope of average biomass for each group, we now consider the additional effect of uncertainty in our estimates of the total biomass for each group. To do this, we added an additional step where for each group we obtained a random total biomass estimate from a log-normal distribution centred at the mean biomass estimate for that group, with standard deviation calculated from the 95% confidence intervals for the group. Fig. S12 B, C are the distribution of slopes for 10,000 simulations for the top 200m and the entire water column, respectively. For the top 200m, we obtained a normal distribution with mean -0.049 and a standard deviation of 0.021. This gives a 95% confidence interval for the slope of the average global biomass in the top 200m from bacteria to whales of -0.085 to -0.003. Across the entire water column, we get a normal distribution with mean -0.056 and a standard deviation of 0.020. This gives a 95% confidence interval for the slope of average global biomass from bacteria to whales across the entire water column of -0.095 to -0.017.

Slope dependence on group biomass

Our global maps of biomass for each of nine major groups allow us to estimate the biomass (or abundance) spectrum slope for nearly every grid point over the global ocean, where all major groups have been estimated. These data exclude nano-zooplankton and mammals, though they represent a small fraction of the total biomass and so may not play so dominant a role in affecting the biomass spectrum. Fig. S13 is a map of the biomass spectra slopes at all the grid points where we have data for each major group from bacteria to fish. We assumed uniform biomass across each major group size range, though as discussed above, this has little effect on the resulting spectra slopes (see previous section). We then summed across major groups in each size class for a total of 33,821 regression slopes. These slopes are almost all in [-0.01, -0.05], which are consistent with the sensitivity analysis described in the previous section (see the histogram of slopes above the map legend in Fig. S13).

Although this analysis suggests the size-spectrum appears to broadly hold regionally, at 1° spatial resolution, we caution against drawing strong conclusions from these findings for the following reasons. First, spatially resolved size-spectra do not include several major groups (mammals and nano-zooplankton). For these groups, only global estimates of biomass are available, potentially biasing more spatially resolved size-spectra. Moreover, many mammals, and fish aggregate and migrate over vast distances, such that even the relatively large 1° lat-long cells of the ocean may not be sufficiently large to capture these variations. Second, while most of our data are widely distributed geographically (Fig. 1 A), they are still very patchy for most major groups. We expect that geographic variations will be partially normalized through aggregation over the entire ocean, since we do not expect any systematic bias in our global interpolation. Nonetheless, the patchiness of the data may mean that undersampled regions do not provide reliable estimates of the local size spectrum. Third, an analysis of the local size-spectrum slope dependence on different major groups revealed that fish biomass appears to dominate slope estimates (Fig. S13 E), described below.

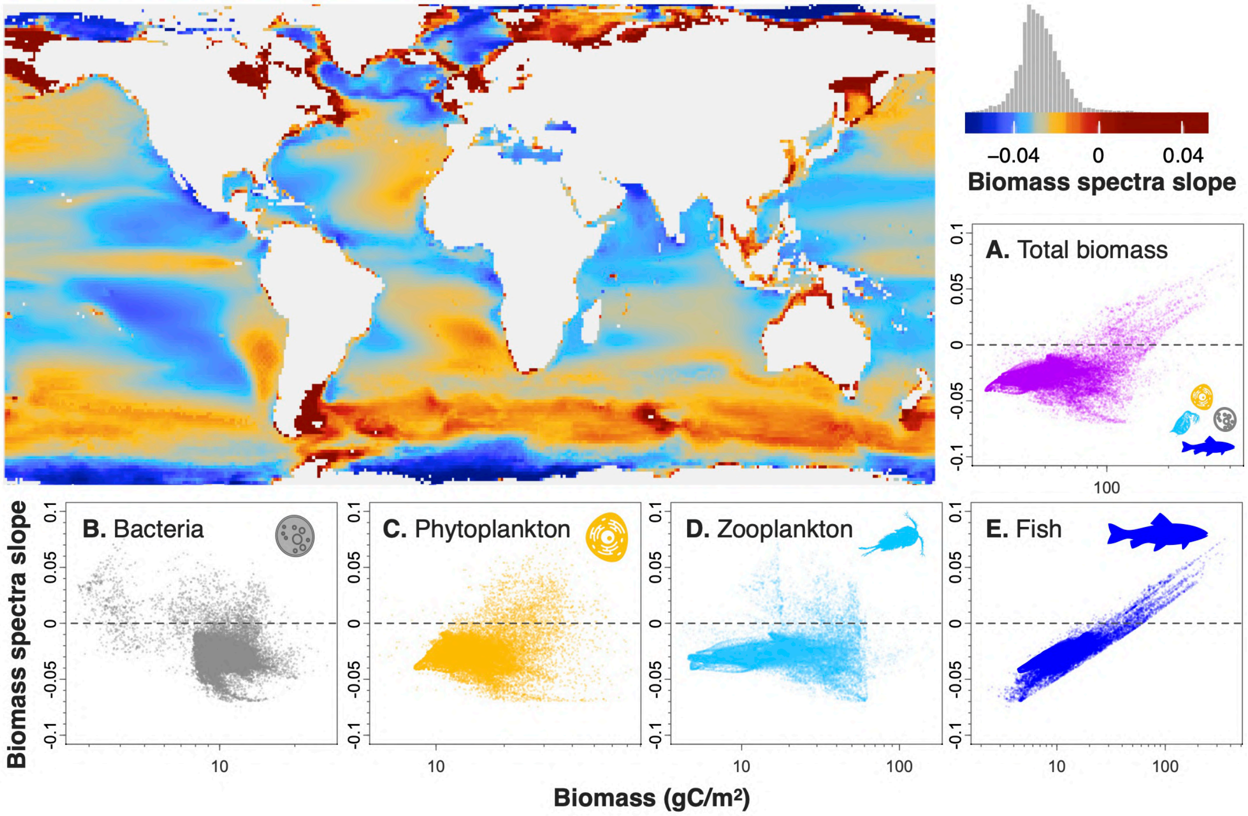

Fig. S13. Geographic distribution of biomass spectra slopes and their dependence on major group biomass. The biomass spectra slope is calculated for 33,821 regions in the ocean, with the vast majority of slopes between -0.05 and -0.01, as shown in the legend histogram. The colors in the map denote the slope, while colors in the plots denote major taxonomic groups. For each ocean region, the biomass spectrum slope over all groups is plotted against the biomass of each major group separately, excluding mammals. **A.** Biomass spectrum slope vs. the total biomass sum over all major groups. **B.** Biomass spectrum slope vs. bacteria biomass. **C**. Slope vs. phytoplankton biomass. **D.** Slope vs. zooplankton biomass. **E.** Slope vs. fish biomass. Biomass spectra slopes appear to have a strong, almost proportionate dependence on the logarithm of fish biomass. Fish also exhibit an order of magnitude larger biomass range than other groups.

We checked how this relatively small variation in slope was related to the biomass of each major group (Fig. S13 B-E). Although there is almost no slope dependence on total biomass, nor on most major groups, there does appear to be a strong slope dependence on fish biomass (Fig. S13 E) in different regions of the ocean. The slope appears to have a strong proportional dependence on the log of fish biomass. Unfortunately, fish also happen to be our least well resolved or constrained group, and so it is difficult to know whether this is representative of biological reality or an artefact of how fish biomass is estimated.

If this slope dependence on fish biomass is legitimate, it indicates the slope gets more negative in more oligotrophic systems, which is consistent with comparative empirical research among plankton (*2*), but contradicts empirical research among fish in northern lakes (*145*). Fig. S13 E implies that fish get more abundant faster than other groups as we go to more eutrophic systems (and less abundant faster towards oligotrophic). If oligotrophic has a more positive slope than

eutrophic (regardless of actual slope values), it follows that fish must have smaller variance than other groups, (regardless of their actual values).

Alternatively, if this slope dependence on fish biomass is an artefact, it implies the fish models are overly sensitive to variations in their prey and are varying more in response than they should in reality. This appears due to fish having the largest range in biomass, spanning more than 2 decades, when other groups span < 1, and that fish occupy the entire upper part of the regression, since mammals are excluded from this analysis. It is possible that fish biomass exhibits greater variability than other groups over different regions of the ocean due to their aggregation and migratory behaviors, but it is not clear if that variation would be detected at the coarse grid scale of our global map data.

Energetic considerations

In this section we show data for the predator-prey and metabolism-body mass relations across a similar size range as the global ocean size-spectrum shown in Figs. 1 and 2. In Fig. S14 we show data for predator-prey mass ratios. Here we are considering individual level predator-prey body mass relations, rather than community level predator-prey biomass relations (*146*, *147*).

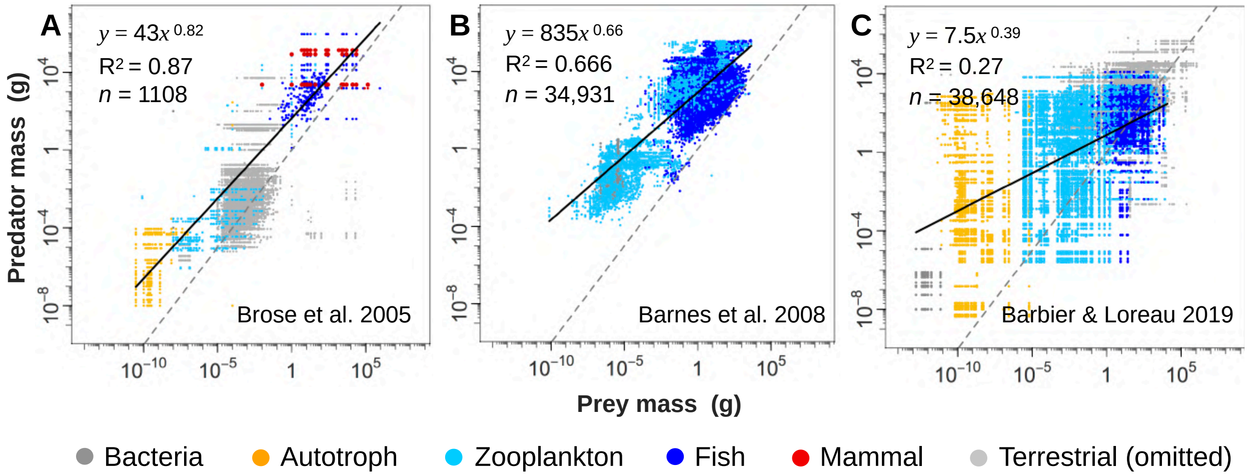

Fig. S14. Predator-prey mass relations. In all three plots, the typical mass (grams) of different species consumer-resource pairs are plotted. Only aquatic trophic interactions are colored (according to prey), for which the best fit regression (black line) equation is reported. The dashed grey line is the 1:1 line, meaning a predator consumes a prey of the same size, with points below the dashed line representing a smaller individual consuming a larger. Data are from **A**. Brose et al. 2005 (*52*, *148*), **B.** Barnes et al. 2008 (*53*), and **C.** Barbier & Loreau 2019 (*149*). The level of overlap between studies has not been investigated, but the patterns are all very highly dispersed.

Fig. S14 shows data from three sources separately (*53*, *148*, *149*), revealing in all cases that predator-prey mass ratios are not constant (exponent ≠ 1), as is widely assumed, and residual variation is extremely high, near six orders of magnitude in any given size class.

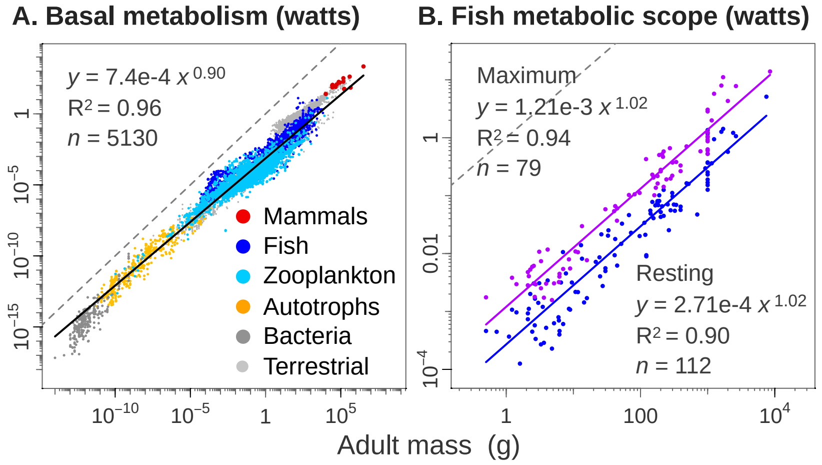

Fig. S15. Metabolic scaling. A. Basal metabolism (corrected to 15°C) is plotted from bacteria to whales (data from (*33*, *34*)), highlighting 5130 aquatic species (terrestrial species are colored light gray but omitted from the regression). **B.** Fish resting (blue) and maximum (violet) metabolism (corrected to 15°C) is shown for fish (data from (*56*)). These data were used to estimate the amount of energy associated with the lost biomass spectrum (pink striped area in Fig. 3 C).

Fig. S15 shows data for metabolism from (*33*, *34*, *56*), revealing that basal metabolism scales near 0.9 with body mass over all aquatic species, which is somewhat shallower than previous work has shown across similar size ranges (*33*, *34*, *55*), but still significantly steeper than ¾, as is often assumed. Across fish, both resting and maximum metabolism are nearly proportional to body mass. Note that we obtained slightly steeper slopes than are reported in the original paper that assembled these fish data (*56*) (0.948 for resting and 0.937 for maximum).

In all plots in Figs. S14 and S15 only aquatic species are colored, with terrestrial species shown in light grey and omitted from regressions and species counts (*n*). These individual trophic and metabolic relations are frequently thought to underpin the size spectrum in a number of theories, including (*19–24*, *26*, *28–30*, *150*, *151*) for predator-prey body mass ratios, and (*16–23*, *151*) for metabolism.

REFERENCES AND NOTES

1. R. W. Sheldon, A. Prakash, W. H. Sutcliffe Jr., The size distribution of particles in the ocean. *Limnol. Oceanogr.* **17**, 327–340 (1972).

2. W. G. Sprules, L. E. Barth, Surfing the biomass size spectrum: Some remarks on history, theory, and application. *Can. J. Fish. Aquat. Sci.* **73**, 477–495 (2015).

3. R. A. Quinones, T. Platt, J. Rodríguez, Patterns of biomass-size spectra from oligotrophic waters of the Northwest Atlantic. *Prog. Oceanogr.* **57**, 405–427 (2003).

4. J. Rodriguez, M. M. Mullin, Relation between biomass and body weight of plankton in a steady state oceanic ecosystem 1. *Limnol. Oceanogr.* **31**, 361–370 (1986).

5. A. Atkinson, M. K. S. Lilley, A. G. Hirst, A. J. McEvoy, G. A. Tarran, C. Widdicombe, E. S. Fileman, E. M. S. Woodward, K. Schmidt, T. J. Smyth, P. J. Somerfield, Increasing nutrient stress reduces the efficiency of energy transfer through planktonic size spectra. *Limnol. Oceanogr.* **66**, 422–437 (2021).

6. E. San Martin, X. Irigoien, R. P. Harris, A. López-Urrutia, M. V. Zubkov, J. L. Heywood, Variation in the transfer of energy in marine plankton along a productivity gradient in the Atlantic Ocean. *Limnol. Oceanogr.* **51**, 2084–2091 (2006).

7. P. R. Boudreau, L. M. Dickie, Biomass spectra of aquatic ecosystems in relation to fisheries yield. *Can. J. Fish. Aquat. Sci.* **49**, 1528–1538 (1992).

8. P. M. Yurista, D. L. Yule, M. Balge, J. D. VanAlstine, J. A. Thompson, A. E. Gamble, T. R. Hrabik, J. R. Kelly, J. D. Stockwell, M. R. Vinson, A new look at the Lake Superior biomass size spectrum. *Can. J. Fish. Aquat. Sci.* **71**, 1324–1333 (2014).

9. U. Gaedke, The size distribution of plankton biomass in a large lake and its seasonal variability. *Limnol. Oceanogr.* **37**, 1202–1220 (1992).

10. W. G. Sprules, A. P. Goyke, Size-based structure and production in the pelagia of Lakes Ontario and Michigan. *Can. J. Fish. Aquat. Sci.* **51**, 2603–2611 (1994).

11. H. Cyr, R. H. Peters, Biomass-size spectra and the prediction of fish biomass in lakes. *Can. J. Fish. Aquat. Sci.* **53**, 994–1006 (1996).

12. K. H. Andersen, *Fish Ecology, Evolution, and Exploitation: A New Theoretical Synthesis* (Princeton Univ. Press, 2019).

13. J. Guiet, J.-C. Poggiale, O. Maury, Modelling the community size-spectrum: Recent developments and new directions. *Ecol. Model.* **337**, 4–14 (2016).

14. K. H. Andersen, T. Berge, R. J. Gonçalves, M. Hartvig, J. Heuschele, S. Hylander, N. S. Jacobsen, C. Lindemann, E. A. Martens, A. B. Neuheimer, K. Olsson, A. Palacz, A. E. F. Prowe, J. Sainmont, S. J. Traving, A. W. Visser, N. Wadhwa, T. Kiørboe, Characteristic sizes of life in the oceans, from bacteria to whales. *Ann. Rev. Mar. Sci.* **8**, 217–241 (2016).

15. J. L. Blanchard, R. F. Heneghan, J. D. Everett, R. Trebilco, A. J. Richardson, From bacteria to whales: Using functional size spectra to model marine ecosystems. *Trends Ecol. Evol.* **32**, 174–186 (2017).

16. T. Platt, K. Denman, Organisation in the pelagic ecosystem. *Helgoländer Wissenschaftliche Meeresuntersuchungen.* **30**, 575–581 (1977).

17. W. Silvert, T. Platt, Energy flux in the pelagic ecosystem: A time-dependent equation. *Limnol. Oceanogr.* **23**, 813–816 (1978).

18. K. H. Andersen, J. E. Beyer, Asymptotic size determines species abundance in the marine size spectrum. *Am. Nat.* **168**, 54–61 (2006).

19. R. W. Sheldon, W. H. Sutcliffe Jr., M. A. Paranjape, Structure of pelagic food chain and relationship between plankton and fish production. *J. Fish. Board Canada* **34**, 2344–2353 (1977).

20. S. Jennings, K. Collingridge, Predicting consumer biomass, size-structure, production, catch potential, responses to fishing and associated uncertainties in the world's marine ecosystems. *PLOS ONE* **10**, e0133794 (2015).

21. S. Jennings, S. Mackinson, Abundance-body mass relationships in size-structured food webs. *Ecol. Lett.* **6**, 971–974 (2003).

22. S. R. Kerr, Theory of size distribution in ecological communities. *J. Fish. Board Canada* **31**, 1859–1862 (1974).

23. R. Trebilco, J. K. Baum, A. K. Salomon, N. K. Dulvy, Ecosystem ecology: Size-based constraints on the pyramids of life. *Trends Ecol. Evol.* **28**, 423–431 (2013).

24. U. Borgmann, Models on the slope of, and biomass flow up, the biomass size spectrum. *Can. J. Fish. Aquat. Sci.* **44**, s136–s140 (1987).

25. J. Camacho, R. V. Solé, Scaling in ecological size spectra. *EPL (Europhysics Letters)* **55**, 774–780 (2001).

26. T. M. Canales, R. Law, J. L. Blanchard, Shifts in plankton size spectra modulate growth and coexistence of anchovy and sardine in upwelling systems. *Can. J. Fish. Aquat. Sci.* **73**, 611–621 (2016).

27. M. Zhou, M. E. Huntley, Population dynamics theory of plankton based on biomass spectra. *Mar. Ecol. Prog. Ser.* **159**, 61–73 (1997).

28. M. Hartvig, K. H. Andersen, J. E. Beyer, Food web framework for size-structured populations. *J. Theor. Biol.* **272**, 113–122 (2011).

29. E. Benoıt, M.-J. Rochet, A continuous model of biomass size spectra governed by predation and the effects of fishing on them. *J. Theor. Biol.* **226**, 9–21 (2004).

30. M. L. Thiebaux, L. M. Dickie, Models of aquatic biomass size spectra and the common structure of their solutions. *J. Theor. Biol.* **159**, 147–161 (1992).

31. R. H. Peters, *The Ecological Implications Of Body Size* (Cambridge Studies in Ecology, Cambridge Univ. Press, Cambridge, ed. 1, 1983).

32. J. H. Brown, J. F. Gillooly, A. P. Allen, V. M. Savage, G. B. West, Toward a metabolic theory of

ecology. *Ecology* **85**, 1771–1789 (2004).

33. T. Kiørboe, A. G. Hirst, Shifts in mass scaling of respiration, feeding, and growth rates across life-form transitions in marine pelagic organisms. *Am. Nat.* **183**, E118–E130 (2014).

34. I. A. Hatton, A. P. Dobson, D. Storch, E. D. Galbraith, M. Loreau, Linking scaling laws across eukaryotes. *Proc. Natl. Acad. Sci.* **116**, 21616–21622 (2019).

35. R. F. Heneghan, I. A. Hatton, E. D. Galbraith, Climate change impacts on marine ecosystems through the lens of the size spectrum. *Emerging Top. Life Sci.* **3**, 233–243 (2019).

36. E. Tekwa, K. Catalano, A. Bazzicalupo, M. Pinsky, *The Global Body Size Biomass Spectrum is Multimodal* (2021); Preprint at www.researchsquare.com/article/rs-144241/v1.

37. N. Daan, H. Gislason, J. G. Pope, J. C. Rice, Changes in the North Sea fish community: Evidence of indirect effects of fishing? *ICES J. Mar. Sci.* **62**, 177–188 (2005).

38. J. G. Pope, B. J. Knights, Comparison of length distributions of combined catches of all demersal fishes in surveys in the North Sea and at Faroe Bank. *Can. Spec. Publ. Fish. Aquat. Sci.* **59**, 116–118 (1982).

39. D. A. Carozza, D. Bianchi, E. D. Galbraith, Formulation, general features and global calibration of a bioenergetically-constrained fishery model. *PLOS ONE* **12**, e0169763 (2017).

40. C. M. Petrik, C. A. Stock, K. H. Andersen, P. D. van Denderen, J. R. Watson, Bottom-up drivers of global patterns of demersal, forage, and pelagic fishes. *Prog. Oceanogr.* **176**, 102124 (2019).

41. Y. M. Bar-On, R. Phillips, R. Milo, The biomass distribution on Earth. *Proc. Natl. Acad. Sci.* **115**, 6506–6511 (2018).

42. Y. M. Bar-On, R. Milo, The biomass composition of the oceans: A blueprint of our blue planet. *Cell* **179**, 1451–1454 (2019).

43. E. Buitenhuis, M. Vogt, R. Moriarty, N. Bednarsek, S. C. Doney, K. Leblanc, C. Le Quéré, Y.-W. Luo, C. O'Brien, T. O'Brien, J. Peloquin, R. Schiebel, C. Swan, MAREDAT: Towards a world atlas

of MARine Ecosystem DATa. *Earth Syst. Sci. Data* **5**, 227–239 (2013).

44. Further details are provided in Supplementary Materials linked to the online article. Data and code are available at: https://doi.org/10.5281/zenodo.5520055.

45. V. Christensen, M. Coll, C. Piroddi, J. Steenbeek, J. Buszowski, D. Pauly, A century of fish biomass decline in the ocean. *Mar. Ecol. Prog. Ser.* **512**, 155–166 (2014).

46. B. Worm, T. A. Branch, The future of fish. *Trends Ecol. Evol.* **27**, 594–599 (2012).

47. IUCN 2020, The IUCN Red List of Threatened Species, Version 2020–1, *IUCN Red List of Threatened Species* (2020); www.iucnredlist.org/en.

48. L. B. Christensen, "Marine mammal populations: Reconstructing historical abundances at the global scale," M.Sc. thesis, Fisheries Centre, University of British Columbia (2006).

49. L. Kwiatkowski, O. Aumont, L. Bopp, Consistent trophic amplification of marine biomass declines under climate change. *Glob. Chang. Biol.* **25**, 218–229 (2019).

50. H. K. Lotze, D. P. Tittensor, A. Bryndum-Buchholz, T. D. Eddy, W. W. Cheung, E. D. Galbraith, M. Barange, N. Barrier, D. Bianchi, J. L. Blanchard, L. Bopp, M. Büchner, C. M. Bulman, D. A. Carozza, V. Christensen, M. Coll, J. P. Dunne, E. A. Fulton, S. Jennings, M. C. Jones, S. Mackinson, O. Maury, S. Niiranen, R. Oliveros-Ramos, T. Roy, J. A. Fernandes, J. Schewe, Y.-J. Shin, T. A. M. Silva, J. Steenbeek, C. A. Stock, P. Verley, J. Volkholz, N. D. Walker, B. Worm, Global ensemble projections reveal trophic amplification of ocean biomass declines with climate change. *Proc. Natl. Acad. Sci. U.S.A.* **116**, 12907–12912 (2019).

51. J. M. Grady, B. S. Maitner, A. S. Winter, K. Kaschner, D. P. Tittensor, S. Record, F. A. Smith, A. M. Wilson, A. I. Dell, P. L. Zarnetske, H. J. Wearing, B. Alfaro, J. H. Brown, Metabolic asymmetry and the global diversity of marine predators. *Science* **363**, eaat4220 (2019).

52. U. Brose, T. Jonsson, E. L. Berlow, P. Warren, C. Banasek-Richter, L.-F. Bersier, J. L. Blanchard, T. Brey, S. R. Carpenter, M.-F. C. Blandenier, L. Cushing, H. A. Dawah, T. Dell, F. Edwards, S. Harper-Smith, U. Jacob, M. E. Ledger, N. D. Martinez, J. Memmott, K. Mintenbeck, J. K. Pinnegar,

B. C. Rall, T. S. Rayner, D. C. Reuman, L. Ruess, W. Ulrich, R. J. Williams, G. Woodward, J. E. Cohen, Consumer-resource body-size relationships in natural food webs. *Ecology* **87**, 2411–2417 (2006).

53. C. Barnes, D. M. Bethea, R. D. Brodeur, J. Spitz, V. Ridoux, C. Pusineri, B. C. Chase, M. E. Hunsicker, F. Juanes, A. Kellermann, J. Lancaster, F. Ménard, F.-X. Bard, P. Munk, J. K. Pinnegar, F. S. Scharf, R. A. Rountree, K. I. Stergiou, C. Sassa, A. Sabates, S. Jennings, Predator and prey body sizes in marine food webs. *Ecology* **89**, 881 (2008).

54. R. F. Heneghan, J. D. Everett, P. Sykes, S. D. Batten, M. Edwards, K. Takahashi, I. M. Suthers, J. L. Blanchard, A. J. Richardson, A functional size-spectrum model of the global marine ecosystem that resolves zooplankton composition. *Ecol. Model.* **435**, 109265 (2020).

55. A. M. Makarieva, V. G. Gorshkov, B.-L. Li, S. L. Chown, P. B. Reich, V. M. Gavrilov, Mean mass-specific metabolic rates are strikingly similar across life's major domains: Evidence for life's metabolic optimum. *Proc. Natl. Acad. Sci. U.S.A.* **105**, 16994–16999 (2008).

56. S. S. Killen, D. S. Glazier, E. L. Rezende, T. D. Clark, D. Atkinson, A. S. T. Willener, L. G. Halsey, Ecological influences and morphological correlates of resting and maximal metabolic rates across teleost fish species. *Am. Nat.* **187**, 592–606 (2016).

57. C. T. Darimont, C. H. Fox, H. M. Bryan, T. E. Reimchen, The unique ecology of human predators. *Science* **349**, 858–860 (2015).

58. D. Pauly, D. Zeller, Catch reconstructions reveal that global marine fisheries catches are higher than reported and declining. *Nat. Commun.* **7**, 10244 (2016).

59. M. Newman, Power laws, Pareto distributions and Zipf's law. *Contemporary Phys.* **46**, 323–351 (2005).

60. C. H. Wigington, D. Sonderegger, C. P. D. Brussaard, A. Buchan, J. F. Finke, J. A. Fuhrman, J. T. Lennon, M. Middelboe, C. A. Suttle, C. Stock, W. H. Wilson, K. E. Wommack, S. W. Wilhelm, J. S. Weitz, Re-examination of the relationship between marine virus and microbial cell abundances. *Nat. Microbiol.* **1**, 15024 (2016).

61. E. T. Buitenhuis, W. K. W. Li, M. W. Lomas, D. M. Karl, M. R. Landry, S. Jacquet, Global distributions of picoheterothrophs (Bacteria and Archaea) abundance and biomass-Gridded data product (NetCDF)-Contribution to the MAREDAT World Ocean Atlas of Plankton Functional Types, in *PANGAEA* (2012); https://doi.org/10.1594/PANGAEA.779142.

62. E. Lara, D. Vaqué, E. L. Sà, J. A. Boras, A. Gomes, E. Borrull, C. Díez-Vives, E. Teira, M. C. Pernice, F. C. Garcia, I. Forn, Y. M. Castillo, A. Peiró, G. Salazar, X. A. G. Morán, R. Massana, T. S. Catalá, G. M. Luna, S. Agustí, M. Estrada, J. M. Gasol, C. M. Duarte, Unveiling the role and life strategies of viruses from the surface to the dark ocean. *Sci. Adv.* **3**, e1602565 (2017).

63. T. D. O'Brien, *COPEPOD: The Global Plankton Database, An Overview of the 2014 Database Contents, Processing Methods, and Access Interface* (U.S. Dep. Commerce, NOAA Tech. Memo, 2014).

64. E. T. Buitenhuis, R. B. Rivkin, S. Sailley, C. Le Quéré, Biogeochemical fluxes through microzooplankton. *Global Biogeochem. Cycles* **24**, GB4015 (2010).

65. T. Biard, L. Stemmann, M. Picheral, N. Mayot, P. Vandromme, H. Hauss, G. Gorsky, L. Guidi, R. Kiko, F. Not, In situ imaging reveals the biomass of giant protists in the global ocean. *Nature* **532**, 504–507 (2016).

66. R. Moriarty, Global distributions of epipelagic macrozooplankton abundance and biomass - Gridded data product (NetCDF) - Contribution to the MAREDAT World Ocean Atlas of Plankton Functional Types, in *PANGAEA* (2012); https://doi.org/10.1594/PANGAEA.777398.

67. K. Kaschner, Modelling and mapping resource overlap between marine mammals and fisheries on a global scale, Ph.D. thesis, University of British Columbia (2004).

68. M. Omorii, T. Ikeda, *Methods in Marine Zooplankton Ecology* (John Wiley & Sons, New York, 1984).

69. C. De Vargas, S. Audic, N. Henry, J. Decelle, F. Mahé, R. Logares, E. Lara, C. Berney, N. Le Bescot, I. Probert, M. Carmichael, J. Poulain, S. Romac, S. Colin, J.-M. Aury, L. Bittner, S. Chaffron, M. Dunthorn, S. Engelen, O. Flegontova, L. Guidi, A. Horák, O. Jaillon, G. Lima-Mendez, J. Lukeš,

S. Malviya, R. Morard, M. Mulot, E. Scalco, R. Siano, F. Vincent, A. Zingone, C. Dimier, M. Picheral, S. Searson, S. Kandels-Lewis, T. O. Coordinators, S. G. Acinas, P. Bork, C. Bowler, G. Gorsky, N. Grimsley, P. Hingamp, D. Iudicone, F. Not, H. Ogata, S. Pesant, J. Raes, M. E. Sieracki, S. Speich, L. Stemmann, S. Sunagawa, J. Weissenbach, P. Wincker, E. Karsenti, Eukaryotic plankton diversity in the sunlit ocean. *Science* **348**, 1261605 (2015).

70. R. Moriarty, T. D. O'Brien, Distribution of mesozooplankton biomass in the global ocean. *Earth Syst. Sci. Data* **5**, 45–55 (2013).

71. R. Moriarty, E. T. Buitenhuis, C. Le Quéré, M.-P. Gosselin, Distribution of known macrozooplankton abundance and biomass in the global ocean. *Earth Syst. Sci. Data* **5**, 241–257 (2013).

72. J. Uitz, H. Claustre, A. Morel, S. B. Hooker, Vertical distribution of phytoplankton communities in open ocean: An assessment based on surface chlorophyll. *J. Geophys. Res. Oceans* **111**, C08005 (2006).

73. E. Marañón, P. Cermeño, M. Huete-Ortega, D. C. López-Sandoval, B. Mouriño-Carballido, T. Rodríguez-Ramos, Resource supply overrides temperature as a controlling factor of marine phytoplankton growth. *PLOS ONE* **9**, e99312 (2014).

74. A. D. King, M. G. Donat, E. M. Fischer, E. Hawkins, L. V. Alexander, D. J. Karoly, A. J. Dittus, S. C. Lewis, S. E. Perkins, The timing of anthropogenic emergence in simulated climate extremes. *Environ. Res. Lett.* **10**, 094015 (2015).

75. R. Proud, N. O. Handegard, R. J. Kloser, M. J. Cox, A. S. Brierley, From siphonophores to deep scattering layers: Uncertainty ranges for the estimation of global mesopelagic fish biomass. *ICES J. Mar. Sci.* **76**, 718–733 (2019).

76. K. R. Turpie, R. E. Eplee Jr., B. A. Franz, C. Del Castillo, in *Ocean Sensing and Monitoring VI* (International Society for Optics and Photonics, 2014), vol. 9111, p. 911103.

77. A. J. Richardson, A. W. Walne, A. W. G. John, T. D. Jonas, J. A. Lindley, D. W. Sims, D. Stevens, M. Witt, Using continuous plankton recorder data. *Prog. Oceanogr.* **68**, 27–74 (2006).

78. D. I. Warton, I. J. Wright, D. S. Falster, M. Westoby, Bivariate line-fitting methods for allometry. *Biol. Rev. Camb. Philos. Soc.* **81**, 259–291 (2006).

79. R. Hanel, B. Corominas-Murtra, B. Liu, S. Thurner, Fitting power-laws in empirical data with estimators that work for all exponents. *PLOS ONE* **12**, e0170920 (2017).

80. L. Bopp, L. Resplandy, J. C. Orr, S. C. Doney, J. P. Dunne, M. Gehlen, P. Halloran, C. Heinze, T. Ilyina, R. Séférian, J. Tjiputra, M. Vichi, Multiple stressors of ocean ecosystems in the 21st century: Projections with CMIP5 models. *Biogeosciences* **10**, 6225–6245 (2013).

81. T. Brey, C. Müller-Wiegmann, Z. M. C. Zittier, W. Hagen, Body composition in aquatic organisms—A global data bank of relationships between mass, elemental composition and energy content. *J. Sea Res.* **64**, 334–340 (2010).

82. R. J. W. Brewin, S. Sathyendranath, T. Hirata, S. J. Lavender, R. M. Barciela, N. J. Hardman-Mountford, A three-component model of phytoplankton size class for the Atlantic Ocean. *Ecol. Model.* **221**, 1472–1483 (2010).

83. Z. Lee, A. Weidemann, J. Kindle, R. Arnone, K. L. Carder, C. Davis, Euphotic zone depth: Its derivation and implication to ocean-color remote sensing. *J. Geophys. Res. Oceans* **112**, C03009 (2007).

84. J. Holte, L. Talley, A new algorithm for finding mixed layer depths with applications to Argo data and Subantarctic Mode Water formation. *J. Atmos. Oceanic Tech.* **26**, 1920–1939 (2009).

85. R. W. Sheldon, T. R. Parsons, A continuous size spectrum for particulate matter in the sea. *J. Fish. Board Canada* **24**, 909–915 (1967).

86. W. B. Whitman, D. C. Coleman, W. J. Wiebe, Prokaryotes: The unseen majority. *PNAS.* **95**, 6578–6583 (1998).

87. U. Gaedke, A. Seifried, R. Adrian, Biomass size spectra and plankton diversity in a shallow eutrophic lake. *Int. Rev. Hydrobiol.* **89**, 1–20 (2004).

88. J. Tittel, B. Zippel, W. Geller, J. Seeger, Relationships between plankton community structure and plankton size distribution in lakes of northern Germany. *Limnol. Oceanogr.* **43**, 1119–1132 (1998).

89. B. Vidondo, Y. T. Prairie, J. M. Blanco, C. M. Duarte, Some aspects of the analysis of size spectra in aquatic ecology. *Limnol. Oceanogr.* **42**, 184–192 (1997).

90. A. Cózar, C. M. García, J. A. Gálvez, Analysis of plankton size spectra irregularities in two subtropical shallow lakes (Esteros del Iberá, Argentina). *Can. J. Fish. Aquat. Sci.* **60**, 411–420 (2003).

91. C. da Rocha Marcolin, S. Schultes, G. A. Jackson, R. M. Lopes, Plankton and seston size spectra estimated by the LOPC and ZooScan in the Abrolhos Bank ecosystem (SE Atlantic). *Cont. Shelf Res.* **70**, 74–87 (2013).

92. Z. Witek, A. Krajewska-Soltys, Some examples of the epipelagic plankton size structure in high latitude oceans. *J. Plankton Res.* **11**, 1143–1155 (1989).

93. C. García-Muñoz, C. M. García, L. M. Lubián, Á. López-Urrutia, S. Hernández-León, J. Ameneiro, Metabolic state along a summer north–south transect near the Antarctic Peninsula: A size spectra approach. *J. Plankton Res.* **36**, 1074–1091 (2014).

94. C. Frangoulis, M. Grigoratou, T. Zoulias, C. C. Hannides, M. Pantazi, S. Psarra, I. Siokou, Expanding zooplankton standing stock estimation from meso-to metazooplankton: A case study in the N. Aegean Sea (Mediterranean Sea). *Cont. Shelf Res.* **149**, 151–161 (2017).

95. G. A. Tarling, G. Stowasser, P. Ward, A. J. Poulton, M. Zhou, H. J. Venables, R. A. R. McGill, E. J. Murphy, Seasonal trophic structure of the Scotia Sea pelagic ecosystem considered through biomass spectra and stable isotope analysis. *Deep-Sea Res. II Top. Stud. Oceanogr.* **59**, 222–236 (2012).

96. W. G. Sprules, Ecological change in Great Lakes communities—A matter of perspective. *Can. J. Fish. Aquat. Sci.* **65**, 1–9 (2008).

97. S. Kaartvedt, A. Staby, D. L. Aksnes, Efficient trawl avoidance by mesopelagic fishes causes large underestimation of their biomass. *Mar. Ecol. Prog. Ser.* **456**, 1–6 (2012).

98. J. D. Everett, M. E. Baird, P. Buchanan, C. Bulman, C. Davies, R. Downie, C. Griffiths, R. Heneghan, R. J. Kloser, L. Laiolo, A. Lara-Lopez, H. Lozano-Montes, R. J. Matear, F. M. Ennulty, B. Robson, W. Rochester, J. Skerratt, J. A. Smith, J. Strzelecki, I. M. Suthers, K. M. Swadling, P. van Ruth, A. J. Richardson, Modeling what we sample and sampling what we model: Challenges for zooplankton model assessment. *Front. Mar. Sci.* **4**, 77 (2017).

99. M. E. Sieracki, E. M. Haugen, T. L. Cucci, Overestimation of heterotrophic bacteria in the Sargasso Sea: Direct evidence by flow and imaging cytometry. *Deep-Sea Res. I Oceanogr. Res. Pap.* **42**, 1399–1409 (1995).

100. NASA Goddard Space Flight Center, Ocean Ecology Laboratory, Ocean Biology Processing Group, Moderate-resolution Imaging Spectroradiometer (MODIS) Aqua Chlorophyll and Sea-Surface Temperature Data; NASA OB.DAAC, Greenbelt, MD, USA.

101. GEBCO Compilation Group (2020) The General Bathymetric Chart of the Oceans 2020 Grid (doi:10.5285/a29c5465-b138-234d-e053-6c86abc040b9).

102. K. G. Lloyd, M. K. May, R. T. Kevorkian, A. D. Steen, Meta-analysis of quantification methods shows that archaea and bacteria have similar abundances in the subseafloor. *Appl. Environ. Microbiol.* **79**, 7790–7799 (2013).

103. J. Arístegui, J. M. Gasol, C. M. Duarte, G. J. Herndld, Microbial oceanography of the dark ocean's pelagic realm. *Limnol. Oceanogr.* **54**, 1501–1529 (2009).

104. K. Gundersen, M. Heldal, S. Norland, D. A. Purdie, A. H. Knap, Elemental C, N, and P cell content of individual bacteria collected at the Bermuda Atlantic Time-series Study (BATS) site. *Limnol. Oceanogr.* **47**, 1525–1530 (2002).

105. K. M. Fagerbakke, M. Heldal, S. Norland, Content of carbon, nitrogen, oxygen, sulfur and phosphorus in native aquatic and cultured bacteria. *Aquat. Microb. Ecol.* **10**, 15–27 (1996).

106. S. Lee, J. A. Fuhrman, Relationships between biovolume and biomass of naturally derived marine bacterioplankton. *Appl. Environ. Microbiol.* **53**, 1298–1303 (1987).

107. M. Simon, F. Azam, Protein content and protein synthesis rates of planktonic marine bacteria. *Marine Ecology Progress Series. Oldendorf.* **51**, 201–213 (1989).

108. M. Troussellier, M. Bouvy, C. Courties, C. Dupuy, Variation of carbon content among bacterial species under starvation condition. *Aquat. Microb. Ecol.* **13**, 113–119 (1997).

109. C. A. Carlson, H. W. Ducklow, T. D. Sleeter, Stocks and dynamics of bacterioplankton in the northwestern Sargasso Sea. *Deep-Sea Res. II Top. Stud. Oceanogr.* **43**, 491–515 (1996).

110. D. Šantić, N. Krstulović, M. Šolić, Comparison of flow cytometric and epifluorescent counting methods for marine heterotrophic bacteria. *Acta Adriat.* **48**, 107–114 (2007).

111. R. Fukuda, H. Ogawa, T. Nagata, I. Koike, Direct determination of carbon and nitrogen contents of natural bacterial assemblages in marine environments. *Appl. Environ. Microbiol.* **64**, 3352–3358 (1998).

112. S. Sathyendranath, J. Aiken, S. Alvain, R. Barlow, H. Bouman, A. Bracher, R. Brewin, A. Bricaud, C. W. Brown, A. M. Ciotti, in *Reports of the International Ocean-Colour Coordinating Group (IOCCG); 15* (International Ocean-Colour Coordinating Group, 2014), pp. 1–156.

113. A. Morel, J.-F. Berthon, Surface pigments, algal biomass profiles, and potential production of the euphotic layer: Relationships reinvestigated in view of remote-sensing applications. *Limnol. Oceanogr.* **34**, 1545–1562 (1989).

114. S. Agustí, J. I. González-Gordillo, D. Vaqué, M. Estrada, M. I. Cerezo, G. Salazar, J. M. Gasol, C. M. Duarte, Ubiquitous healthy diatoms in the deep sea confirm deep carbon injection by the biological pump. *Nat. Commun.* **6**, 7608 (2015).

115. J. Gjøsæter, K. Kawaguchi, *A Review of the World Resources of Mesopelagic Fish* (Food & Agriculture Org., 1980).

116. L. N. Eduardo, M. M. Mincarone, B. T. Villarins, T. Frédou, A. S. Lira, A. Bertrand, F. Lucena-Frédou, Length–weight relationships of eleven mesopelagic fishes from oceanic islands of the Southwestern Tropical Atlantic. *J. Appl. Ichthyol.* **35**, 605–607 (2019).

117. P. C. Escobar-Flores, R. L. O'Driscoll, J. C. Montgomery, Y. Ladroit, S. Jendersie, Estimates of density of mesopelagic fish in the Southern Ocean derived from bulk acoustic data collected by ships of opportunity. *Polar Biol.* **43**, 43–61 (2020).

118. C. López-Pérez, M. P. Olivar, P. A. Hulley, V. M. Tuset, Length–weight relationships of mesopelagic fishes from the equatorial and tropical Atlantic waters: Influence of environment and body shape. *J. Fish Biol.* **96**, 1388–1398 (2020).

119. WoRMS Editorial Board, World Register of Marine Species, Available from www.marinespecies.org at VLIZ, Accessed 2021-06-13 (2021), https://doi.org/10.14284/170.

120. K. E. Jones, J. Bielby, M. Cardillo, S. A. Fritz, J. O'Dell, C. D. L. Orme, K. Safi, W. Sechrest, E. H. Boakes, C. Carbone, C. Connolly, M. J. Cutts, J. K. Foster, R. Grenyer, M. Habib, C. A. Plaster, S. A. Price, E. A. Rigby, J. Rist, A. Teacher, O. R. P. Bininda-Emonds, J. L. Gittleman, G. M. Mace, A. Purvis, PanTHERIA: A species-level database of life history, ecology, and geography of extant and recently extinct mammals. *Ecology* **90**, 2648–2648 (2009).

121. S. Pompa, P. R. Ehrlich, G. Ceballos, Global distribution and conservation of marine mammals. *Proc. Natl. Acad. Sci. U.S.A.* **108**, 13600–13605 (2011).

122. J. Damuth, Population density and body size in mammals. *Nature* **290**, 699–700 (1981).

123. J. Damuth, Interspecific allometry of population density in mammals and other animals: The independence of body mass and population energy-use. *Biol. J. Linn. Soc.* **31**, 193–246 (1987).

124. C. M. Duarte, S. Agusti, E. Barbier, G. L. Britten, J. C. Castilla, J.-P. Gattuso, R. W. Fulweiler, T. P. Hughes, N. Knowlton, C. E. Lovelock, H. K. Lotze, M. Predragovic, E. Poloczanska, C. Roberts, B. Worm, Rebuilding marine life. *Nature* **580**, 39–51 (2020).

125. D. Bianchi, E. D. Galbraith, D. Carozza, in *2018 Ocean Sciences Meeting* (AGU, 2018).

126. J. B. Jackson, M. X. Kirby, W. H. Berger, K. A. Bjorndal, L. W. Botsford, B. J. Bourque, R. H. Bradbury, R. Cooke, J. Erlandson, J. A. Estes, T. P. Hughes, S. Kidwell, C. B. Lange, H. S. Lenihan, J. M. Pandolfi, C. H. Peterson, R. S. Steneck, M. J. Tegner, R. R. Warner, Historical overfishing and

the recent collapse of coastal ecosystems. *Science* **293**, 629–637 (2001).

127. B. S. Halpern, S. Walbridge, K. A. Selkoe, C. V. Kappel, F. Micheli, C. D'Agrosa, J. F. Bruno, K. S. Casey, C. Ebert, H. E. Fox, R. Fujita, D. Heinemann, H. S. Lenihan, E. M. P. Madin, M. T. Perry, E. R. Selig, M. Spalding, R. Steneck, R. Watson, A global map of human impact on marine ecosystems. *Science* **319**, 948–952 (2008).

128. P. Alexander, C. Brown, A. Arneth, J. Finnigan, D. Moran, M. D. A. Rounsevell, Losses, inefficiencies and waste in the global food system. *Agr. Syst.* **153**, 190–200 (2017).

129. H. K. Lotze, B. Worm, Historical baselines for large marine animals. *Trends Ecol. Evol.* **24**, 254–262 (2009).

130. J. T. Thorson, J. M. Cope, T. A. Branch, O. P. Jensen, Spawning biomass reference points for exploited marine fishes, incorporating taxonomic and body size information. *Can. J. Fish. Aquat. Sci.* **69**, 1556–1568 (2012).

131. E. D. Galbraith, D. A. Carozza, D. Bianchi, A coupled human-Earth model perspective on long-term trends in the global marine fishery. *Nat. Commun.* **8**, 14884 (2017).

132. D. Bianchi, D. A. Carozza, E. D. Galbraith, J. Guiet, T. DeVries, Estimating global biomass and biogeochemical cycling of marine fish with and without fishing. *Science Advances* **7**, eabd7554 (2021).

133. M.-J. Rochet, E. Benoît, Fishing destabilizes the biomass flow in the marine size spectrum. *Proc. R. Soc. B Biol. Sci.* **279**, 284–292 (2012).

134. S. Jennings, J. L. Blanchard, Fish abundance with no fishing: Predictions based on macroecological theory. *J. Anim. Ecol.* **73**, 632–642 (2004).

135. G. Bianchi, H. Gislason, K. Graham, L. Hill, X. Jin, K. Koranteng, S. Manickchand-Heileman, I. Paya, K. Sainsbury, F. Sanchez, K. Zwanenburg, Impact of fishing on size composition and diversity of demersal fish communities. *ICES J. Mar. Sci.* **57**, 558–571 (2000).

136. J. P. Robinson, I. D. Williams, A. M. Edwards, J. McPherson, L. Yeager, L. Vigliola, R. E. Brainard, J. K. Baum, Fishing degrades size structure of coral reef fish communities. *Glob. Chang. Biol.* **23**, 1009–1022 (2017).

137. H. A. Simon, On a class of skew distribution functions. *Biometrika* **42**, 425–440 (1955).

138. G. K. Zipf, *Human Behavior and the Principle of Least Effort: An Introduction to Human Ecology* (Addison-Wesley, New York, 1949).

139. A. Clauset, C. R. Shalizi, M. E. J. Newman, Power-law distributions in empirical data. *SIAM review*. **51**, 661–703 (2009).

140. A. Deluca, Á. Corral, Fitting and goodness-of-fit test of non-truncated and truncated power-law distributions. *Acta Geophys.* **61**, 1351–1394 (2013).

141. I. Voitalov, P. van der Hoorn, R. van der Hofstad, D. Krioukov, Scale-free networks well done. *Phys. Rev. Res.*. **1**, 033034 (2019).

142. J. M. Blanco, F. Echevarría, C. M. García, Dealing with size-spectra: Some conceptual and mathematical problems. *Scientia Marina (Espana)* **58**, 17–29 (1994).

143. Á. Corral, I. Serra, R. Ferrer-i-Cancho, Distinct flavors of Zipf's law and its maximum likelihood fitting: Rank-size and size-distribution representations. *Phys. Rev. E.* **102**, 052113 (2020).

144. D. I. Warton, R. A. Duursma, D. S. Falster, S. Taskinen, smatr 3–an R package for estimation and inference about allometric lines. *Methods Ecol. Evol.* **3**, 257–259 (2012).

145. M. Emmrich, S. Brucet, D. Ritterbusch, T. Mehner, Size spectra of lake fish assemblages: Responses along gradients of general environmental factors and intensity of lake-use. *Freshwater Biol.* **56**, 2316–2333 (2011).

146. I. A. Hatton, K. S. McCann, J. M. Fryxell, T. J. Davies, M. Smerlak, A. R. E. Sinclair, M. Loreau, The predator-prey power law: Biomass scaling across terrestrial and aquatic biomes. *Science* **349**, aac6284 (2015).

147. I. Hatton, in *Unsolved Problems in Ecology*, A. Dobson, R. Holt, D. Tilman Eds. (Princeton Univ. Press., Princeton, 2020), p. 203; https://doi.org/10.2307/j.ctvs9fh2n.

148. U. Brose, L. Cushing, E. L. Berlow, T. Jonsson, C. Banasek-Richter, L.-F. Bersier, J. L. Blanchard, T. Brey, S. R. Carpenter, M.-F. C. Blandenier, J. E. Cohen, H. A. Dawah, T. Dell, F. Edwards, S. Harper-Smith, U. Jacob, R. A. Knapp, M. E. Ledger, J. Memmott, K. Mintenbeck, J. K. Pinnegar, B. C. Rall, T. Rayner, L. Ruess, W. Ulrich, P. Warren, R. J. Williams, G. Woodward, P. Yodzis, N. D. Martinez, Body sizes of consumers and their resources. *Ecology* **86**, 2545–2545 (2005).

149. M. Barbier, M. Loreau, Pyramids and cascades: A synthesis of food chain functioning and stability. *Ecol. Lett.* **22**, 405–419 (2019).

150. R. Law, M. J. Plank, A. James, J. L. Blanchard, Size-spectra dynamics from stochastic predation and growth of individuals. *Ecology*. **90**, 802–811 (2009).

151. A. G. Rossberg, A complete analytic theory for structure and dynamics of populations and communities spanning wide ranges in body size. *Adv. Ecol. Res.* **46**, 427–521 (2012).
